# Autologous transplantation of P63^+^ lung progenitor cells in patients with bronchiectasis: A randomized, single-blind, controlled trial

**DOI:** 10.1016/j.xcrm.2024.101819

**Published:** 2024-11-19

**Authors:** Jiayang Yan, Weipan Zhang, Yun Feng, Xuefei Liu, Lingyun Niu, Yi Guo, Ling Zhou, Mengmeng Shi, Caixia Di, Qiurui Zhang, Xiaofei Wang, Jianping Zhou, Ranran Dai, Lei Ni, Zhiyao Bao, Tianli Yan, Yun Hu, Ping Wang, Ting Zhang, Min Zhou, Wei Zuo, Jieming Qu

**Affiliations:** 1 Department of Pulmonary and Critical Care Medicine, Ruijin Hospital, Shanghai Jiao Tong University School of Medicine, Shanghai 200025, China; 2 Institute for Regenerative Medicine, State Key Laboratory of Cardiology and Medical Innovation Center, Shanghai East Hospital, School of Medicine, Tongji University, Shanghai 200092, China; 3 Institute of Respiratory Diseases, Shanghai Jiao Tong University School of Medicine, Shanghai 200025, China; 4 Shanghai Key Laboratory of Emergency Prevention, Diagnosis and Treatment of Respiratory Infectious Diseases, Shanghai 200025, China; 5 Super Organ R&D Center, Regend Therapeutics, Shanghai 201318, China

## Abstract

Non-cystic fibrosis bronchiectasis is a progressive respiratory disease with limited treatment options, prompting the exploration of regenerative therapies. This study investigates the safety and efficacy of autologous P63^+^ progenitor cell transplantation in a randomized, single-blind, controlled, phase 1/2 trial. Thirty-seven patients receive bronchoscopic airway clearance (B-ACT) (*n* = 19) or B-ACT plus P63^+^ progenitor cells (*n* = 18). Results show that compared to the control group, the change in D_LCO_ levels from baseline to 24 weeks post therapy is significantly higher in the cell treatment group (*p* value = 0.039). Furthermore, the patients in the cell treatment group demonstrate significantly reduced lung damaged area, improved SGRQ score, and ameliorated BSI and FACED scores within 4–12 weeks post therapy. Transcriptomic analysis reveals that progenitor cells with higher expression of P63 gene have better therapeutic efficacy. These findings suggest that P63^+^ progenitor cells may offer a promising therapeutic approach for bronchiectasis. This study was registered at ClinicalTrials.gov(NCT03655808).

## Highlights

• Autologous P63^+^ progenitor cell transplantation is well tolerated
• Cell therapy improves lung function in patients with bronchiectasis
• Cell therapy shows reductions in lung damage and improvements in quality of life
• Higher P63 expression correlates with better therapeutic outcomes

## Graphical abstract

**Figure:**
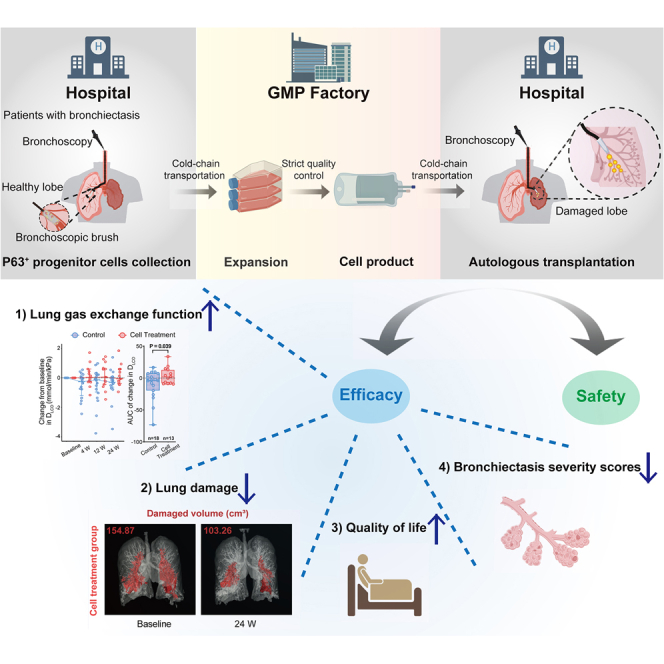

## Teaser

Yan et al. demonstrate that autologous P63^+^ progenitor cell transplantation enhances lung gas exchange function, quality of life, CT imaging outcomes, and bronchiectasis severity scores in patients with bronchiectasis. This study underscores the potential of regenerative cell therapy to promote lung repair, offering a promising strategy for the treatment of bronchiectasis.

## Introduction

Non-cystic fibrosis bronchiectasis (hereinafter referred to as “bronchiectasis”) is a severe chronic respiratory disease characterized by permanent dilation of the airways, recurrent infection, persistent pulmonary epithelium damage, and inflammation.^1^ The burden of bronchiectasis on patients is profound, with most suffering from daily symptoms of cough, sputum production, and intermittent exacerbations, ultimately leading to respiratory failure and diminished quality of life.^2^ The global prevalence of bronchiectasis is increasing,^3^^,^^4^ posing a significant health threat and economic burden to patients and society.^5^^,^^6^ Despite various management strategies, including antibiotics, mucoactive agents, and bronchodilators, licensed treatments are lacking, and clinical interventions remain palliative, with limited evidence supporting their efficacy in repairing damaged lung tissue or restoring pulmonary function.^7^^,^^8^^,^^9^^,^^10^ Consequently, there is an urgent need for regenerative therapies capable of repairing lung tissue damage and halting or reversing the progression of bronchiectasis.

The reparative processes of injured adult lung epithelium are mediated by the activation of various populations of lung-resident stem/progenitor cells, including P63^+^ KRT5^+^ basal progenitor cells located in the basal layer of the airway epithelium. However, the function of human P63^+^ progenitor cells in lung regeneration remains controversial.^11^^,^^12^^,^^13^^,^^14^ In recent decades, the function of human P63^+^ progenitor cells in the lung regeneration process has remained a controversial issue. While some studies suggest their potential to regenerate bronchial and alveolar epithelium,^12^^,^^13^^,^^14^^,^^15^^,^^16^ others indicate that P63^+^ basal progenitors may contribute to persistent pathology, such as bronchiolization or dysplastic tissue formation.^17^^,^^18^^,^^19^ Consequently, further investigation is warranted to determine the role of human P63^+^ progenitor cells in lung repair, particularly in the context of bronchiectasis.

Previous studies showed that human P63^+^ progenitor cells could be isolated from bronchoscopic brushed-off tissue from the patient’s bronchi^20^ and expanded in a feeder cell-based regenerative cloning culture (R-Clone) system. Transplantation of human P63^+^ progenitor cells into injured mouse lungs resulted in lung epithelial reconstitution and improved air exchange function.^21^^,^^22^^,^^23^ More pre-clinical data in rodents and non-human primates also demonstrated the safety and feasibility of intrapulmonary P63^+^ progenitor cell transplantation.^22^^,^^24^ In an early pilot clinical trial performed in two patients with bronchiectasis, both patients have shown promising outcomes following autologous P63^+^ progenitor cell transplantation, including significant improvements in pulmonary function and lung damage recovery.^21^ In a recent phase 1 trial, autologous P63^+^ progenitor cells were cloned and transplanted into patients with chronic obstructive pulmonary disease (COPD), resulting in statistically significant improvements in gas exchange function and walking distances.^24^ These previous works demonstrated the feasibility of large-scale *in vitro* expansion and encouraged us to study the therapeutic potential of autologous P63^+^ progenitor cells in patients with bronchiectasis.

## Results

### Cloning P63^+^ progenitor cells from patients with bronchiectasis

**Figure 1:**
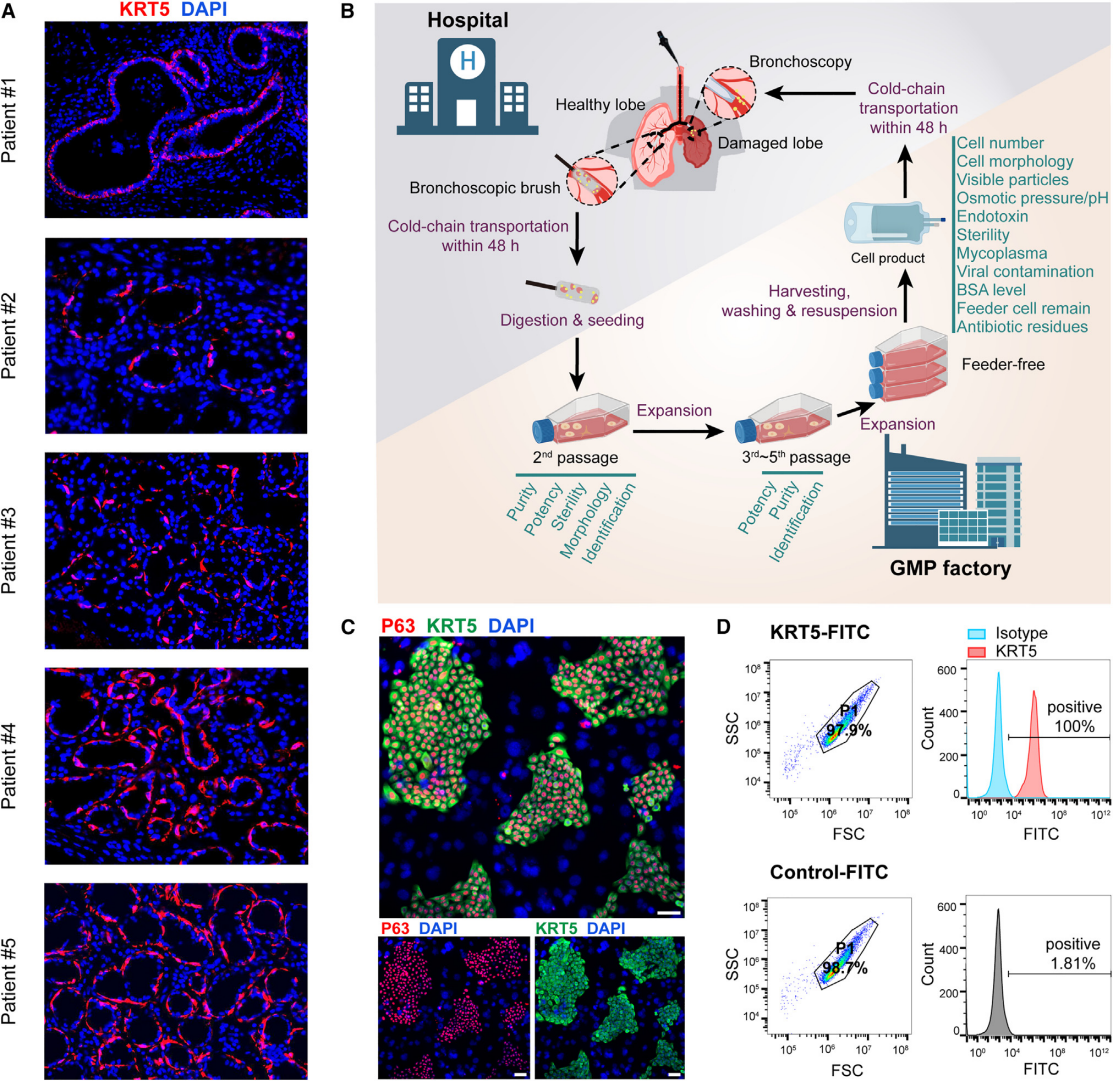
Characterization and cloning of P63^+^ progenitor cells from patients with bronchiectasis (A) Immunofluorescence staining showing KRT5^+^ (red) cells in lung sections from different patients with bronchiectasis (*n* = 5). Nuclei were counterstained with DAPI (blue). Scale bar, 50 μm. (B) A schematic diagram illustrating the manufacture, quality control, and clinical administration procedure of autologous P63^+^ progenitor cell products. (C) Cultured progenitor cell clones were immunostained with KRT5 and P63 markers. Scale bar, 100 μm. (D) Fluorescence-activated cell sorting (FACS) gating strategy for cell identity and purity test. KRT5 was immunostained as a marker of progenitor cells. SSC, side-scatter; FSC, forward scatter.

In healthy human lungs, the P63^+^ KRT5^+^ cells existed only in the airway epithelium. However, for those patients with severe lung diseases such as acute respiratory distress syndrome, idiopathic pulmonary fibrosis, and COPD, it is observed that the P63^+^ KRT5^+^ cells would appear in alveolar spaces, suggesting their possible participation of lung repair or regeneration process.^12^^,^^14^^,^^21^^,^^25^ However, for patients with bronchiectasis with recurrent bacterial infection, it remains unclear whether the P63^+^ progenitor cells mediated a similar process. In this study, we collected pulmonary tissues from 5 patients with bronchiectasis through surgical excision and performed immunostaining to examine the P63^+^ KRT5^+^ cell distribution in the lung. The result showed that in patient #1, all KRT5^+^ cells were lined in the airway epithelium and none of them were found in alveolar spaces. In patient #2, the KRT5^+^ cells were found in the alveolar spaces, forming a typical “bronchiolization” structure characterized by multi-layered cuboidal or columnar cells.^26^^,^^27^ Interestingly, in patient #3, #4, and #5, we found that some of the KRT5^+^ cells exhibited single-layered sphere morphology, forming air sac-like structures (Figure 1A). Interestingly, we noticed that all patient lungs except patient #1 were characterized by interstitial fibrosis. We also noticed that patient #3, #4, and #5 were all females with ≤2 years disease duration period and no bacterial growth in sputum culture, while patient #1 and #2 were males with ≥12 years disease duration period and detectable bacterial growth in sputum culture (Table S1). Immunostaining of consecutive pathological sections showed that the KRT5^+^ air sac-like areas also expressed type I alveolar cell gene AQP5 and endothelial cell gene CD31 (Figure S1A). Altogether, these observations suggested that the P63^+^ KRT5^+^ progenitor cells might have alveolar repair function in the lungs of some patients with bronchiectasis, probably in those patients with recent disease onset and no active infection in the lung.

In order to further study the repair function of P63^+^ progenitor cells in human, the cells were cloned from bronchiectasis patient airway and expanded similarly as previously reported.^24^ Briefly, the process involved collecting tiny brush sample tissues from the 3^rd^–5^th^ order bronchi of patients with bronchiectasis through bronchoscopy.^28^ For tissue collection, the healthiest lung lobe was chosen based on high-resolution computed tomography (HRCT) imaging, and the relatively healthy airway inner surface was selected under bronchoscopic imaging. The collected tissues were then digested by recombinant enzyme and grown on the regenerative cell clone (R-Clone) culture system, which selectively promotes the growth and expansion of progenitor cells. To prevent microbial contamination, gentamicin sulfate at a concentration of 200 μg/mL was applied at the primary passage, but not in the following 2–5 passages. The cell products were subjected to standard quality assays to assess items including cell number, cell morphology, visible particles, pH value, osmotic pressure, sterility, mycoplasma, endotoxin, viral contamination, bovine serum albumin (BSA) level, feeder cell remains, and antibiotic remains (Figure 1B). The expression of representative progenitor cell markers, KRT5 and P63, was confirmed through immunofluorescent staining of the cell colonies (Figure 1C). The cells at the last passage were analyzed by flow cytometry, which showed >99% cells were KRT5^+^/CD45^−^/CD105^−^/CD34^−^ (Figures 1D and S1B). The non-tumorigenic potential of cells was confirmed by soft-agar colony formation assay (Figure S1C).

At the last passage, cells were cultured in feeder-free condition until they reached 85%–100% confluency. The cells were harvested using xeno-free TrypLE and suspended in 30 mL saline as the final product, which was sealed in a cell preservation bag and shipped as fresh cells to Ruijin Hospital by cold-chain transport (2°C–8°C) within 48 h. For transplantation, the cell suspension was warmed to room temperature and evenly distributed into the 6 pulmonary segments with the most severe lesions according to computed tomography (CT) results, using bronchoscopy with 5 mL for each segment.

### Study population and baseline characteristics

In order to study whether the autologous P63^+^ progenitor cells could repair the bronchiectasis lung, we conducted a single-blind, randomized, controlled clinical trial (NCT03655808) between June 2020 to May 2023, to investigate the effect of P63^+^ progenitor cells on parenchyma repair in patients with bronchiectasis, which were diagnosed according to 2019 British Thoracic Society (BTS) guidelines.^29^ Only patients with the diffusing capacity of the lungs for carbon monoxide (D_LCO_) <80% of the predicted value were included in the study. Table S2 provides detailed patient inclusion and exclusion criteria.

Overall, we enrolled 37 patients with bronchiectasis in this study and randomly assigned them to the control or cell treatment group. Among them, 18 patients were assigned to the cell treatment group and 19 patients to the control group. Both the patients and the investigators, except for the bronchoscopy operators, remained masked to the group allocation for the duration of the study. Two patients, one in the cell treatment group and one in the control group, withdrew their previously written informed consent after randomization and did not receive treatment. Eventually, 18 and 17 patients were treated with bronchoscopic airway clearance treatment (B-ACT) or B-ACT plus autologous P63^+^ progenitor cells transplantation, respectively (Figure S2). Other standard-of-care treatments were continued as well in both groups. The demographic and clinical characteristics of patients in both groups at baseline showed no statistically significant difference (Table 1). However, it is noted that the patients in the cell treatment group had non-significant lower diffusion capacity as measured by D_LCO_ level (*p* value = 0.191) and non-significant higher Bronchiectasis Severity Index (BSI) (*p* value = 0.242) at baseline. For B-ACT, 120–200 mL saline was instilled into the patient’s lung followed by continuous suction to remove secretions in the respiratory tract. Patients in the cell treatment group were transplanted with 1–3 × 10^6^ autologous P63^+^ progenitor cells per kilogram body weight (Table S3) through bronchoscopy. Patients were followed up at 4, 12, and 24 weeks post cell transplantation for safety and efficacy outcome analysis.

**Table 1:** Baseline demographic and clinical characteristics

Demographics	Cell treatment group (*n* = 17)	Control group (*n* = 18)	Total (*n* = 35)	*p* value
Age (years)^a^	53.7 ± 13.5	50.4 ± 14.1	52.0 ± 13.7	0.499
Female gender^b^	9 (52.9%)	11 (61.1%)	20 (57.1%)	0.738
Body mass index (kg/m^2^)^a^	20.1 ± 3.5	19.8 ± 4.0	20.0 ± 3.7	0.832
Smokers^b^	3 (17.6%)	1 (5.6%)	4 (11.4%)	0.338

**Bronchiectasis characteristics**				

Duration of disease (years)^c^	16.0 (5.0, 31.0)	10.0 (6.5, 30.0)	12.0 (5.0, 30.0)	0.858
FEV1% predicted^a^	44.2 ± 18.3	49.1 ± 19.2	46.7 ± 18.7	0.442
D_LCO_% predicted^a^	54.5 ± 18.6	62.4 ± 16.1	58.8 ± 17.6	0.191
BSI score^a^	10.9 ± 3.9	9.3 ± 4.1	10.1 ± 4.0	0.242
SGRQ score^a^	52.9 ± 20.3	44.1 ± 21.8	48.4 ± 21.2	0.225
Exacerbations in the past year^c^	1.0 (1.0, 1.5)	1.0 (1.0, 2.0)	1.0 (1.0, 2.0)	0.386
Radiography involved lung lobes^c^	5.0 (4.0, 5.0)	4.0 (2.0, 5.0)	5.0 (3.0, 5.0)	0.207

**Etiology**				

Post TB infection^b^ Post non-TB infection^b^ Idiopathic^b^ Other^b^	2 (11.8%) 6 (35.3%) 8 (47.1%) 1 (5.9%)	2 (11.1%) 8 (44.4%) 7 (38.9%) 1 (5.6%)	4 (11.4%) 14 (40.0%) 15 (42.9%) 2 (5.7%)	0.942

**Comorbidities**				

COPD^b^	4 (23.5%)	4 (22.2%)	8 (22.9%)	>0.999
Asthma^b^	1 (5.9%)	0 (0)	1 (2.9%)	0.486
Chronic rhinitis or sinusitis^b^	6 (35.3%)	4 (22.2%)	10 (28.6%)	0.471

**Quality sputum culture**				

*Pseudomonas aeruginosa*^b^	6 (35.3%)	4 (22.2%)	10 (28.6%)	0.471
Other^b^	2 (11.8%)	1 (5.6%)	3 (8.6%)	0.603
No bacterial growth^b^	9 (52.9%)	13 (72.2%)	22 (62.9%)	0.305

**Medication for bronchiectasis**				

Oral antibiotics^b^	11 (64.7%)	9 (50.0%)	20 (57.1%)	0.500
Oral corticosteroid^b^	2 (11.8%)	0 (0)	2 (5.7%)	0.229
Inhaled corticosteroid^b^	3 (17.6%)	3 (16.7%)	6 (17.1%)	>0.999
Inhaled bronchodilator^b^	10 (58.8%)	12 (66.7%)	22 (62.9%)	0.733
Mucolytics^b^	11 (64.7%)	16 (88.9%)	27 (77.1%)	0.121

a Data were presented as mean ± standard deviation (SD).

b Data were presented as patient number (percentage of patients).

c Data were presented as median (interquartile range, IQR).

### Safety analysis

Adverse events occurred in 82.4% of patients in the cell treatment group and 83.3% of those in the control group (*p* value >0.999) (Table 2). The most common adverse events were fever (37.1%), hemoptysis (i.e., coughing up bloody sputum; 25.7%), and increased sputum (20.0%). Grade 1 adverse events occurred in 9 (52.9%) patients in the cell treatment group and 14 (77.8%) patients in the control group. Grade 2 adverse events occurred in 8 (47.1%) patients in the cell treatment group and 8 (44.4%) patients in the control group. Two grade 3 serious adverse events (SAEs) occurred in 2 patients in the cell treatment group: one was pneumothorax and the other one was acute exacerbation of COPD with type 2 respiratory failure. Both patients were hospitalized and recovered after standard treatment. Among all these adverse events, 25 out of 66 events (37.88%) were considered related to bronchoscopic surgery, with 17 grade 1 events, 7 grade 2 events, and 1 grade 3 event (pneumothorax) (Table S4). Other 41 recorded adverse events (62.12%) were considered unlikely to be related to bronchoscopic procedure or cell transplantation, with 23 grade 1 events, 17 grade 2 events, and 1 grade 3 event, as judged by the investigators (Table S5). No grade 4 or 5 adverse events were recorded. There was no relationship between cell doses and the frequency of adverse events (correlation coefficient = −0.14; *p* value = 0.590). Additionally, key laboratory indexes, including white blood cells, neutrophil to lymphocyte ratio, alanine aminotransferase, aspartate transaminase, creatinine, and creatine kinase, remained stable in both two groups after treatment (Figure S3). No death or tumor formation was reported in this trial and we are continuing life-long observation on the patients who received the cell therapy. Altogether, these data indicated that autologous P63^+^ progenitor cell transplantation therapy had an acceptable safety profile among patients with bronchiectasis.

**Table 2:** Incidence of adverse events

Events	Cell treatment group (*n* = 17)	Control group (*n* = 18)	Total (*n* = 35)	*p* value
Any adverse event^a^	14 (82.4%)	15 (83.3%)	29 (82.9%)	>0.999
Fever^a^	8 (47.1%)	5 (27.8%)	13 (37.1%)	0.305
Hemoptysis^a^^,^^b^	3 (17.6%)	6 (33.3%)	9 (25.7%)	0.443
Sputum increased^a^	4 (23.5%)	3 (16.7%)	7 (20.0%)	0.691
Cough increased^a^	3 (17.6%)	3 (16.7%)	6 (17.1%)	>0.999
Fatigue^a^	3 (17.6%)	3 (16.7%)	6 (17.1%)	>0.999
COVID-19^a^	2 (11.8%)	4 (22.2%)	6 (17.1%)	0.658
Bronchiectasis exacerbation^a^	2 (11.8%)	2 (11.1%)	4 (11.4%)	>0.999
Pharyngeal discomfort^a^	1 (5.9%)	3 (16.7%)	4 (11.4%)	0.603
Other^a^^,^^c^	4 (23.5%)	5 (27.8%)	9 (25.7%)	>0.999
Serious adverse events^a^^,^^d^	2 (11.8%)	0 (0)	2 (5.7%)	0.229

a Data were presented as patient number (percentage of patients).

b The term “hemoptysis” included bloody sputum in this study.

c Other adverse events included chest discomfort, dizziness, dyspnea, nausea, influenza, and anxiety.

d Serious adverse events occurred in 2 patients, one for pneumothorax and another for acute exacerbation of COPD.

### Primary efficacy outcomes

**Figure 2:**
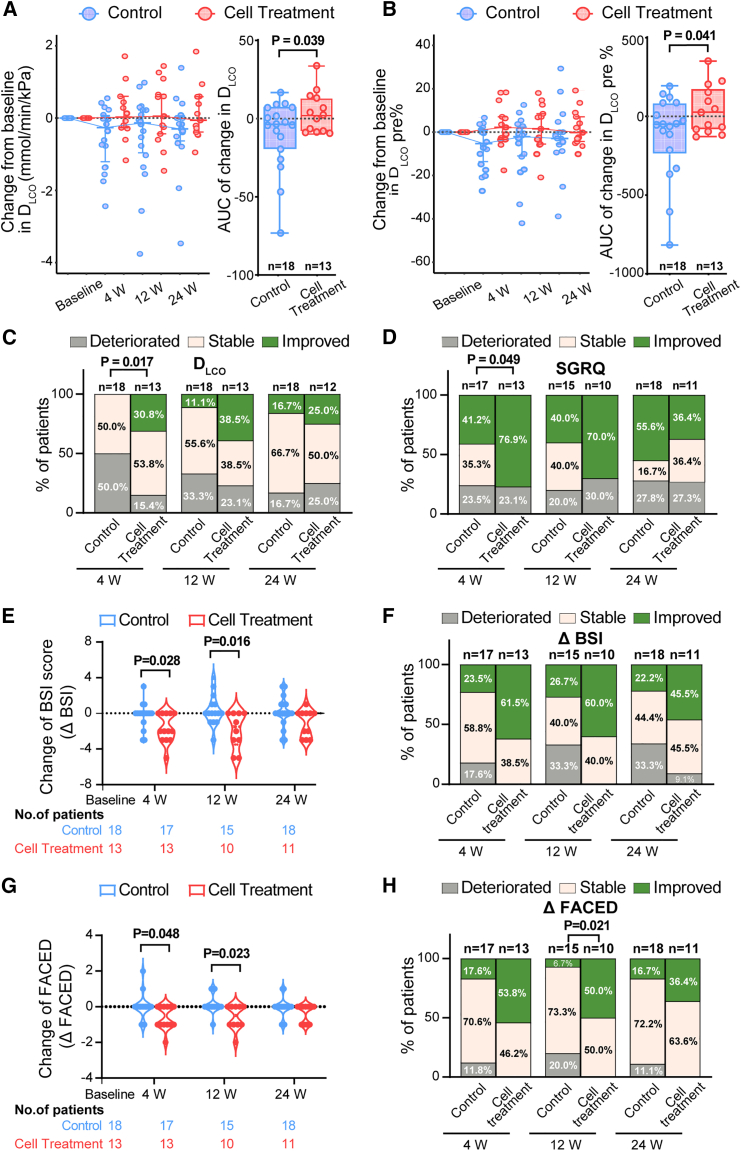
Changes of D_LCO_, SGRQ, BSI score, and FACED score at different time points after cell treatment (A) Left, changes of median D_LCO_ in both groups at week 4, 12, and 24. Data are represented as median (interquartile range, IQR). Right, boxplot showing the AUC of the D_LCO_ change from baseline to 24 weeks in both groups. Each dot indicated an individual patient. (B) Left, changes of median D_LCO_% of predicted in both groups at week 4, 12, and 24. Data are represented as median (IQR). Right, boxplot showing the AUC of the D_LCO_% change from baseline to 24 weeks in both groups. Each dot indicated an individual patient. (C) Column charts represent the proportion of patients who demonstrated >10% changes (improved) or < −10% changes (deteriorated) of D_LCO_ level at week 4, 12, and 24 after therapy. (D) Column charts represent the proportion of patients who had >4 units changes (deteriorated) or < −4 units changes (improved) of SGRQ score at week 4, 12, and 24 after cell treatment. (E) Violin plot showing changes in BSI score in both groups at week 4, 12, and 24. (F) Column charts represent the proportion of patients whose BSI score improved or deteriorated for ≥1 unit at week 4, 12, and 24 after cell treatment. (G) Violin plot showing changes in FACED score in both groups at week 4, 12, and 24. (H) Column charts represent the proportion of patients whose FACED score improved or deteriorated ≥1 unit at week 4, 12, and 24 after cell treatment.

The primary efficacy outcome of the current study is the change of D_LCO_ after cell treatment. D_LCO_ is a measurement of the gas transfer capacity of lung. Unlike the typically analyzed forced expiratory volume in 1 s (FEV_1_) parameter, which measures the airflow capacity, D_LCO_ represents the air exchange aspect of lung function that is quantitatively determined by the effective alveolar-capillary surface area. In chronic respiratory diseases including bronchiectasis, reduced gas transfer capacity was independently associated with higher mortality and lower quality of life.^30^^,^^31^^,^^32^^,^^33^ In this clinical study, we planned to use the change of D_LCO_ as the primary efficacy outcome to evaluate the therapeutic effect. The data showed that the median change from baseline of D_LCO_ level in the cell treatment group was better than the control group at all follow-up time points. We then calculated the area under the curve (AUC) of D_LCO_ to quantify the overall change of D_LCO_ from baseline from 4 weeks to 24 weeks. It was observed that the patients in the cell treatment group exhibited significantly larger cumulative change compared to the control group (mean ± standard deviation [SD], 4.06 ± 13.14 vs. −9.84 ± 22.46; 95% confidence interval [CI], 0.73 to 27.06; *p* value = 0.039) (Figure 2A).

Similarly, we also analyzed the D_LCO_% of predicted value. The data showed that the median change from baseline of D_LCO_% of predicted value in the cell treatment group was also better than the control group at all follow-up time points. We then calculated the AUC of D_LCO_% predicted to quantify the overall change of D_LCO_ from baseline from 4 to 24 weeks. It was observed that the patients in the cell treatment group exhibited significantly larger cumulative change compared to the control group in D_LCO_% predicted (mean ± SD, 43.47 ± 153.16 vs. −118.37 ± 264.64; 95% CI, 7.23 to 316.44; *p* value = 0.041) (Figure 2B).

For chronic lung diseases, the minimum clinically important difference for D_LCO_ was 10%–11% of baseline D_LCO_.^31^^,^^34^ Therefore, in our analysis, we also calculated the number of patients with >10% D_LCO_ change. There were 30.8% of patients in the cell treatment group who had more than a 10% increase of baseline D_LCO_% predicted at week 4, while none of the participants in the control group had >10% increase. There were only 15.4% of patients who had more than a 10% decrease of baseline D_LCO_% predicted at week 4 in the cell treatment group, as compared with 50.0% of patients in the control group. The difference between groups was statistically significant (*p* value = 0.017) (Figure 2C and Table S6). A similar tendency was also observed at week 12 and week 24, although the difference was not statistically significant. Further subgroup analysis indicated that compared to the control group, the improvement of D_LCO_ and D_LCO_% of predicted in the cell treatment group was consistent across most of the subgroups, except it was more pronounced in patients complicated with COPD (Figures S4 and S5). Altogether, the data indicated that in some of the patients with bronchiectasis, autologous P63^+^ progenitor cell transplantation could significantly improve the gas exchange capacity of lung.

### Secondary efficacy outcomes

One secondary efficacy outcome of the current study is the change of the St George’s Respiratory Questionnaire (SGRQ) score. SGRQ is used to assess the quality of life in patients with chronic respiratory diseases, and for the SGRQ score, a four-unit change has been proposed as clinically relevant.^35^^,^^36^ At week 4, we observed that the proportion of patients with an improvement exceeding four units was 76.9% in the cell treatment group and 41.2% in the control group, which demonstrated a statistically significant difference (*p* value = 0.049) (Figure 2D and Table S6). A similar tendency was also observed at week 12 but not week 24. We also used two different multidimensional grading systems to assess the severity of bronchiectasis before and after cell treatment: the BSI and FACED scores.^37^^,^^38^ Both scores could predict the exacerbation risk, hospitalization, and mortality of patients with bronchiectasis.^39^ The data showed that the cell treatment group demonstrated a significant decline in both BSI score and FACED score compared to the control group (Figures 2E–2H and S6–S8, Tables S7 and S8). Altogether, our results indicated that the autologous P63^+^ progenitor cell transplantation could improve quality of life and decrease the severity of bronchiectasis during 4–12 weeks post cell transplantation, while at 24 weeks post cell transplantation, the beneficial effect was no longer obvious.

**Figure 3:**
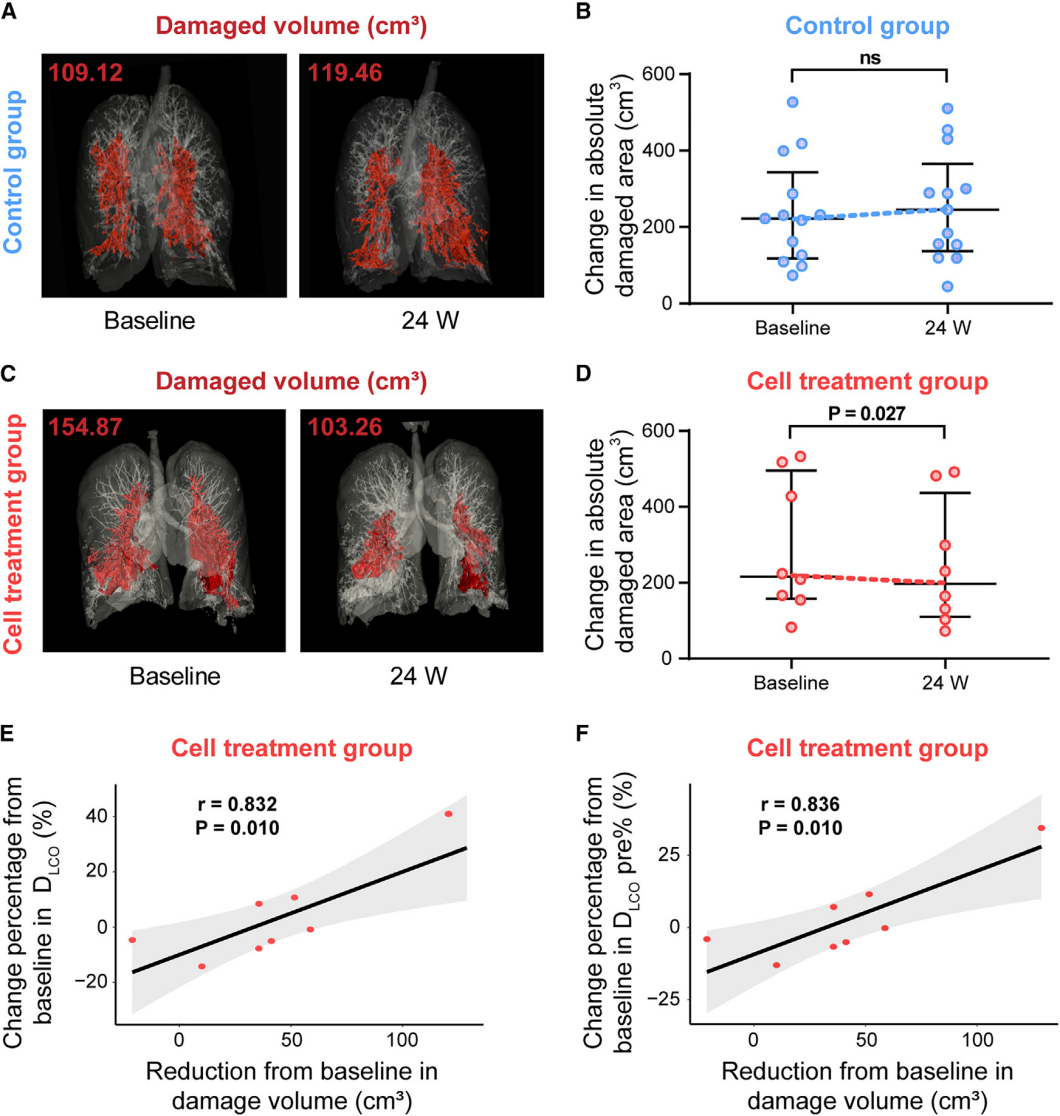
Changes in CT damaged areas at week 24 after cell treatment (A) The lung of patient #9003 in the control group was scanned by HRCT and 3D visualized. The red zone indicated the lung damaged area. (B) Changes in the absolute damaged area using HRCT 3D visualization and quantification analysis in the control group. Each dot represented an individual patient. Data are represented as mean ± standard error of the mean (SEM). The paired Student’s t test was performed. (C) The lung of patient #9018 in the cell treatment group was scanned by HRCT and 3D visualized. The red zone indicated the lung damaged area. (D) Changes in the absolute damaged area using HRCT 3D visualization and quantification analysis in the cell treatment group. Each dot represented an individual patient. Data are represented as mean ± SEM. The paired Student’s t test was performed. (E) Pearson correlations between the change from baseline in the damaged volume and the D_LCO_ in the cell treatment group. Each dot represented an individual patient. (F) Pearson correlations between the change from baseline in the damaged volume and the D_LCO_% of predicted in the cell treatment group. Each dot represented an individual patient. Reduction from baseline = −1 × (change from baseline).

We also analyzed the HRCT data of patients before and after cell treatment. As the morphology of bronchiectasis and mucus plugs assessed by experts in a blind manner showed no obvious difference between the two groups, we used computational image processing software for in-depth analysis. Three-dimensional (3D) visualization of consecutive CT images by 3D Slicer could measure the damaged area, including bronchial dilation and inflammatory lesions. Figure 3A showed a representative 3D lung visualization image of patient #9003 from the control group, illustrating the increase in lung damage area (Figure 3B). In contrast, Figure 3C showed a representative 3D lung visualization image of patient #9018 from the cell therapy group, demonstrating that the lung damage area was largely decreased following P63^+^ progenitor cell transplantation (Figure 3D). Comparing to the control group, the patients in the cell treatment group demonstrated a statistically significant decrease in the damaged lung area over the course of 24 weeks (Figures 3B and 3D). Furthermore, we observed a significant association between the change from baseline to 24 weeks in the damaged volume and the D_LCO_ in the cell treatment group (correlation coefficient = 0.832; *p* value = 0.010) (Figures 3E and S9A). Similarly, Pearson correlation analysis illustrated that the improvement of the damaged volume was also associated with the D_LCO_% of predicted in the cell treatment group (correlation coefficient = 0.836; *p* value = 0.010) (Figures 3F and S9B). These results were consistent with our findings that autologous P63^+^ progenitor cells transplantation could improve the gas exchange capacity in patients with bronchiectasis.

Furthermore, we observed significant improvement in alveolar ventilation and total lung capacity exclusively at week 4 (Table S7). In addition, we also observed that the increase of inspiratory capacity was larger in the cell treatment group than in the control group exclusively at week 24 (Table S7). Other efficacy outcomes including FEV_1_, forced vital capacity (FVC), FEV_1_/FVC, maximum mid expiratory flow, maximum voluntary ventilation, 6-min walking distance, and distance-saturation product were similar between two groups throughout the 24-week period after treatment (Table S7 and Figure S10).

### Transcriptomic analysis of patient cells

**Figure 4:**
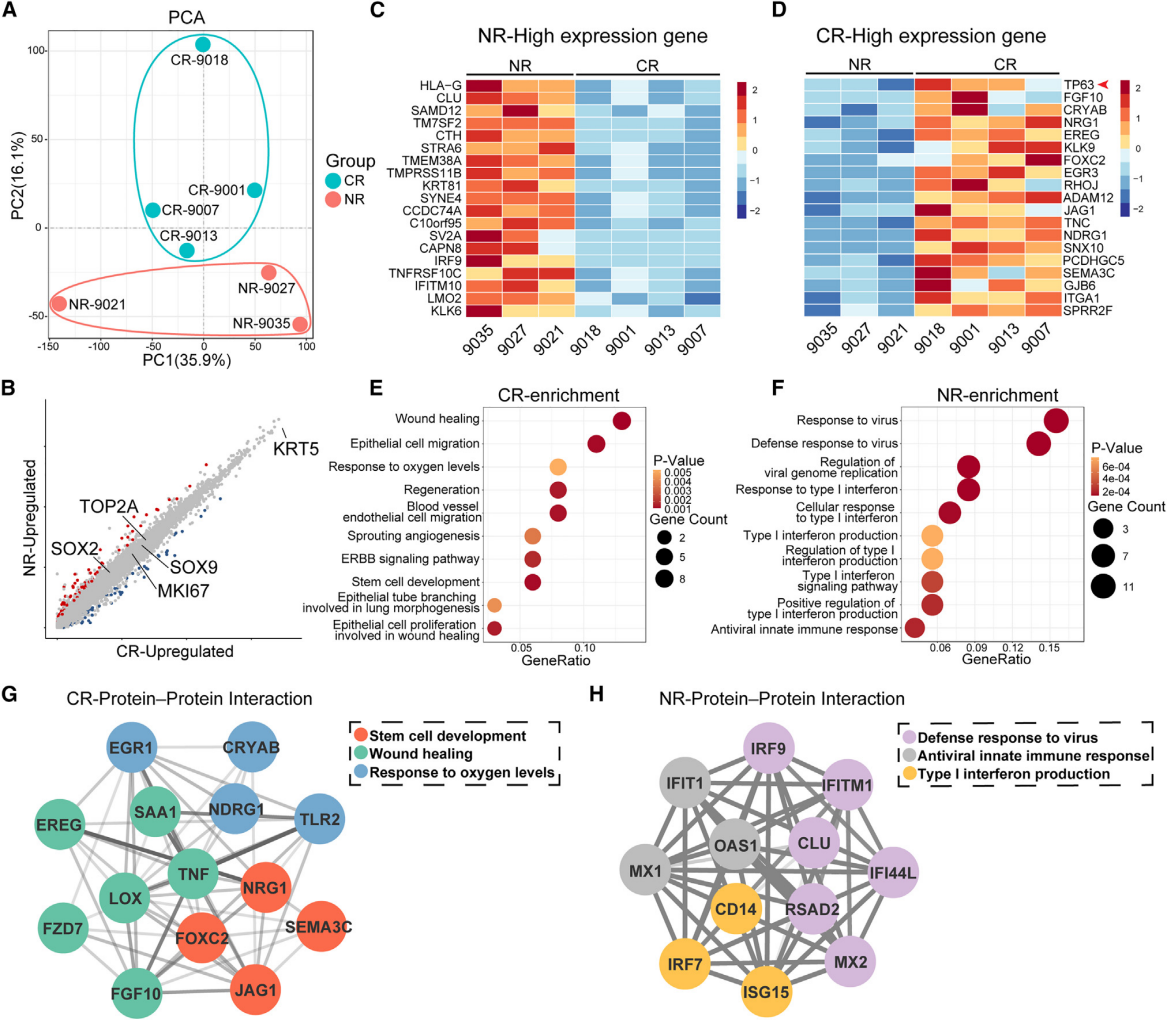
Transcriptomic analysis of progenitor cells derived from different patients (A) Unsupervised principal component analysis of RNA-seq data progenitor cells derived from complete responsive (CR) and non-responsive (NR) patients (CR, *n* = 4; NR, *n* = 3). (B) Scatterplot of gene expression of NR and CR cell lines. Gray dots represented genes showing no significantly different expression levels. (C and D) Expression heatmap of gene sets differentially expressed in NR (C) and CR (D) progenitor cells (CR, *n* = 4; NR, *n* = 3). (E and F) GO terms that were significantly enriched in the CR (E) and NR (F) groups (*p* value <0.05) (CR, *n* = 4; NR, *n* = 3). (G and H) Protein interaction network analysis of the expression of proteins associated with specific GO terms and their interaction relationship in CR (G) and NR (H) groups (CR, *n* = 4; NR, *n* = 3).

We were particularly interested in understanding why some patients respond to the cell therapy better than others and hoping to identify factors that may determine treatment outcomes. Pearson correlation analysis illustrated that the improvement of the D_LCO_ level over the 24-week period was not associated with the dose of cells transplanted to patients in the cell treatment group (correlation coefficient = −0.18; *p* value = 0.616). Then we asked whether the difference in gene expression profiles of patients’ progenitor cells might contribute to the difference in treatment efficacy. Therefore, we analyzed P63^+^ progenitor cells isolated from 7 patients in the cell treatment group. Among them, 4 patients demonstrated a “complete response” to autologous cell transplant therapy, defined as patients with >10% D_LCO_ change from baseline level as well as improvement of SGRQ and mMRC (complete responsive [CR]-patient #9001, #9007, #9013, and #9018). The other 3 patients demonstrated “no response” to treatment (non-responsive [NR]-patient #9021, #9027, and #9035), whose D_LCO_ change is within ±10% of baseline level. We performed whole-genome RNA sequencing to analyze the transcriptome of progenitor cells from these 7 patients. Unsupervised principal component analysis of the whole-transcriptome data showed that four cell lines from CR patients showed a tendency to separate from three cell lines from NR patients (Figure 4A). These data suggested that the differences in overall gene expression profiles might be related to different treatment outcomes, as CR and NR patients were similar in terms of demographics, disease severity, comorbidities, lung function, and medication at baseline (Table S9).

Further studies showed that both groups expressed similar levels of the progenitor genes KRT5, SOX9, and SOX2 and cell proliferation markers KI67 and TOP2A (Figure 4B). However, progenitor cells from NR patients highly expressed 78 genes, and many of their functions were related to inflammation and virus, such as HLA-G and IRF9 (Figure 4C).^40^ In contrast, progenitor cells from CR patients maintained higher expression level of the key transcriptional factor P63. Additionally, CR cells highly expressed other 67 genes, and many of their functions were related to lung development, such as FGF10, JAG1, and EREG (Figure 4D).^41^^,^^42^^,^^43^ Gene ontology (GO) analysis indicated that the CR cells were enriched in genes related to wound healing, regeneration, and lung morphogenesis (Figures 4E and S11A). In contrast, GO analysis showed that NR cells highly expressed genes related to virus and interferon responses (Figures 4F and S11B). Protein-protein interaction network analysis showed that in CR patients, the proteins related to stem cell pathways frequently interacted with proteins involved in wound healing and responding to oxygen levels (Figure 4G). In contrast, in NR cells, proteins involved in type I interferon response interacted with proteins involved in innate immunity and virus resistance at high frequencies (Figure 4H), and we speculated that the NR cells might have been modified in patient lungs to fulfill the pathogen clearance function, instead of the normal tissue repair function. Overall, these data suggested that the P63-high normal progenitor cells might have a better therapeutic effect than the P63-low variant progenitor cells. These results were consistent with previous findings in patients with COPD^24^ and indicated that the treatment of bronchiectasis needed to move toward an endophenotypic precision medicine approach.^44^

## Discussion

The clinical course of non-CF bronchiectasis is characterized by intermittent exacerbations and irreversible deterioration, which may progress to respiratory failure and even mortality. Current standard treatments, including antibiotics, mucoactive agents, bronchodilators, corticosteroids, and airway clearance therapy, offer only symptomatic relief and do not address the underlying structural lung damage. Thus, there is an urgent need for stem/progenitor cell treatment options aimed at lung regeneration. Our previous pilot clinical trial demonstrated the potential of autologous P63^+^ progenitor cell transplantation to improve pulmonary function in two patients with bronchiectasis. Consecutive CT revealed regional bronchiectasis recovery in one of these patients.^21^^,^^45^ Building upon this concept, the current randomized, controlled, single-blind clinical study aimed to investigate the safety and efficacy of intrapulmonary transplantation of P63^+^ progenitor cells in a larger cohort.

The current study demonstrated the feasibility of isolating, expanding, and transplanting P63^+^ progenitor cells in patients with bronchiectasis. The incidences of adverse events were similar between the two groups, with most events attributed to the bronchoscopy procedure or natural progression of bronchiectasis. SAEs occurred in 2 patients in the cell treatment group. One patient experienced a pneumothorax immediately after bronchoscopic surgery to collect P63^+^ progenitor cells. Given the compromised nature of bronchial walls in patients with bronchiectasis,^46^ this pneumothorax was likely due to the brush’s manipulation during bronchoscopy. Therefore, future studies should ensure gentle bronchoscopic procedures performed by well-trained physicians. Another SAE involved a patient who developed a common cold and subsequently experienced an acute exacerbation of COPD 8 weeks after cell transplantation. This patient previously experienced 3–4 times of COPD acute exacerbations per year, and the cause of the exacerbation event this time was clear. Additionally, the occurrence time is far from transplantation surgery. Thus, both of the two SAEs were considered unrelated to cell transplantation therapy. These two patients recovered well following standard conservative treatment in the hospital. Importantly, among all patients, no tumor formation was observed during the entire follow-up period, indicating the overall safety of autologous P63^+^ progenitor cell transplantation in patients with bronchiectasis.

In addition to safety evaluation, the data also revealed significant improvements in pulmonary gas transfer function (D_LCO_), quality of life, CT images, and bronchiectasis severity scores following cell transplantation, suggesting potential therapeutic benefits. Currently, pharmacotherapy for bronchiectasis primarily focuses on infection control using antibiotics and alleviating airflow restriction with short- or long-acting bronchodilators. However, these approaches fail to halt or reverse bronchiolar and alveolar destruction and do not positively impact gas transfer parameters (D_LCO_). Previous studies have reported impaired D_LCO_ in 55.7% of patients with bronchiectasis, with a progressive decline of 2.9% per year.^44^^,^^47^ D_LCO_ values below 85% of predicted values are significant predictors of all-cause mortality, even in the absence of apparent clinical respiratory disease.^48^ In chronic respiratory conditions such as bronchiectasis and COPD, declining D_LCO_ is associated with higher mortality and lower quality of life,^30^^,^^31^^,^^32^^,^^33^ independent of airflow obstruction severity and other clinical variables. In our study, a significant improvement in gas transfer function was observed within 12 weeks post therapy. The time-limited benefit of cell transplantation could be due to that in the highly infectious microenvironment of bronchiectasis lungs, the transplanted cells cannot persistently engraft, in contrast to our previous finding of persistent beneficial effect in patients with COPD.^24^ Consistently, CT imaging indicated partial lung injury repair in patients who underwent cell transplantation. Additionally, we observed improvements in BSI and FACED scores following cell treatment. These scoring systems are commonly used to assess the severity and prognosis of bronchiectasis and demonstrate high predictive power.^39^

The mechanism underlying P63^+^ progenitor cell therapy’s improvement of D_LCO_ and other health status in patients with bronchiectasis requires further investigation. As mentioned in the introduction, the exact function of P63^+^ progenitor cells in the lungs of patients with various pulmonary diseases remains unclear. While these cells have demonstrated significant bronchiolar and alveolar repair potential, aberrant P63^+^ basaloid cells have been found in the alveolar space of fibrotic lungs,^18^ and P63^+^ basal cell hyperplasia has been associated with persistent airway remodeling in COPD.^49^^,^^50^ Regarding bronchiectasis, previous studies have indicated the expansion of P63^+^ KRT5^+^ lung basal progenitor cells in dilated bronchioles.^51^ Additionally, we observed that these cells could form air sac-like structures in the alveolar spaces of some patients with bronchiectasis within two years of onset. This suggests that endogenous P63^+^ cells may possess lung repair functions under certain conditions before becoming exhausted as the disease progresses. These repair processes likely involve multiple mechanisms, including the regeneration of damaged airway epithelium, re-epithelialization of injured alveolar spaces, and paracrine signaling of lung-repairing growth factors or anti-bacterial peptides. Interestingly, we found that the D_LCO_ improvement was associated with the higher expression level of P63 in progenitor cells in some patient samples. A similar observation was described in another phase 1 study using autologous P63^+^ progenitor cells to treat COPD.^24^ However, further investigations in animal models and human subjects are needed to fully elucidate the complex mechanisms underlying these observations. In the future, based on a better understanding of the repair mechanism, appropriate methods could be developed to select P63^high^ cells for therapeutic purposes, or measures could be implemented to reprogram cells for improved therapeutic effects. Further research in this area holds the potential to enhance the efficacy of P63^+^ progenitor cell therapy for bronchiectasis and other respiratory conditions.

### Limitations of the study

The current work has several limitations that warrant attention in future research. Firstly, considering the diverse etiologies of bronchiectasis, the relatively small number of participants and the single-center nature of the study may limit the generalizability of the findings, especially the beneficial effect in patients without severe deficiency of gas exchange function. Therefore, additional verification in larger, multicenter cohorts is necessary to establish the safety and clinical efficacy of P63^+^ progenitor cell transplantation. Secondly, the trial was not specifically designed to elucidate the etiology of enrolled patients, leading to potential variability in responses to cell therapy due to the heterogeneous nature of non-CF bronchiectasis. Additionally, the genetic and epigenetic background of progenitor cells cloned from individual patients may contribute to distinct responses to therapy. Future studies should consider stratifying patients based on etiological factors and explore the impact of genetic and epigenetic variations on treatment outcomes. Thirdly, the 24-week follow-up duration may be insufficient to assess long-term safety and efficacy, particularly regarding exacerbation frequency and overall mortality. Longer-term follow-up periods are necessary to evaluate the durability of treatment effects and potential late-onset adverse events. Finally, we noted a higher drop-out rate in the cell treatment group. This was mainly due to an uneven geographic distribution of patients after randomization. A larger proportion of patients in the control group (47.06% vs. 27.78%) resided in the Yangtze River Delta (YRD) region of China, which is closer to the Shanghai Ruijin Hospital where the study took place. Due to the stringent COVID-19 lockdown policy enforced in Shanghai in 2022,^52^ several patients in the cell treatment group who resided outside of the YRD region were lost to follow-up. We hope that future studies will be able to address the limitations of the current study and provide more reliable evidence regarding the safety and efficacy of the treatment.

## Resource availability

### Lead contact

Further information and requests for resources and reagents should be directed to and will be fulfilled by the lead contact, Jieming Qu (jmqu0906@163.com).

### Materials availability

This study did not generate new unique reagents.

### Data and code availability

RNA-seq data generated during this study have been deposited at GEO and the accession number is listed in the key resources table.

Qualified researchers may request access to individual patient-level clinical data for eligible studies. However, due to proprietary considerations, the datasets generated and/or analyzed during the current study are not publicly available. All data will be shared in aggregate form and can be requested once the article has been published, if there is not a reasonable likelihood of participant reidentification. To request access to patient-level data, please contact the lead contact, who will decide whether or not to provide the data.

No custom code was generated.

Any additional information required to reanalyze the data reported in this work paper is available from the lead contact upon request.

## Acknowledgments

This study was supported by Shanghai Shenkang Hospital Development Center Clinical Science and Technology Innovation Project (SHDC12018102 to J.Q.), Shanghai Municipal Key Clinical Specialty (shslczdzk02202 to J.Q.), Shanghai Top-Priority Clinical Key Disciplines Construction Project (2017ZZ02014 to J.Q.), Shanghai Key Laboratory of Emergency Prevention, Diagnosis and Treatment of Respiratory Infectious Diseases (20dz2261100 to J.Q.), Cultivation Project of Shanghai Major Infectious Disease Research Base (20dz2210500 to J.Q.), 10.13039/100020732Innovative Research Team of High-level Local Universities in Shanghai (to J.Q.), Shanghai Municipal Hospital Respiratory and Critical Care Medicine Specialist Alliance (to J.Q.), Shanghai Sailing Program (21YF1427000 to M.S.), 10.13039/100014717National Science Fund for Excellent Young Scholars (82122038to W. Zuo), Shanghai Science and Technology Talents Program (19QB1403100 to W. Zuo), Shanghai Municipal Science and Technology Small and Medium-sized Enterprise Technology Innovation Fund Program (230H1007900 to W. Zuo), National Biopharmaceutical Technology Innovation Center Cell Therapy 'Open Bidding and Taking the Lead' Technical Research Project (NCTIB2023XB01011 to W. Zuo), and Jiangsu Province Science and Technology Special Funds (Key Research and Development Plan for Social Development) Project (BE2023727 to W. Zuo). The clinical trial part was also partially funded by Regend Therapeutics, Ltd..

## Author contributions

J.Q. and W. Zuo were responsible for the study design and coordination of all study-related activities and contributed to the evaluation and interpretation of study data and manuscript writing. M.Z., Y.F., L.Z., Y.G., T.Y., and Y.H. performed the bronchoscopy. M.Z. and Y.F. generated the random allocation sequence. C.D., Q.Z., X.W., J.Z., R.D., L. Ni, and Z.B. recruited the patients and assigned participants to groups. J.Y., W. Zhang, X.L., L. Niu, M.S., P.W., and T.Z. collected and assembled the data. J.Y. and W. Zhang did the statistical analysis. All authors were involved in data interpretation and the writing, revision, and critical review of the manuscript. All authors had full access to all the data in the study and had final responsibility for the decision to submit for publication. J.Q., W. Zuo, and M.Z. accessed and verified the underlying data.

## Declaration of interests

W. Zuo and T.Z. have an equity interest in Regend Therapeutics, holding the patent for human lung progenitor cell isolation and expansion technique.

## STAR★Methods

### Key resources table

**Table:** 

REAGENT or RESOURCE	SOURCE	IDENTIFIER
**Antibodies**		

Rabbit Cytokeratin 5 Monoclonal Antibody	Thermo Fisher	Cat#MA5-14473; RRID: AB_10979451
Rabbit CD31 Polyclonal Antibody	Proteintech	Cat#28083-1-AP; RRID: AB_2881055
Rabbit Recombinant Anti-Aquaporin 5 antibody	Abcam	Cat#ab92320; RRID: AB_2049171
Mouse Anti-p63 antibody	Abcam	Cat#ab735; RRID: AB_305870
Rabbit Recombinant Anti-Cytokeratin 5 antibody	Abcam	Cat#ab52635; RRID: AB_869890
Alexa Fluor-conjugated Donkey 488	Thermo Fisher	Cat#A21206; RRID: AB_2535792
PE Mouse Anti-Human CD45 Antibody	BD Pharmingen	Cat#560975; RRID: AB_560975
PE Mouse Anti-Human CD34 Antibody	BD Pharmingen	Cat#560941; RRID: AB_10522562
FITC Mouse Anti-Human CD105	BD Pharmingen	Cat#561443; RRID: AB_10714629
FITC Mouse Anti-Human IgG Antibody	BD Pharmingen	Cat#560952; RRID: AB_2648727
PE Mouse IgG1 κ Isotype Ctrl Antibody	BD Pharmingen	Cat#557646; RRID: AB_10519360
Alexa-conjugated Donkey 594 secondary antibodies	Life Technologies	Cat#A-21207; RRID: AB_141637
Alexa-conjugated Donkey 488 secondary antibodies	Life Technologies	Cat#A-21206; RRID: AB_2535792

**Chemicals, peptides, and recombinant proteins**		

Citrate Buffer (pH = 6)	Sigma-Aldrich	Cat#c9999
DAPI	Roche	Cat#10236276001
PBS	Multicell	Cat#311-425-CL
Vectashield	Vector Labs	Cat#H-1000-10
TRIzol Reagent	Invitrogen	Cat#15596026CN

**Deposited data**		

RNA-Seq data	This paper	GEO: GSE261102

**Software and algorithms**		

Statistical Package for the Social Science (version 25.0)	IBM Corp	https://www.ibm.com/cn-zh/products/spss-statistics
GraphPad Prism (version 9.0)	GraphPad Software, Inc.	https://www.graphpad.com
R package using ggplot2 (version 3.4.2)	R Project	https://cran.r-project.org/web/packages/ggplot2/index.html
R package using DESeq2 (version 1.38.3)	R Project	https://bioconductor.org/packages/release/bioc/html/DESeq2.html
R package using ClusterProfiler (version 4.6.2)	R Project	https://bioconductor.org/packages/release/bioc/html/clusterProfiler.html
R (version 4.2.3)	R Project	https://www.r-project.org
Cytoscape (version3.10.0)	Cytoscape Consortium	https://cytoscape.org
R package using pheatmap (version 1.0.12)	R Project	https://cran.r-project.org/web/packages/pheatmap/index.html
3D Slicer (version 5.2.2)	Fedorov et al., 2012	https://www.slicer.org
FlowJo	N/A	https://www.flowjo.com

### Experimental model and study participant details

#### Trial design

A randomized, single-blind, controlled trial was conducted at Ruijin Hospital, Shanghai Jiao Tong University School of Medicine. Patients enrollment began on June 21, 2020, and the last patient follow-up visit was on May 17, 2023. All the eligible patients met the criteria by the day of enrollment. Written informed consent was obtained after discussion with the patient or an appropriate surrogate. This clinical trial was approved by the Ethics Commission of Ruijin Hospital (2018-10-5) and registered with ClinicalTrials.gov, number NCT03655808. Detailed clinical trial protocol was shown in Data S1. The cell dose range (1–3 × 10^6^ kg/body weight) was chosen based on previous studies.^21^^,^^24^ Within the given range, for each patient, the exact cell dose was determined by the cell number harvested at last.

#### Participants

Patients aged 18 to 75 years, with a diagnosis of bronchiectasis confirmed by chest HRCT and remaining clinically stable for at least 2 weeks, were recruited from the outpatient clinics of Ruijin Hospital. In addition, all enrolled patients had a D_LCO_ of less than 80% of the predicted value, were suitable for bronchoscopy, and were willing to receive autologous P63^+^ progenitor cells transplantation therapy. The key exclusion criteria included active pulmonary tuberculosis, uncontrolled asthma, extremely severe COPD, respiratory failure, major hepatic or renal dysfunction, pregnancy or breast-feeding. A complete list of inclusion and exclusion criteria was shown in Table S2.

#### Randomization and blinding

As the number of participants was relatively small and the trial was single center, eligibility patients were assigned according to a random number table, with sequentially numbered in a 1:1 ratio generated by computer, to receive either B-ACT plus autologous P63^+^ progenitor cells transplantation (cell treatment group) or B-ACT therapy only (control group). The opaque sealed envelope method was used to conceal the allocation sequence. Both patients and investigators, except for the bronchoscopy operators, remained masked to the treatment assignment for the duration of the study. That is, only the investigators who performed the bronchoscopy were unblinded. The non-blinded investigators should not disclose any blind information to other investigators, participants, or clinic staff.

#### Interventions

Firstly, a comprehensive assessment of patients was conducted to check whether the patients were able to tolerate the bronchoscopy. Mainly through the following examinations: blood test, infectious diseases related indicators detections, electrocardiogram, chest HRCT, pulmonary function examination, and arterial blood gas analysis if necessary. Preoperative analysis and discussion were conducted according to the requirement of bronchoscopy. Patients and their families were fully informed before the bronchoscope, and the informed consents were signed. The bronchoscopy was performed by board-certified respiratory physicians of Ruijin Hospital using a flexible fiberoptic bronchoscope. Before the bronchoscopy, oropharyngeal and laryngeal anesthesia was obtained by administration of 2 mL of nebulized 4% lidocaine, followed by 1 mL of 2% topical lidocaine sprayed into the patient’s oral and nasal cavities. Operators checked whether the patients had active denture and removed it in time to prevent aspiration. Oxygen was given to one side of the nasal tract and oxygen saturation and pulse were monitored. For patients in the control group, they were only given B-ACT therapy. B-ACT therapy was performed on all patients in both groups according to the protocol described in our previous study.^53^ In brief, continuous suction was performed with the sputum aspirator from the trachea to the subsegmental bronchi during the entering of the bronchoscope to remove the visible secretions from the entire respiratory tract, and then operators used 120–200 mL normal saline to collect lavage fluid (the volume various depending on the operator’s judgment). For patients in the cell treatment group, on the basis of B-ACT therapy, airway tissues were collected from patients in the cell treatment group by a disposable 2-mm brush. Operators gently glided the brush back and forth 1 or 2 times in 3∼5^th^ order bronchi within the relatively healthy area of the lung, which showed no obvious sign of lesions based on chest HRCT scans and bronchoscopic scope.

The obtained tissues were washed and enzymatically digested to form a single-cell suspension, which was then cultured under the R-Clone system, a patented technique of Regend Therapeutics, Ltd. Before releasing for therapeutic purpose, the expanded P63^+^ progenitor cells underwent a series of tests, including cell identity, cell purity, viable cell count, viability, sterility, mycoplasma detection, biological efficacy, endotoxin, viral contamination, BSA remain content, antibiotic remain content, tumorigenicity pH, osmolality, product appearance, and visible particles.

The P63^+^ progenitor cells product contained 1 to 3 × 10^6^ cells/kg body weight suspended in 30 mL sterile 0.9% normal saline and was shipped to Ruijin Hospital in an ice box with a real-time monitoring and alarm device for temperature and location to ensure the required storage conditions (2–8°C). Shipping of cell products by car from the Regend cell factory to the hospital generally took less than 3 h. Upon receipt, the cellular product was inspected and 0.5 mL was kept as the retained sample in a liquid nitrogen storage tank in Ruijin Hospital. The rest was immediately sent to the bronchoscope room.

Patients underwent examinations 1–3 days before the second bronchoscopy and physicians assessed the patients again to determine whether they were suitable for bronchoscopy. For patients in the control group, they were given B-ACT therapy again. And for patients in the cell treatment group, after B-ACT therapy, they were also given autologous P63^+^ progenitor cells transplantation. Cell suspension was pre-warmed to approximately 37°C 15 min before use and kept in a syringe for later use.

During the cell transplantation process, all the patients received standard monitoring systems, including electrocardiogram, heart rate, non-invasive arterial blood pressure, and peripheral oxygen saturation (SpO_2_) in the operating room. The patients were asked to open the mouth as wide as possible and then the oral cavity and hypopharynx mucosa were sprayed with 2% lidocaine 3 times within 20 min before the procedure. A bite block was placed between the teeth of patients, and the operator advanced the fiberoptic bronchoscope downward along the oropharyngeal curve until the epiglottis and glottis were visible. The fiberoptic bronchoscope was inserted into the trachea after the front of the bronchoscope had passed through the vocal cords.

Six lung segments with the most severe lesions were selected by the team of doctors before bronchoscopy according to CT results. After the bronchoalveolar lavage was completed, the lavage fluid in the affected area was required to aspirate as much as possible. When the oxygen saturation of patients maintained above 92%, 5 mL of the cell suspension was slowly and gently pushed into each lung segment via the working channel of the bronchoscope with a 20 mL syringe in around 30 s, and the severely damaged lung segment could be injected more than once.

After bronchoscopy, patients were told to fast, avoid coughing violently, and keep a supine position without pillow for at least 2 h. Physicians closely monitored the physical signs of patients including body temperature, pulse, respiration, blood pressure, oxygen saturation, and any signs of adverse reaction. Patients was discharged from the hospital 2–3 days after bronchoscopy.

#### Outcomes

Patients were followed up by clinical physicians at Week 4, Week 12, and Week 24 in Ruijin Hospital after the second bronchoscopy operation. In order to evaluate the safety and tolerability of autologous P63^+^ progenitor cells transplantation, we monitored adverse events from enrollment through 24 weeks after treatment. Meanwhile, we recorded the clinical information and laboratory tests of all the participants on baseline, and Week 4, 12, and 24. The data included the following: (1) demographic data, principal symptoms, medical history related to bronchiectasis, comorbidity, and medicine treatment; (2) vital signs and physical examination results; (3) laboratory tests, including blood and urine routine examinations, chemistry panels assessing liver and kidney function, lactate dehydrogenase (LDH), CK, blood glucose, and arterial blood gas analysis; (4) electrocardiogram results.

The primary efficacy endpoints were the changes from baseline in D_LCO_ after treatment. Efficacy was also evaluated with respect to the following secondary endpoint measures: the changes from baseline in other pulmonary function parameters including FEV_1_, FVC, FEV_1_/FVC, MMEF, and MVV, at Week 4, 12, and 24; the changes from baseline in 6MWD and DSP at Week 4, 12, and 24; the changes from baseline in SGRQ at Week 4, 12, and 24; the changes from baseline in BSI and FACED scores at Week 4, 12, and 24. These endpoints were compared between the cell treatment group and the control group. Data collections were performed according to standardized protocol by clinical physicians involved in this research.

Serial pulmonary function tests were all performed according to the American Thoracic Society (ATS) and European Respiratory Society (ERS) guidelines.^54^^,^^55^^,^^56^^,^^57^^,^^58^^,^^59^ This included measurement of the flow-volume curve and spirometry, lung volume by single breath dilution and plethysmography, airway resistance during panting at functional residual capacity (FRC), and D_LCO_. Predicted values were selected using a reference model by Zheng and associates.^60^ Short-acting bronchodilators, if any, were withdrawn for at least 4 h, and long-acting bronchodilators for 12 h prior to the examinations.^57^

BSI score and FACED score were applied to determine the severity and prognosis of bronchiectasis. The BSI score was a combination of clinical parameters, including the age, body mass index, prior exacerbations and prior hospitalization in the preceding year, mMRC grading, FEV_1_% of predicted, *Pseudomonas aeruginosa* infection, colonization with other potentially pathogenic microorganisms (PPMs, including *Haemophilus influenzae*, *Haemophilus parainfluenzae*, *Staphylococcus aureus*, *Klebsiella* spp., and other clinically significant bacteria) and the number of bronchiectatic lobes. BSI score of ≤4, 5–8, and ≥9 denoted mild, moderate, and severe bronchiectasis, respectively.^38^ FACED score incorporated variables including FEV_1_% of predicted, age, colonization of *Pseudomonas aeruginosa*, radiological extension, and mMRC grading. FACED score of ≤2, 3–4, and ≥5 denoted mild, moderate, and severe bronchiectasis, respectively.^37^

A standardized data collection spreadsheet was designed to obtain data of patients from electronic medical records. Two attending physicians independently reviewed the data collection forms to double check the data validity.

#### CT image analysis

All CT examinations were performed adhered to the common chest protocol: the patient was installed in a supine position with arms raised and held the breath at full inspiration during acquisition. Chest CT images were acquired using a GE Revolution APEX CT (GE Healthcare, Milwaukee, USA). The scan parameters are summarized as follows: helical, 100KVp, 80mm collimation, 0.5 s rotation time, 0.992 pitch, 1∼1.25mm slice thickness, B70f very sharp kernel. HRCT is critical to establish the diagnosis of bronchiectasis according to 2019 BTS guidelines.^29^ The direct signs of CT to establish a diagnosis of bronchiectasis include: (1) bronchial dilatation (internal lumen diameter greater than accompanying pulmonary artery, bronchoarterial ratio >1); (2) lack of airway tapering >2 cm distal to point of bifurcation; (3) airway visibility within 1 cm of the costal pleura of fissures. And the indirect signs include: (1) bronchial wall thickening; (2) mucoid impaction/fluid-filled airways (tubular or Y-shaped structures; branching or rounded opacities in cross section ± air-fluid levels); (3) bronchiolitis (clustered ill-defined centrilobular nodules with a tree-in-bud configuration); (4) mosaic attenuation caused by air trapping; (5) mosaic perfusion of the pulmonary identified on contrast-enhanced dual energy CT of the pulmonary parenchyma; (6) bronchial artery hyperplasia.^61^

CT image quantification and 3D visualization were performed with the open-source 3D Slicer, version 5.2.2 (https://www.slicer.org). Damaged lung areas with attenuation values of between −600 and 2500 Hounsfield units on CT images were automatically selected, with minor manual adjustment by experts. The percentage of damaged areas of the lungs is defined as the ratio to the total volume of both lungs (except trachea and bronchi) and is calculated by using the "Segment Statistics" function in the software.

### Method details

#### Immunostaining

For immunostaining, section slides underwent antigen retrieval in citrate buffer (pH = 6, Sigma-Aldrich, USA) heated in a microwave oven for 20 min. The following antibodies were utilized for immunostaining: KRT5 (1:500, MA5-14473, Thermo Fisher), P63 (1:200, ab735, Abcam), CD31 (1:200, Proteintech, 28083-1-AP), AQP5 (1:300, Abcam, ab92320). For immunofluorescence staining, Alexa-conjugated Donkey 488/594 secondary antibodies (1:200, Life Technologies, USA) along with DAPI (Roche, USA, 10236276001) were used. The tissue slides underwent auto-fluorescence removal and were mounted using mounting media (Vectashield, Vector Labs, H-1000-10). Slides were observed under a fluorescent microscope (Olympus).

#### Flow cytometry

Cells were digested into single cell suspensions, washed with PBS (Multicell, 311-425-CL), and then resuspended in PBS at a concentration of 1 × 10^6^ cells/mL. Flow cytometry staining was conducted in a standard protocol. Following staining, cells were transferred into FACS tubes and each tube was analyzed on a Beckman CytoFLEX within 1 h. The gate was defined to remove debris and doublet cells using FSC and SSC. Positive and negative cells were identified by the isotype control group. Antibodies used include: Anti-KRT5 (Abcam, Ab52635, 1:500), Alexa Fluor-conjugated Donkey 488 (Thermo Fisher, A21206, 1:200), PE Mouse Anti-Human CD45 Antibody (BD Pharmingen, 560975, 1:200), PE Mouse Anti-Human CD34 Antibody (BD Pharmingen, 560941, 1:200), FITC Mouse Anti-Human CD105 (BD Pharmingen, 561443, 1:200), FITC Mouse Anti-Human IgG Antibody (BD Pharmingen, 560952, 1:200), 488 Mouse IgG1 κ Isotype Ctrl Antibody (BD Pharmingen, 557782,1:200) and PE Mouse IgG1 κ Isotype Ctrl Antibody (BD Pharmingen, 557646,1:200).

#### Bulk RNA-Sequencing and bioinformatics

Total RNA was extracted from progenitor cells using TRIzol Reagent (Invitrogen, Life Technologies, 15596026CN) following the manufacturer’s instructions. Subsequently, the extracted RNA was treated with DNase I (Invitrogen, Life Technologies, USA) to remove any contaminating DNA. The cDNA library was then constructed and sequenced, and the BGI-NSG platform was utilized. The sequencing data obtained was then subjected to filtering using SOAP nuke.^62^ The filtering process involved the following steps: (1) removing reads containing sequencing adapters, (2) removing reads with a low-quality base ratio (base quality less than or equal to 15) higher than 20%, and (3) removing reads with an unknown base ('N' base) ratio higher than 5%. Following the filtering steps, clean reads were obtained and stored in FASTQ format for further analysis. The clean data were mapped to the reference genome (hGRC38) by HISAT (v2.1.0).^63^ The expression level of genes was calculated by RSEM (v1.2.8) and FPKM (Fragments Per Kilobase per Million) of each gene was calculated based on the length of the gene and read counts mapped to this gene.

Analysis of RNA-Seq data was performed by R (version 4.2.3). PCA and differential expression analysis were performed using the DESeq2 R package (1.38.3). A P-value of 0.01 and an absolute fold change of 2 were set as the threshold for significant differential expression. Visualization of heatmap was generated through R packet pheatmap (1.0.12). GO enrichment analysis of differentially expressed genes was performed by the ClusterProfiler R package. GO terms with a P-value <0.05 were considered significantly enriched by differentially expressed genes and the results were visualized by the enrichplot R package using dot plots. Protein-protein interaction (PPI) network was constructed to map the differentially expressed genes (DEGs) to the protein by using Cytoscape (3.10.0).

### Quantification and statistical analysis

As the trial was an early phase study, the sample size was based on clinical consideration, rather than statistical consideration, to provide safety and efficacy information with the need to minimize exposure to subjects. Categorical variables were presented as frequencies and percentages, while continuous variables were expressed as mean ± standard error of the mean (SEM)/standard deviation (SD) or median (25∼75^th^ interquartile range [IQR]). The Shapiro-Wilk test was applied to assess the data normality. The unpaired Student’s t test was used for normally distributed variables and the Mann-Whitney U test was used for non-normally distributed variables unless otherwise noted. Categorical variables were compared by the Chi-square test or Fisher’s exact test. Pearson correlation test was computed for correlation analysis. For the primary endpoint analysis, the AUC of the change from baseline to 4–24 weeks in D_LCO_ was calculated and Welch’s t-test was used to examine the difference between the cell treatment and control groups. For secondary endpoints, the difference between the cell treatment and control groups was tested using the Mann-Whitney U test, and the median differences were calculated using the Hodges-Lehmann estimation. If patients missed pulmonary function tests, the last results of the D_LCO_ test after cell treatment were carried forward to the missing visits for primary endpoint analysis. Other missing values for secondary endpoints and safety analyses were not imputed. Analyses were presented with two-sided P-values, with the level of significance set at 0.05. All statistical analysis and diagramming were performed by SPSS (version 25.0), GraphPad (version 9.0), and R package using ggplot2 (version 3.4.2).

### Additional resources

This study has been registered with ClinicalTrials.gov, NCT03655808.

## Supplemental information

Document S1. Figures S1–S11, Tables S1–S9, and Data S1

Document S2. Article plus supplemental information
