# Supplementary material for: Autologous transplantation of P63+ lung progenitor cells in patients with bronchiectasis: A randomized, single-blind, controlled trial
Source: Cell Rep Med. 2024 Nov 19;5(11):101819. doi: 10.1016/j.xcrm.2024.101819 (PMC11604513; doi:10.1016/j.xcrm.2024.101819)
Supplement: Document S1. Figures S1–S11, Tables S1–S9, and Data S1 [file mmc1.pdf]

Cell Reports Medicine, Volume *5*

Supplemental information

Autologous transplantation of $P63^+$ lung progenitor cells in patients with bronchiectasis: A randomized, single-blind, controlled trial

Jiayang Yan, Weipan Zhang, Yun Feng, Xuefei Liu, Lingyun Niu, Yi Guo, Ling Zhou, Mengmeng Shi, Caixia Di, Qiurui Zhang, Xiaofei Wang, Jianping Zhou, Ranran Dai, Lei Ni, Zhiyao Bao, Tianli Yan, Yun Hu, Ping Wang, Ting Zhang, Min Zhou, Wei Zuo, and Jieming Qu

Supplementary information

Table S1. Demographic and clinical characteristics of bronchiectasis patients who provided pulmonary tissues through surgical excision. Related to Figure 1.

	**Patient #1**	**Patient #2**	**Patient #3**	**Patient #4**	**Patient #5**
**Age (years)**	33	61	67	58	50
**Gender**	Male	Male	Female	Female	Female
**Body mass index (kg/m^2)**	25.95	22.99	27.25	24.56	24.63
**Smoking history (pack years)**	None	35	None	None	None
**Principal symptoms**	Cough, sputum, hemoptysis	Cough, sputum, hemoptysis	Cough, hemoptysis	Cough, chest pain	Cough, sputum, hemoptysis
**Duration of disease (years)**	17	12	1	1	2
**FEV_1 % predicted**	103.6	65.4	74.3	96.1	83.4
**Infection status**	*klebsiella pneumoniae*	*Streptococcus intermadius*	No bacterial growth in the sputum culture	No bacterial growth in the sputum culture	No bacterial growth in the sputum culture
**BSI score**	9	10	4	2	7
**Comorbidities**	None	Tuberculosis, gout	None	None	Tuberculosis
**Medication for bronchiectasis**	Inhaled bronchodilator	Mucolytics	Oral antibiotics, mucolytics	None	Oral antibiotics, mucolytics

Site of surgical tissues	Inferior lobe of left lung	Inferior lobe of left lung	Inferior lobe of left lung	Inferior lobe of right lung	Inferior lobe of right lung
**Airway destruction condition[a]**	Expanded alveolar spaces, alveolar histiocytosis, metaplasia	Expanded alveolar spaces, alveolar hemorrhage, focal abscess	Edema, alveolar hemorrhage and disruption, metaplasia	Alveolar histiocytosis, necrotic material in the bronchial lumen	Expanded alveolar spaces, alveolar hemorrhage
**Immune infiltration condition[a]**	Lymphocytes, plasma cells, and eosinophils infiltration	Lymphocytes infiltration, lymphoid follicular formation	Lymphocytes, plasma cells, and eosinophils infiltration, lymphoid follicular formation	Lymphocytes and plasma cells infiltration, lymphoid follicular formation	Lymphocytes, plasma cells, and eosinophils infiltration
**Fibrosis condition[a]**	Not observed	Interstitial fibrosis	Interstitial fibrosis	Interstitial fibrosis	Slight interstitial fibrosis

[a]These were assessed according to histopathological examination of specimens.

Table S2. List of key inclusion and exclusion criteria. Related to STAR Methods.

**Inclusion criteria:**
Aged between 18 to 75;
Remaining clinically stable;
Diagnosed with bronchiectasis according to the guidelines;
D_{LCO} < 80% of the predicted value;
Being capable of doing pulmonary function tests;
Tolerant to bronchofiberscopy;
Written informed consent signed.
**Exclusion criteria:**
Pregnant or lactating;
Patients positive for syphilis or HIV;
Patients with malignant tumors;
Patients with serious comorbidities;
Patients with serious systemic diseases;
Patients with serious kidney dysfunction;
Patients with serious liver dysfunction;
Patients with serious heart disease (NYHA class III~IV);
Patients with a history of abusing alcohol and illicit drugs;
Patients participated in other clinical trials in the past 3 months;
Patients could not understand the test procedures and use the test equipment;
Patients assessed as inappropriate to participate in this clinical trial by the investigator.

Table S3. Dose information in study patients. Related to STAR Methods.

Patient #	The date of $P63^{+}$ progenitor cells collection	Days from collection to transplantation (days)	Group	Dose ($\times 10^{6}$ cells/kg)[a]
**9001**	2020.8.12	28	Cell treatment	2.37
**9002**	2021.7.2	78	Cell treatment	1.78
**9007**	2021.5.7	46	Cell treatment	1.13
**9008**	2021.7.2	78	Cell treatment	2.49
**9010**	2021.9.16	132	Cell treatment	2.13
**9013**	2021.8.27	45	Cell treatment	2.12
**9015**	2021.10.19	42	Cell treatment	1.49
**9017**	2021.10.21	34	Cell treatment	1.70
**9018**	2021.11.11	48	Cell treatment	2.00
**9021**	2022.9.7	292	Cell treatment	2.40
**9024**	2022.3.7	74	Cell treatment	2.83
**9027**	2022.10.17	42	Cell treatment	2.28
**9028**	2022.8.15	31	Cell treatment	2.11
**9029**	2022.9.9	35	Cell treatment	2.34
**9032**	2022.10.13	49	Cell treatment	2.00
**9034**	2022.10.21	42	Cell treatment	2.41
**9035**	2022.10.27	48	Cell treatment	2.27
		67.29 ± 63.20[b]		2.11 ± 0.41[b]

[a]Cells suspended Perfedex preservation solution for long-term shipment.

[b]Data were presented as mean ± standard deviation (SD).

Table S4. Adverse events likely related to bronchoscopy. Related to Table 2.

	Any Grade[c]		Grade 1[c]		Grade 2[c]		Grade 3[c]		Grade 4~5[c]	
**Events**	Cell treatment (N = 17)	Control (N = 18)	Cell treatment (N = 17)	Control (N = 18)	Cell treatment (N = 17)	Control (N = 18)	Cell treatment (N = 17)	Control (N = 18)	Cell treatment (N = 17)	Control (N = 18)
**Fever[a]**	7(41.18%)	2 (11.11%)	4 (23.53%)	1 (5.56%)	3 (17.65%)	1 (5.56%)	0 (0)	0 (0)	0 (0)	0 (0)
**Hemoptysis[ab]**	3 (17.65%)	3 (16.67%)	3 (17.65%)	3 (16.67%)	0 (0)	0 (0)	0 (0)	0 (0)	0 (0)	0 (0)
**Sputum increased[a]**	1 (5.88%)	0 (0)	1 (5.88%)	0 (0)	0 (0)	0 (0)	0 (0)	0 (0)	0 (0)	0 (0)
**Pharyngeal discomfort[a]**	1 (5.88%)	3 (16.67%)	1 (5.88%)	3 (16.67%)	0 (0)	0 (0)	0 (0)	0 (0)	0 (0)	0 (0)
**Chest discomfort[a]**	1 (5.88%)	1 (5.56%)	0 (0)	1 (5.56%)	1 (5.88%)	0 (0)	0 (0)	0 (0)	0 (0)	0 (0)
**Dyspnea[a]**	1 (5.88%)	0 (0)	0 (0)	0 (0)	1 (5.88%)	0 (0)	0 (0)	0 (0)	0 (0)	0 (0)
**Nausea[a]**	1 (5.88%)	0 (0)	0 (0)	0 (0)	1 (5.88%)	0 (0)	0 (0)	0 (0)	0 (0)	0 (0)
**Pneumothorax[a]**	1 (5.88%)	0 (0)	0 (0)	0 (0)	0 (0)	0 (0)	1 (5.88%)	0 (0)	0 (0)	0 (0)

[a]Data were presented as patient number (percentage of patients).

[b]The term "hemoptysis" included bloody sputum in this study.

[c]The severity grade of adverse events was defined according to the National Cancer Institute Common Terminology Criteria for Adverse Events (NCI CTCAE), version 5.0.

Table S5. Adverse events likely unrelated to bronchoscopy. Related to Table 2.

Events	Any Grade[c]		Grade 1[c]		Grade 2[c]		Grade 3[c]		Grade 4~5[c]	
	Cell treatment (N = 17)	Control (N = 18)	Cell treatment (N = 17)	Control (N = 18)	Cell treatment (N = 17)	Control (N = 18)	Cell treatment (N = 17)	Control (N = 18)	Cell treatment (N = 17)	Control (N = 18)
**Fever[a]**	1 (5.88%)	3 (16.67%)	1 (5.88%)	0 (0)	1 (5.88%)	2 (11.11%)	0 (0)	0 (0)	0 (0)	0 (0)
**Hemoptysis[ab]**	0 (0)	3 (16.67%)	0 (0)	3 (16.67%)	0 (0)	0 (0)	0 (0)	0 (0)	0 (0)	0 (0)
**Sputum increased[a]**	3 (17.65%)	3 (16.67%)	3 (17.65%)	3 (16.67%)	0 (0)	0 (0)	0 (0)	0 (0)	0 (0)	0 (0)
**Cough increased[a]**	3 (17.65%)	3 (16.67%)	3 (17.65%)	3 (16.67%)	0 (0)	0 (0)	0 (0)	0 (0)	0 (0)	0 (0)
**Fatigue[a]**	3 (17.65%)	3 (16.67%)	2 (11.76%)	2 (11.11%)	1 (5.88%)	1 (5.56%)	0 (0)	0 (0)	0 (0)	0 (0)
**COVID-19[a]**	2 (11.76%)	4 (22.22%)	0 (0)	0 (0)	2 (11.76%)	4 (22.22%)	0 (0)	0 (0)	0 (0)	0 (0)
**Bronchiectasis exacerbation[a]**	2 (11.76%)	2 (11.11%)	0 (0)	0 (0)	2 (11.76%)	2 (11.11%)	0 (0)	0 (0)	0 (0)	0 (0)
**Dizziness[a]**	1 (5.88%)	1 (5.56%)	1 (5.88%)	1 (5.56%)	0 (0)	0 (0)	0 (0)	0 (0)	0 (0)	0 (0)
**Dyspnea[a]**	0 (0)	1 (5.56%)	0 (0)	1 (5.56%)	0 (0)	0 (0)	0 (0)	0 (0)	0 (0)	0 (0)
**Influenza[a]**	0 (0)	1 (5.56%)	0 (0)	0 (0)	0 (0)	1 (5.56%)	0 (0)	0 (0)	0 (0)	0 (0)
**Anxiety[a]**	0 (0)	1 (5.56%)	0 (0)	0 (0)	0 (0)	1 (5.56%)	0 (0)	0 (0)	0 (0)	0 (0)
**Acute exacerbation of COPD[a]**	1 (5.88%)	0 (0)	0 (0)	0 (0)	0 (0)	0 (0)	1 (5.88%)	0 (0)	0 (0)	0 (0)

[a]Data were presented as patient number (percentage of patients).

[b]The term "hemoptysis" included bloody sputum in this study.

[c]Severity grade of adverse events were defined according to the National Cancer Institute Common Terminology Criteria for Adverse Events (NCI CTCAE), version 5.0.

Table S6. Number and percentage of patients who improved or deteriorated more than the minimal clinically important difference in two groups for D_{LCO}, FEV_1, 6MWD, SGRQ score, and mMRC grading at Week 4, 12, and 24. Related to Figure 2.

	Cell treatment group	Control group	P-value
**D_{LCO} % predicted change percentage from baseline**			
Week 4	*N* = 13	*N* = 18	
> 10%[a]	4 (30.8%)	0 (0)	
-10% to 10%[a]	7 (53.8%)	9 (50.0%)	**0.017**
< -10%[a]	2 (15.4%)	9 (50.0%)	
Week 12	*N* =13	*N* =18	
> 10%[a]	5 (38.5%)	2 (11.1%)	
-10% to 10%[a]	5 (38.5%)	10 (55.6%)	0.237
< -10%[a]	3 (23.1%)	6 (33.3%)	
Week 24	*N* = 12	*N* = 18	
> 10%[a]	3 (25.0%)	3 (16.7%)	
-10% to 10%[a]	6 (50.0%)	12 (66.7%)	0.665
< -10%[a]	3 (25.0%)	3 (16.7%)	
**FEV_1 change from baseline**			
Week 4	*N* = 13	*N* = 17	
> 100 mL[a]	2 (15.4%)	1 (5.9%)	
-100 to 100 mL[a]	9 (69.2%)	11 (64.7%)	0.535
<100 mL[a]	2 (15.4%)	5 (29.4%)	
Week 12	*N* = 10	*N* = 15	
> 100 mL[a]	0 (0)	3 (20.0%)	
-100 to 100 mL[a]	9 (90.0%)	7 (46.7%)	0.120
<100 mL[a]	1 (10.0%)	5 (33.3%)	
Week 24	*N* = 11	*N* = 18	
> 100 mL[a]	0 (0)	4 (22.2%)	
-100 to 100 mL[a]	8 (72.7%)	9 (50.0%)	0.285
<100 mL[a]	3 (27.3%)	5 (27.8%)	
**6MWD change from baseline**			
Week 4	*N* = 12	*N* = 17	
> 30 m[a]	5 (41.7%)	5 (29.4%)	
-30 to 30 m[a]	6 (50.0%)	8 (47.1%)	0.621
<30 m[a]	1 (8.3%)	4 (23.5%)	
Week 12	*N* = 10	*N* = 15	
> 30 m[a]	5 (50.0%)	6 (40.0%)	0.512

-30 to 30 m[a]	4 (40.0%)	4 (26.7%)	
<30 m[a]	1 (10.0%)	5 (33.3%)	
Week 24	*N* = 11	*N* = 18	
> 30 m[a]	5 (45.5%)	5 (27.8%)	
-30 to 30 m[a]	4 (36.4%)	6 (33.3%)	0.563
<30 m[a]	2 (18.2%)	7 (38.9%)	
**SGRQ score change from baseline**			
Week 4	*N* = 13	*N* = 17	
> 4 units[a]	10 (76.9%)	7 (41.2%)	
-4 to 4 units[a]	0 (0)	6 (35.3%)	**0.049**
< -4 units[a]	3 (23.1%)	4 (23.5%)	
Week 12	*N* = 10	*N* = 15	
> 4 units[a]	7 (70.0%)	6 (40.0%)	
-4 to 4 units[a]	0 (0)	6 (40.0%)	0.102
< -4 units[a]	3 (30.0%)	3 (20.0%)	
Week 24	*N* = 11	*N* = 18	
> 4 units[a]	4 (36.4%)	10 (55.6%)	
-4 to 4 units[a]	4 (36.4%)	3 (16.7%)	0.485
< -4 units[a]	3 (27.3%)	5 (27.8%)	
**mMRC grading change from baseline**			
Week 4	*N* = 13	*N* = 17	
> 1 unit[a]	6 (46.2%)	4 (23.5%)	
-1 to 1 unit[a]	6 (46.2%)	11 (64.7%)	0.423
< -1 unit[a]	1 (7.7%)	2 (11.8%)	
Week 12	*N* = 10	*N* = 15	
> 1 unit[a]	7 (70.0%)	0 (0)	
-1 to 1 unit[a]	1 (10.0%)	10 (66.7%)	**<0.0001**
< -1 unit[a]	2 (20.0%)	5 (33.3%)	
Week 24	*N* = 11	*N* = 18	
> 1 unit[a]	3 (27.3%)	5 (27.8%)	
-1 to 1 unit[a]	6 (54.5%)	11 (61.1%)	0.874
< -1 unit[a]	2 (18.3%)	2 (11.1%)	

[a]Data were presented as patient number (percentage of patients).

Table S7. Key secondary endpoint results at Week 4, 12, and 24. Related to Figure 2 and Figure S10.

Endpoint	Cell treatment group		Control group		Difference in change, Cell treatment vs Control group (95% CI) [c]	P-value
	N subjects [a]	Change from baseline[b]	N subjects[a]	Change from baseline[b]		
**Week 4**						
VA (L)	13	0.08 (-0.18, 0.40)	15	-0.08 (-0.42, 0.07)	0.26 (0.01, 0.89)	**0.037**
VA % predicted	13	2.60 (-3.00, 7.55)	15	-1.70 (-8.80, 1.50)	4.90 (0.20, 15.00)	**0.033**
TLC % predicted	13	2.90 (-3.25, 11.40)	17	-1.80 (-10.05, -0.05)	5.80 (0.10, 18.30)	**0.043**
FEV_1 (L)	13	0.01 (-0.07, 0.06)	17	0 (-0.14, 0.04)	0.03 (-0.04, 0.12)	0.245
FEV_1 % predicted	13	1.90 (-0.70, 3.00)	17	0.20 (-4.85, 1.10)	2.10 (-0.50, 5.70)	0.079
FVC (L)	13	-0.02 (-0.12, 0.15)	17	-0.05 (-0.13, 0.04)	0.04 (-0.06, 0.18)	0.385
FVC % predicted	13	0.70 (-3.30, 4.55)	17	-1.20 (-5.45, 0.95)	2.40 (-1.40, 6.20)	0.213
FEV_1/FVC	13	0.01 (-5.48, 2.66)	17	-2.31 (-4.32, 2.02)	0.38 (-4.05, 4.13)	0.805
MMEF (L/s)	13	0 (-0.03, 0.05)	17	-0.07 (-0.28, 0.06)	0.07 (-0.05, 0.26)	0. 363
MVV (L/min)	12	0.83 (-3.19, 7.26)	16	3.95 (0, 6.31)	-1.61 (-6.20, 8.02)	0.599
6MWD (m)	12	20.50 (-1.00, 85.91)	17	2.73 (-32.85, 46.35)	29.84 (-18.00, 79.00)	0.263
DSP	12	35.40 (0.21, 81.00)	16	17.51 (-10.86, 30.74)	25.45 (-16.03,72.77)	0.302
SGRQ	13	-16.65 (-21.05, 0.73)	17	-1.27 (-8.60, 4.42)	-11.84 (-19.60, 1.69)	0.103
BSI score	13	-2.00	17	0 (-0.50, 0)	-2.00 (-3.00, 0)	**0.028**

		(-3.00, 0)				
FACED score	13	-1.00 (-1.00, 0)	17	0 (0, 0)	-1.00 (-1.00, 0)	**0.048**
mMRC grading	13	0 (-1.00, 0)	17	0 (-0.50, 0)	0 (-1.00, 0)	0.263
**Week 12**						
FEV_1 (L)	10	0 (-0.08, 0.03)	15	-0.03 (-0.12, 0.08)	0.03 (-0.08, 0.11)	0.495
FEV_1 % predicted	10	-0.05 (-3.60, 1.30)	15	-1.90 (-6.30, 3.31)	1.00 (-3.70, 5.40)	0.765
FVC (L)	10	0.06 (-0.05, 0.08)	15	-0.06 (-0.29, 0.12)	0.12 (-0.06, 0.28)	0.129
FVC % predicted	10	1.00 (-2.38, 2.78)	15	-4.00 (-11.30, 3.60)	3.50 (-2.10, 10.40)	0.196
FEV_1/FVC	10	-2.97 (-4.76, 2.27)	15	0.83 (-0.29, 3.09)	-3.49 (-6.34, 0.69)	0.091
MMEF (L/s)	9	-0.06 (-0.17, -0.01)	15	0 (-0.17, 0.07)	-0.05 (-0.21, 0.07)	0.263
MVV (L/min)	10	2.37 (-1.95, 12.50)	15	-0.15 (-4.74, 12.00)	3.18 (-6.52, 14.32)	0.531
6MWD (m)	10	40.96 (-12.75, 105.50)	15	24.00 (-51.00, 57.00)	38.34 (-36.00, 107.00)	0.261
DSP	9	62.56 (26.85, 85.18)	13	24.09 (-15.86, 42.15)	39.74 (-8.47, 76.08)	0.110
SGRQ	10	-7.29 (-16.18, 10.16)	15	-1.68 (-16.92, 3.16)	-4.09 (-12.93, 10.89)	0.807
BSI score	10	-1.50 (-3.50, 0)	15	0 (-1.00, 1.00)	-2.00 (-4.00, 0)	**0.016**
FACED score	10	-0.50 (-1.00, 0)	15	0 (0, 0)	-1.00 (-1.00, 0)	**0.023**
mMRC grading	10	-1.00 (-1.00, 0.25)	15	0 (0, 1.00)	-1.00 (-1.00, -1.00)	**0.012**
**Week 24**						
IC (L)	10	0.12	13	-0.12	0.22 (0.04, 0.48)	**0.018**

		(-0.10, 0.28)		(-0.26, 0.02)		
FEV_1 (L)	11	0 (-0.10, 0.02)	18	-0.01 (-0.11, 0.08)	0.01 (-0.10, 0.08)	0.774
FEV_1 % predicted	11	-0.30 (-4.40, 1.10)	18	-0.90 (-5.40, 4.40)	-0.25 (-5.20, 3.70)	0.912
FVC (L)	11	0.03 (-0.13, 0.09)	18	0.01 (-0.25, 0.11)	0.04 (-0.15, 0.18)	0.642
FVC % predicted	11	-0.40 (-2.10, 1.70)	18	-1.65 (-9.53, 2.93)	1.00 (-3.90, 4.60)	0.642
FEV_1/FVC	11	0.99 (-3.46, 0.85)	17	-0.01 (4.43, 3.05)	-0.97 (-4.48, 2.39)	0.547
MMEF (L/s)	11	-0.01 (-0.08, 0.04)	17	0 (-0.18, 0.06)	-0.01 (-0.15, 0.09)	0.890
MVV (L/min)	10	0.83 (-4.30, 8.45)	15	0.96 (-6.21, 7.87)	0.24 (-7.20, 8.07)	0.935
6MWD (m)	11	9.00 (-27.00, 100.80)	18	-0.57 (-59.88, 34.25)	42.12 (-28.46, 106.36)	0.276
DSP	11	44.83 (-33.64, 77.49)	15	0.20 (-53.73, 38.54)	29.06 (-21.95, 88.83)	0.198
SGRQ	11	-2.22 (-20.03, 10.06)	18	-6.13 (-11.17, 7.12)	-0.06 (-12.47, 12.56)	> 0.999
BSI score	11	0 (-3.00, 0)	18	0 (-0.25,1.00)	-1.00 (-3.00, 0)	0.092
FACED score	11	0 (-1.00, 0)	18	0 (0, 0)	0 (-1.00, 0)	0.238
mMRC grading	11	0 (-1.00, 0)	18	0 (-1.00, 0)	0 (-1.00, 1.00)	0.808

CI, confidence interval; VA, alveolar ventilation; TLC, total lung capacity; MMEF, maximum mid expiratory flow; MVV, maximum voluntary ventilation; DSP, distance-saturation product; IC, inspiratory capacity.

[a]Patients No. at Week 4/12/24.

[b]Data are shown as median (interquartile range, IQR).

[c]Differences are expressed as Hodges-Lehmann estimator and 95% CI.

Table S8. Number and percentage of patients who improved or deteriorated in the individual components of BSI score and FACED score at Week 4, 12, and 24. Related to Figure 2 and Figure S6, S7, S8.

	Cell treatment group	Control group	P-value
**BSI-the BMI parameter change from baseline**			
Week 4	*N* = 13	*N* = 17	
Improvement[a]	1 (7.7%)	0 (0)	
No change[a]	12 (92.3%)	17 (100%)	0.433
Worsening[a]	0 (0)	0 (0)	
Week 12	*N* =10	*N* =15	
Improvement[a]	1 (10.0%)	0 (0)	
No change[a]	9 (90.0%)	15 (100%)	0.400
Worsening[a]	0 (0)	0 (0)	
Week 24	*N* = 11	*N* = 18	
Improvement[a]	1 (9.1%)	0 (0)	
No change[a]	10 (90.9%)	18 (100%)	0.379
Worsening[a]	0 (0)	0 (0)	
**BSI-the FEV_1 % of predicted parameter change from baseline**			
Week 4	*N* = 13	*N* = 17	
Improvement[a]	0 (0)	2 (11.8%)	
No change[a]	13 (100%)	11 (64.7%)	0.068
Worsening[a]	0 (0)	4 (23.5%)	
Week 12	*N* =10	*N* =15	
Improvement[a]	1 (10.0%)	2 (13.3%)	
No change[a]	9 (90.0%)	11 (73.3%)	0.769
Worsening[a]	0 (0)	2 (13.3%)	
Week 24	*N* = 11	*N* = 18	
Improvement[a]	0 (0)	2 (11.1%)	
No change[a]	10 (100%)	13 (72.2%)	0.499
Worsening[a]	1 (9.1%)	3 (16.7%)	
**BSI-the acute exacerbation parameter change from baseline**			
Week 12	*N* =10	*N* =15	
Improvement[a]	0 (0)	0 (0)	
No change[a]	10 (100%)	14 (93.3%)	> 0.999
Worsening[a]	0 (0)	1 (6.7%)	
Week 24	*N* = 11	*N* = 18	
Improvement[a]	0 (0)	0 (0)	> 0.999

No change[a]	11 (100%)	17(94.4%)	
Worsening[a]	0 (0)	1 (5.6%)	
**BSI-the mMRC grading parameter change from baseline**			
Week 4	*N* = 13	*N* = 17	
Improvement[a]	4 (30.8%)	1 (5.9%)	
No change[a]	9 (69.2%)	15 (88.2%)	0.138
Worsening[a]	0 (0)	1 (5.9%)	
Week 12	*N* =10	*N* =15	
Improvement[a]	2 (20.0%)	0 (0)	
No change[a]	8 (80.0%)	13 (86.7%)	0.141
Worsening[a]	0 (0)	2 (13.3%)	
Week 24	*N* = 11	*N* = 18	
Improvement[a]	1 (9.1%)	1 (5.6%)	
No change[a]	8 (72.7%)	17 (94.4%)	0.257
Worsening[a]	2 (18.2%)	0 (0)	
**BSI-the *Pseudomonas aeruginosa* infection parameter change from baseline**			
Week 4	*N* = 13	*N* = 17	
Improvement[a]	4 (30.8%)	2 (11.8%)	
No change[a]	9 (69.2%)	15 (88.2%)	0.360
Worsening[a]	0 (0)	0 (0)	
Week 12	*N* =10	*N* =15	
Improvement[a]	4 (40.0%)	1 (6.7%)	
No change[a]	6 (60.0%)	13 (86.7%)	0.121
Worsening[a]	0 (0)	1 (6.7%)	
Week 24	*N* = 11	*N* = 18	
Improvement[a]	4 (36.4%)	3 (16.7%)	
No change[a]	7 (63.6%)	12 (66.7%)	0.264
Worsening[a]	0 (0)	3 (16.7%)	
**BSI-the other microorganisms parameter change from baseline[b]**			
Week 12	*N* =10	*N* =15	
Improvement[a]	0 (0)	1 (6.7%)	
No change[a]	10 (100%)	14 (93.3%)	> 0.999
Worsening[a]	0 (0)	0 (0)	
Week 24	*N* = 11	*N* = 18	
Improvement[a]	0 (0)	0 (0)	
No change[a]	11 (100%)	17(94.4%)	> 0.999
Worsening[a]	0 (0)	1 (5.6%)	
**FACED-the *Pseudomonas aeruginosa* infection parameter change from baseline**			

Week 4	$N = 13$	$N = 17$	
Improvement[a]	4 (30.8%)	2 (11.8%)	
No change[a]	9 (69.2%)	15 (88.2%)	0.360
Worsening[a]	0 (0)	0 (0)	
Week 12	$N = 10$	$N = 15$	
Improvement[a]	4 (40.0%)	1 (6.7%)	
No change[a]	6 (60.0%)	13 (86.7%)	0.121
Worsening[a]	0 (0)	1 (6.7%)	
Week 24	$N = 11$	$N = 18$	
Improvement[a]	4 (36.4%)	3 (16.7%)	
No change[a]	7 (63.6%)	12 (66.7%)	0.264
Worsening[a]	0 (0)	3 (16.7%)	
**FACED-the mMRC grading parameter change from baseline**			
Week 4	$N = 13$	$N = 17$	
Improvement[a]	4 (30.8%)	1 (5.9%)	
No change[a]	9 (69.2%)	15 (88.2%)	0.138
Worsening[a]	0 (0)	1 (5.9%)	
Week 12	$N = 10$	$N = 15$	
Improvement[a]	2 (20.0%)	0 (0)	
No change[a]	8 (80.0%)	13 (86.7%)	0.141
Worsening[a]	0 (0)	2 (13.3%)	
Week 24	$N = 11$	$N = 18$	
Improvement[a]	1 (9.1%)	1 (5.6%)	
No change[a]	9 (81.8%)	17 (94.4%)	0.470
Worsening[a]	1 (9.1%)	0 (0)	
**FACED-the FEV_1 % of predicted parameter change from baseline[c]**			
Week 4	$N = 13$	$N = 17$	
Improvement[a]	0 (0)	0 (0)	
No change[a]	13 (100%)	16 (94.1%)	> 0.999
Worsening[a]	0 (0)	1 (5.9%)	

[a]Data were presented as patient number (percentage of patients).

[b]Data of the age, hospitalization, and radiological severity parameters of BSI score at Week 4, 12, and 24, and the other microorganisms parameter at Week 4 were not shown as all patients had no change.

[c]Data of the age and radiological severity parameters of FACED score at Week 4, 12, and 24, and the FEV_1 % of predicted parameter at Week 12 and 24 were not shown as all patients had no change.

Table S9. Baseline characteristics of complete responsive (CR) and non-responsive (NR) patients in the cell treatment group. Related to Figure 4.

Demographics	CR patients ($N = 4$)	NR patients ($N = 3$)	P-value
Age (years)[a]	50.5 ± 14.8	49.0 ± 10.5	0.888
Female gender[b]	3 (75.0%)	2 (66.7%)	> 0.999
Body mass index (kg/m^2)[a]	23.3 ± 1.7	22.3 ± 4.2	0.669
Smokers[b]	0 (0)	1 (33.3%)	0.429
**Bronchiectasis characteristics**			
Duration of disease (years)[a]	22.0 ± 18.7	14.3 ± 6.7	0.535
FEV_1 (L)[a]	1.0 ± 0.4	1.6 ± 0.7	0.174
D_{LCO} (mmol/min/kPa)[a]	4.6 ± 0.6	6.1 ± 1.3	0.085
6MWD (m)[a]	443.5 ± 123.5	411.0 ± 92.7	0.720
SGRQ score[a]	45.9 ± 13.7	51.1 ± 22.4	0.718
BSI score[a]	8.3 ± 4.0	10.8 ± 1.5	0.311
FACED score[a]	3.5 ± 1.3	2.3 ± 1.5	0.322
mMRC grading[c]	1.5 (1.0, 2.5)	2.0 (2.0, 2.5)	0.400
**Etiology**			
Post infection[b]	1 (25.0%)	1 (33.3%)	> 0.999
Idiopathic[b]	3 (75.1%)	2 (66.7%)	
**Comorbidities**			
COPD[b]	1 (25.0%)	1 (33.3%)	> 0.999
Chronic rhinitis or sinusitis[b]	0 (0)	1 (33.3%)	0.429
Hypertension[b]	1 (25.0%)	0 (0)	> 0.999
Diabetes[b]	0 (0)	1 (33.3%)	0.429
**Quality BALF culture**			
Pseudomonas aeruginosa[b]	3 (75.0%)	1 (33.3%)	0.486
No bacterial growth[b]	1 (25.0%)	2 (66.7%)	
**Medication for bronchiectasis**			
Oral antibiotics[b]	3 (75.0%)	2 (66.7%)	> 0.999
Inhaled bronchodilator[b]	3 (75.0%)	1 (33.3%)	0.486
Mucolytics[b]	2 (50.0%)	2 (66.7%)	> 0.999

[a]Data were presented as mean ± standard deviation (SD).

[b]Data were presented as patient number (percentage of patients).

[c]Data were presented as median (interquartile range, IQR).

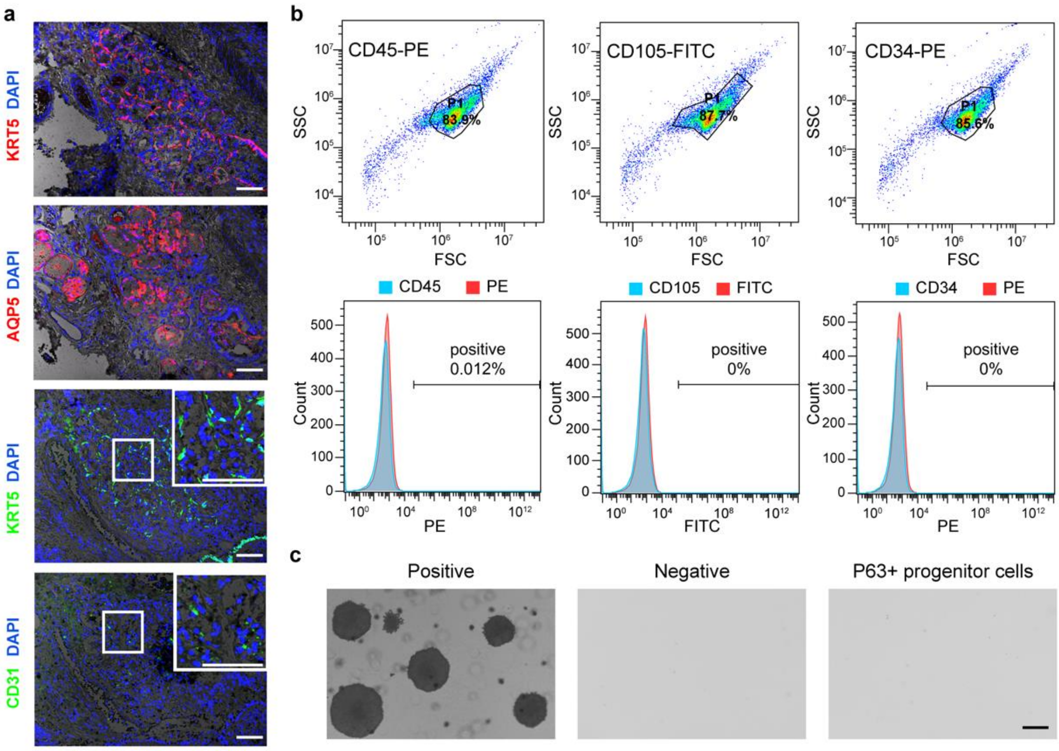

Fig. S1 Immunofluorescence images and quality control of cultured lung progenitor cells. Related to Figure 1. a, Immunofluorescence staining showing the expression of KRT5, AQP5, and CD31 with nuclear staining using DAPI (blue). KRT5 expression (red) and AQP5 expression (red) are shown in the same field for Patient #5. KRT5 expression (green) and CD31 expression (green) are shown in the same field for Patient #3. Scale bar, 50 µm. **b,** FACS gating strategy for purity test by immunostaining with anti-CD45, CD105, and CD34 antibodies. **c,** Soft agar assay, also known as a tumorigenicity test, showing the lack of tumor formation by $P63^+$ lung progenitor cells. Human melanoma cells served as a positive control, while growth-arrested 3T3 cells served as a negative control. Scale bar, 200 µm.

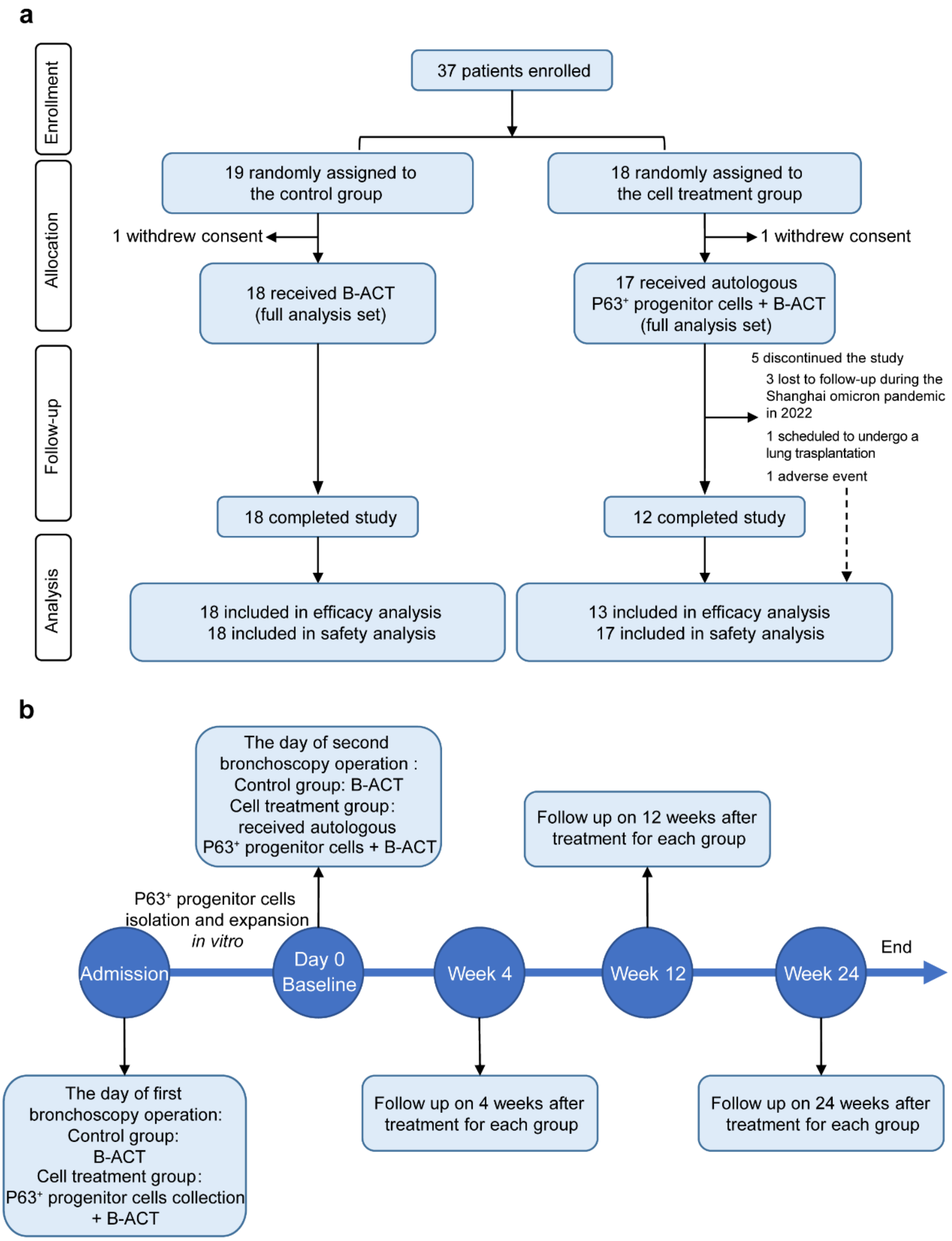

Fig. S2 Flow diagram and timeline for the study design. Related to STAR Methods. a, The detailed procedure of the study. **b**, The timeline of the study. B-ACT, bronchoscopic airway clearance therapy.

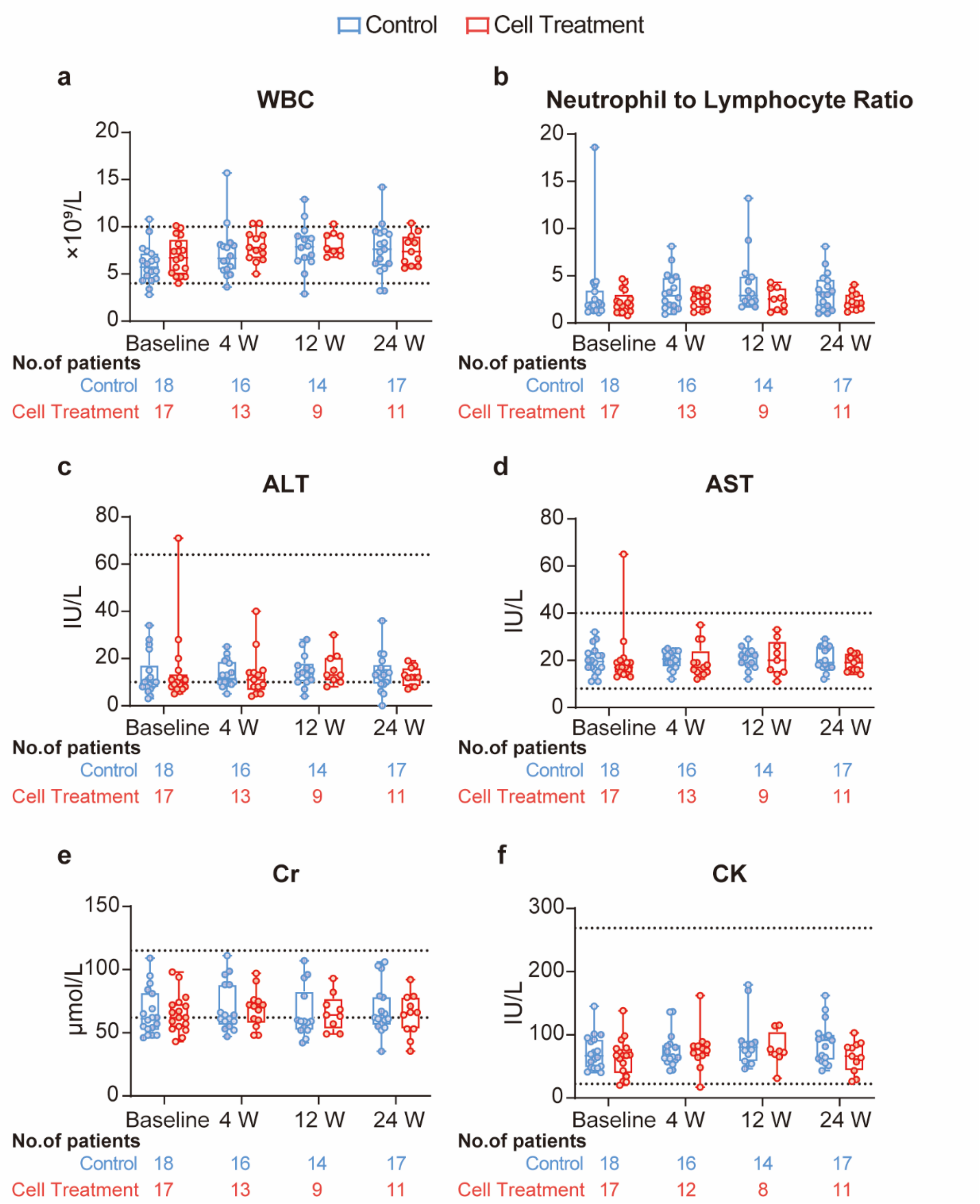

Fig. S3 Boxplot showing changes in clinical laboratory evaluations following cell therapy. Related to Table 2. The horizontal line within each box represents the median value; the bottom and top lines of the box represent the 25th and 75th percentiles, respectively; and the horizontal lines below and above the box represent the lowest and highest values, respectively.

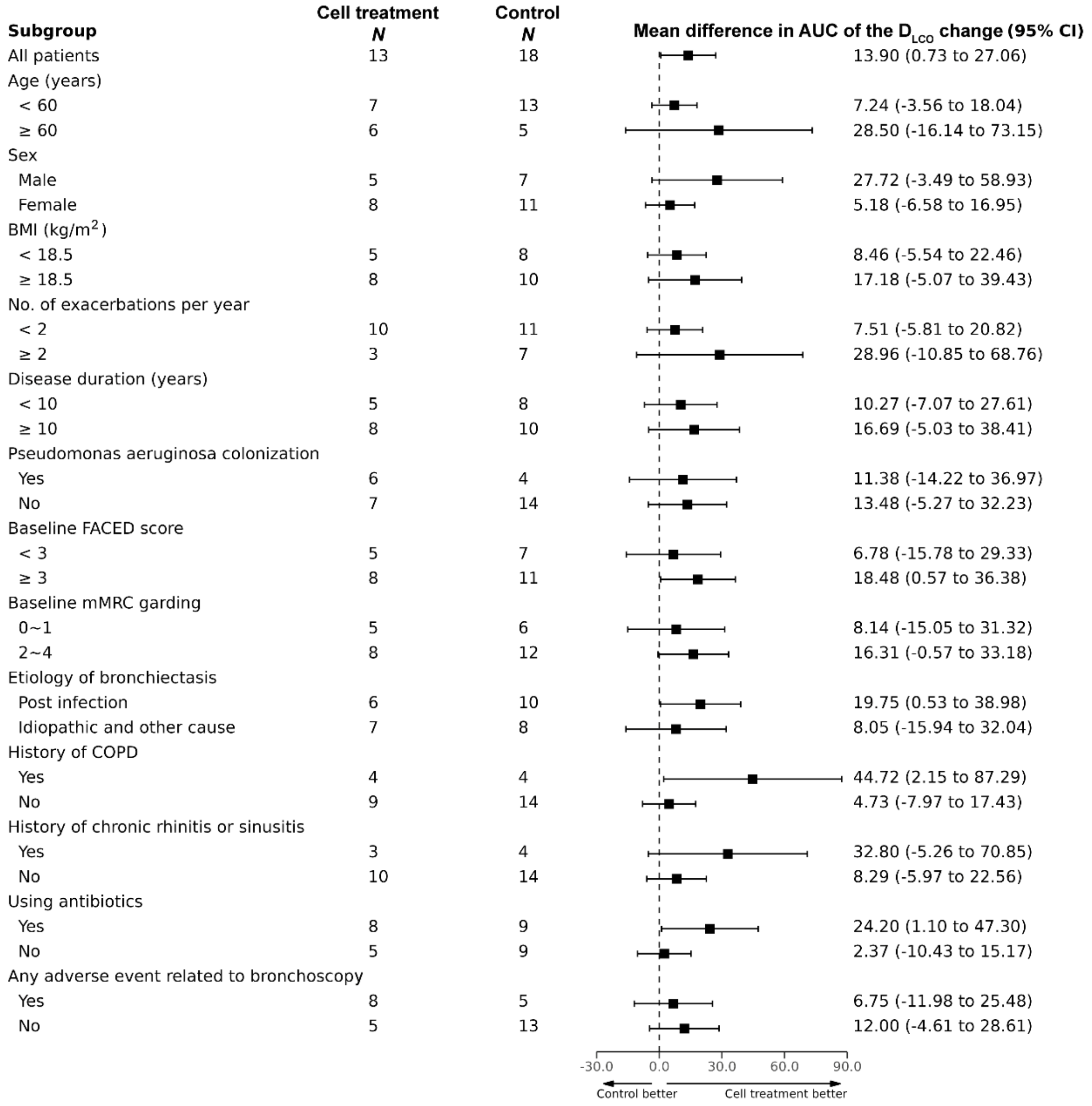

Fig. S4 Forrest plots of the AUC of the D_{LCO} change from baseline to 24 weeks in subgroup analysis. Related to Figure 2. AUC, the area under the curve; BMI, body mass index; mMRC, modified British Medical Research Council; COPD, chronic obstructive pulmonary disease.

Subgroup	Cell treatment *N*	Control *N*	Mean difference in AUC of the D_{LCO} % change (95% CI)
All patients	13	18	161.83 (7.23 to 316.44)
Age (years)			
< 60	7	13	85.39 (-46.62 to 217.39)
≥ 60	6	5	332.55 (-185.30 to 850.41)
Sex			
Male	5	7	329.35 (-26.93 to 685.62)
Female	8	11	56.08 (-89.66 to 201.83)
BMI (kg/m^2)			
< 18.5	5	8	115.32 (-46.86 to 277.48)
≥ 18.5	8	10	187.09 (-75.64 to 449.83)
No. of exacerbations per year			
< 2	10	11	98.72 (-69.21 to 266.65)
≥ 2	3	7	315.47 (-106.23 to 737.18)
Disease duration (years)			
< 10	5	8	128.56 (-88.83 to 344.95)
≥ 10	8	10	189.48 (-61.64 to 440.60)
Pseudomonas aeruginosa colonization			
Yes	6	4	161.64 (-76.10 to 399.37)
No	7	14	146.08 (-67.44 to 359.61)
Baseline FACED score			
< 3	5	7	65.85 (-182.62 to 314.33)
≥ 3	8	11	223.47 (14.16 to 432.78)
Baseline mMRC garding			
0~1	5	6	79.09 (-190.56 to 348.54)
2~4	8	12	198.62 (0.93 to 396.32)
Etiology of bronchiectasis			
Post infection	6	10	233.63 (9.61 to 475.65)
Idiopathic and other cause	7	8	121.35 (-182.36 to 362.92)
History of COPD			
Yes	4	4	538.50 (42.47 to 1034.53)
No	9	14	47.61 (-98.90 to 194.12)
History of chronic rhinitis or sinusitis			
Yes	3	4	423.16 (-21.07 to 867.38)
No	10	14	84.69 (-81.96 to 251.33)
Using antibiotics			
Yes	8	9	279.12 (9.08 to 549.15)
No	5	9	30.75 (-126.66 to 188.17)
Any adverse event related to bronchoscopy			
Yes	8	5	64.73 (-148.56 to 278.03)
No	5	13	137.45 (-57.55 to 332.45)

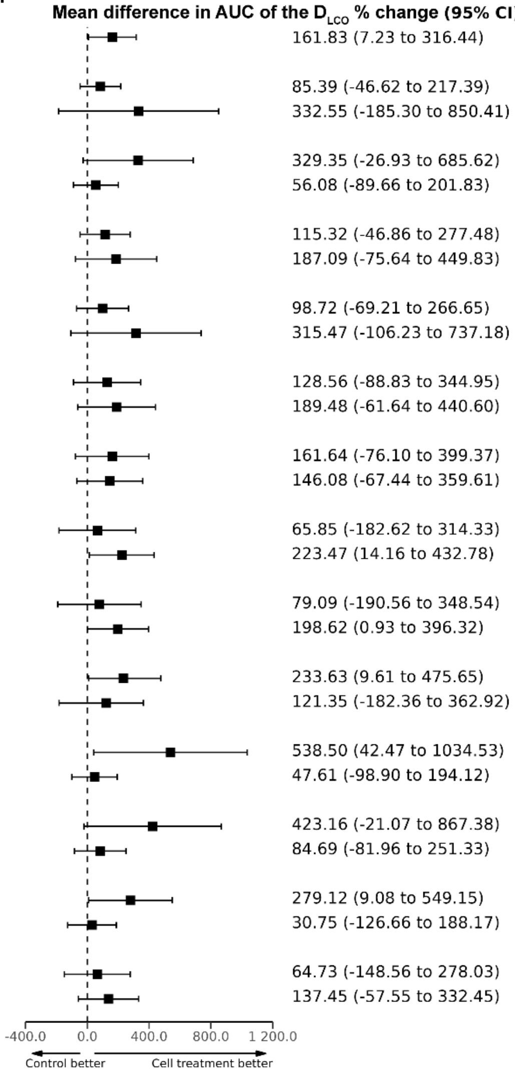

Fig. S5 Forrest plots of the AUC of the D_{LCO} % of predicted value change from baseline to 24 weeks in subgroup analysis. Related to Figure 2. AUC, the area under the curve; BMI, body mass index; mMRC, modified British Medical Research Council; COPD, chronic obstructive pulmonary disease.

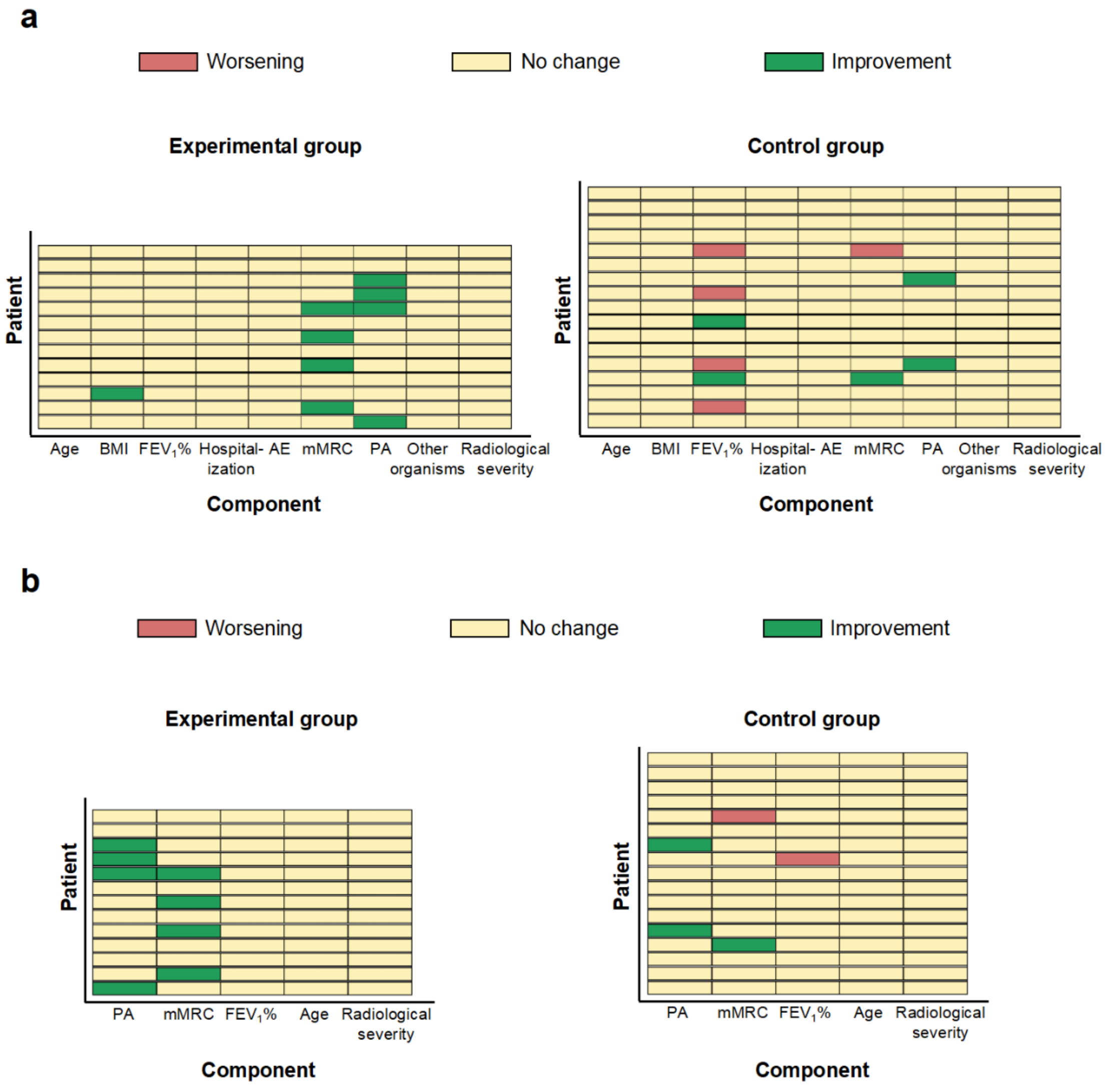

Fig. S6 Components of BSI and FACED scores at Week 4 for patients in two groups. Related to Figure 2 and Table S8. Components of BSI score (**a**) and FACED score (**b**) at Week 4 for patients in the experimental group (left) (n = 13) and the control group (right) (n = 17). Each row represents an individual patient. Green represents improvement, yellow no change, and red worsening, as compared with baseline values. BMI, body mass index; FEV_1, forced expiratory volume in 1 second; AE, acute exacerbation; mMRC, modified British Medical Research Council; PA, *Pseudomonas aeruginosa*.

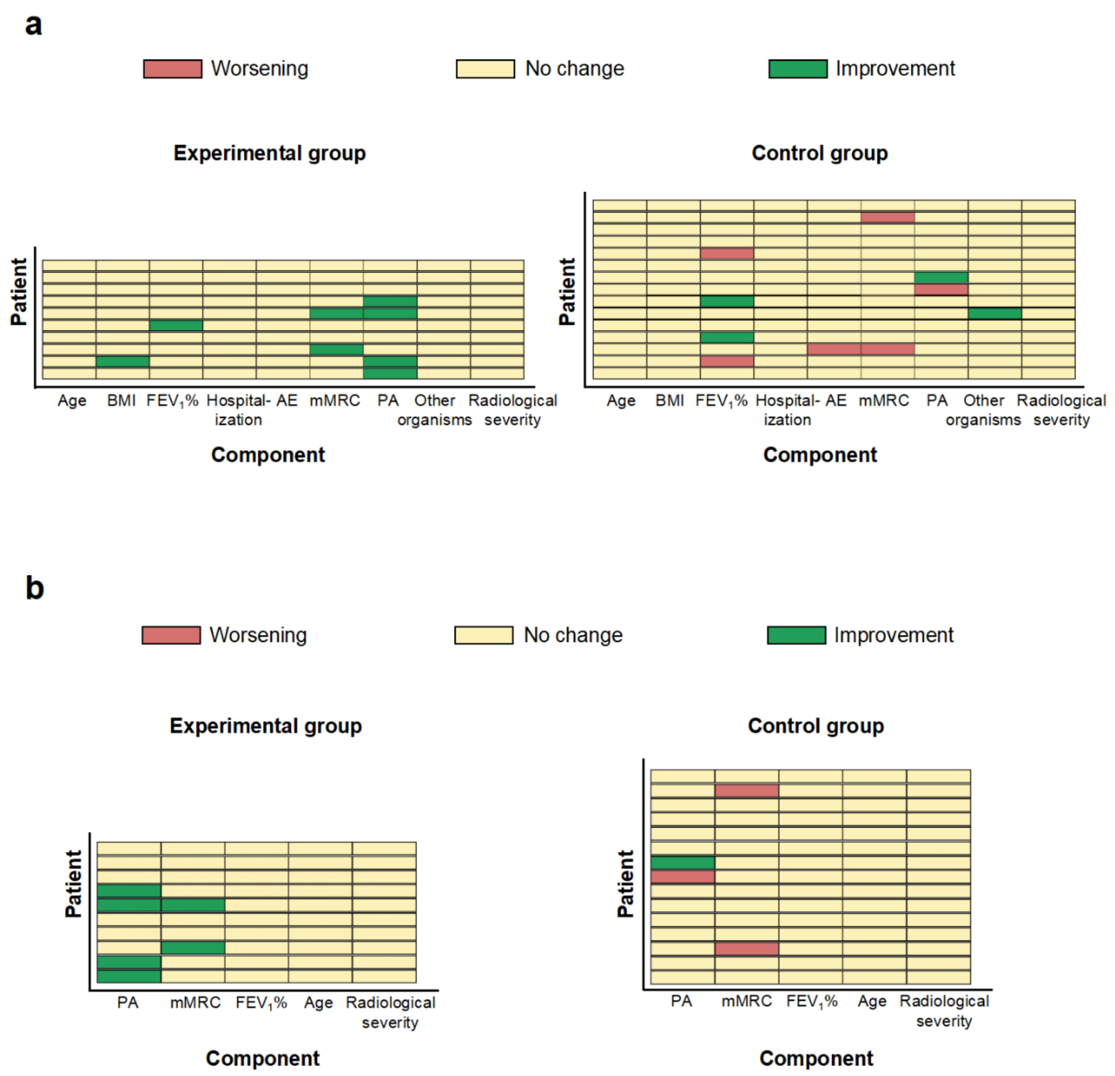

Fig. S7 Components of BSI and FACED scores at Week 12 for patients in two groups. Related to Figure 2 and Table S8. Components of BSI score (**a**) and FACED score (**b**) at Week 12 for patients in the experimental group (left) (n = 10) and the control group (right) (n = 15). Each row represents an individual patient. Green represents improvement, yellow no change, and red worsening, as compared with baseline values. BMI, body mass index; FEV_1, forced expiratory volume in 1 second; AE, acute exacerbation; mMRC, modified British Medical Research Council; PA, *Pseudomonas aeruginosa*.

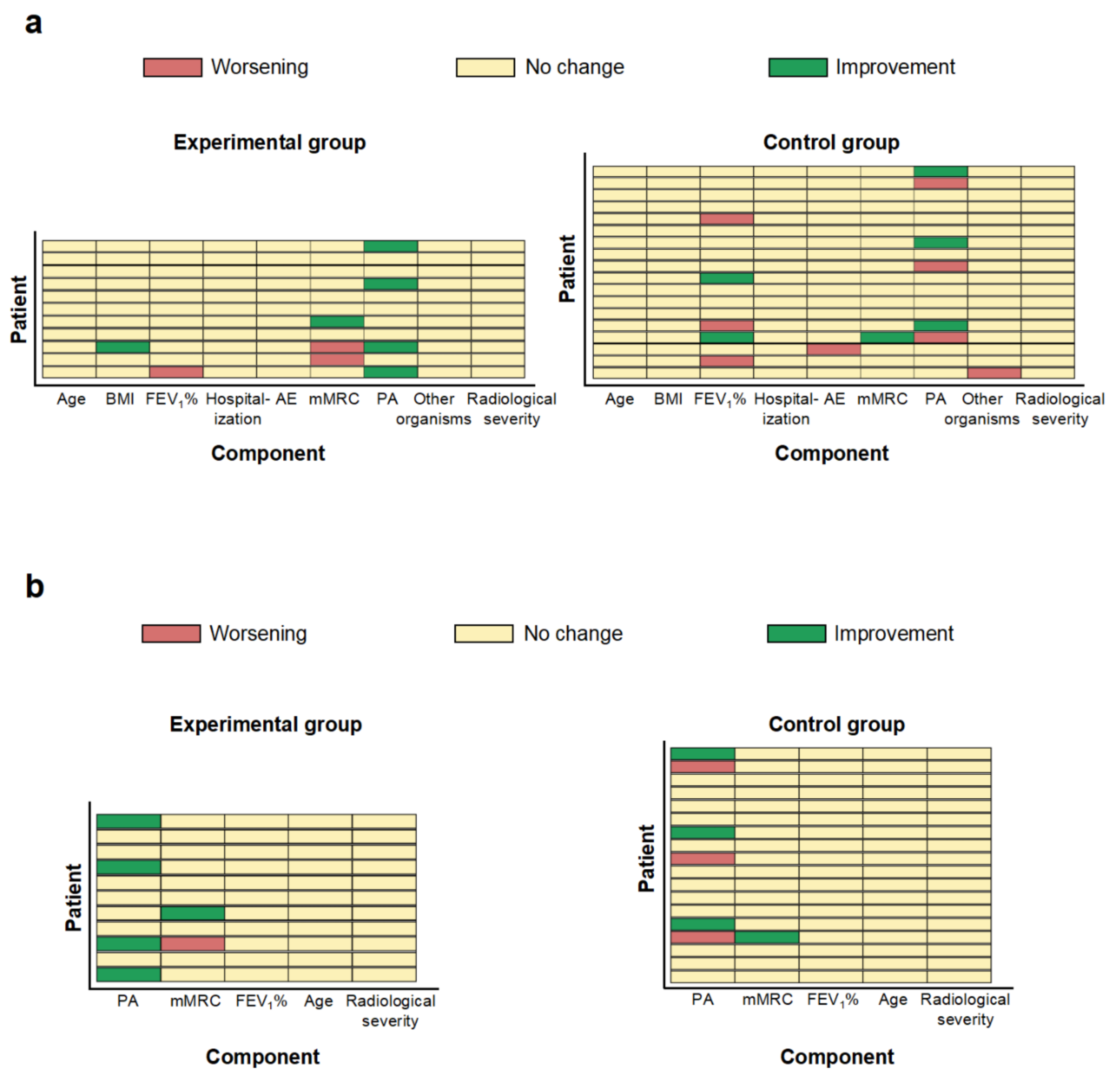

Fig. S8 Components of BSI and FACED scores at Week 24 for patients in two groups. Related to Figure 2 and Table S8. Components of BSI score (**a**) and FACED score (**b**) at Week 24 for patients in the experimental group (left) (n = 11) and the control group (right) (n = 18). Each row represents an individual patient. Green represents improvement, yellow no change, and red worsening, as compared with baseline values. BMI, body mass index; FEV_1, forced expiratory volume in 1 second; AE, acute exacerbation; mMRC, modified British Medical Research Council; PA, *Pseudomonas aeruginosa*.

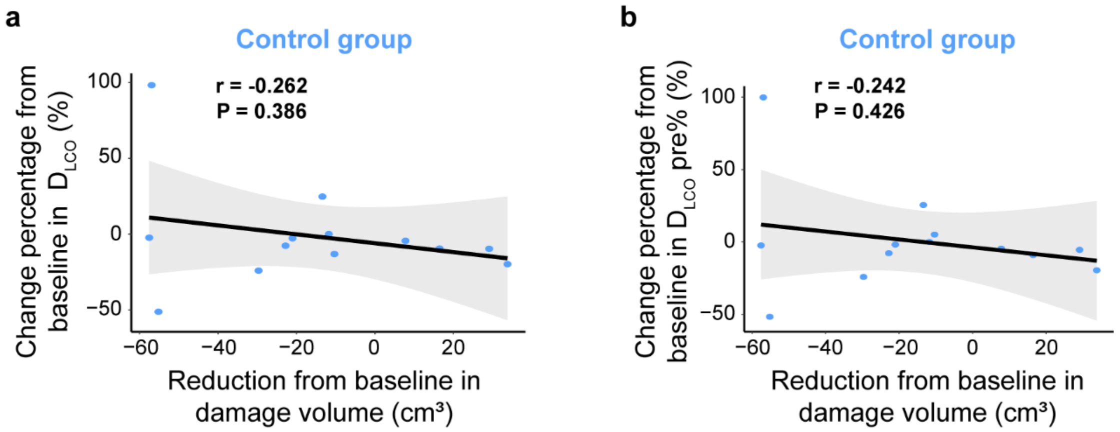

Fig. S9 Pearson correlations between the change from baseline to 24 weeks in the damaged volume and diffusing capacity in the control group. Related to Figure 3. Each dot represented an individual patient. Reduction from baseline = -1 × (change from baseline).

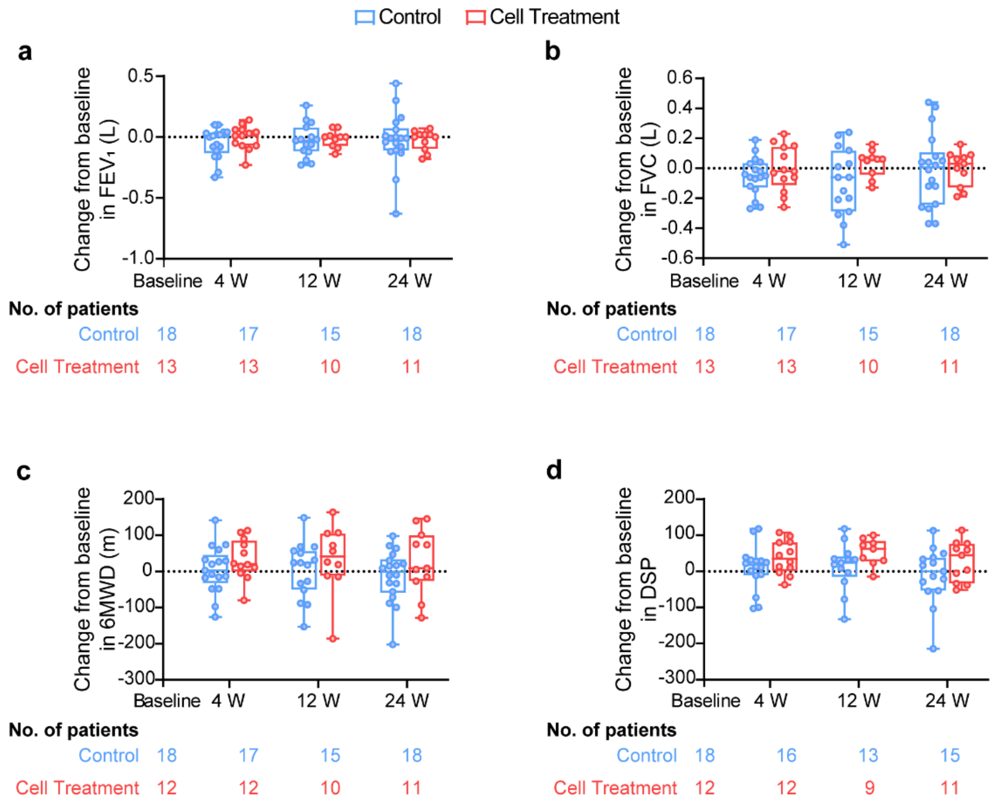

Fig. S10 Boxplot showing changes in FEV_1, FVC, 6MWD, and DSP at different time points. Related to Table S7. a, Changes in FEV_1 at Week 4, 12, and 24. **b**, Changes in FVC at Week 4, 12, and 24. **c**, Changes in 6MWD at Week 4, 12, and 24. **d**, Changes in DSP at Week 4, 12, and 24.

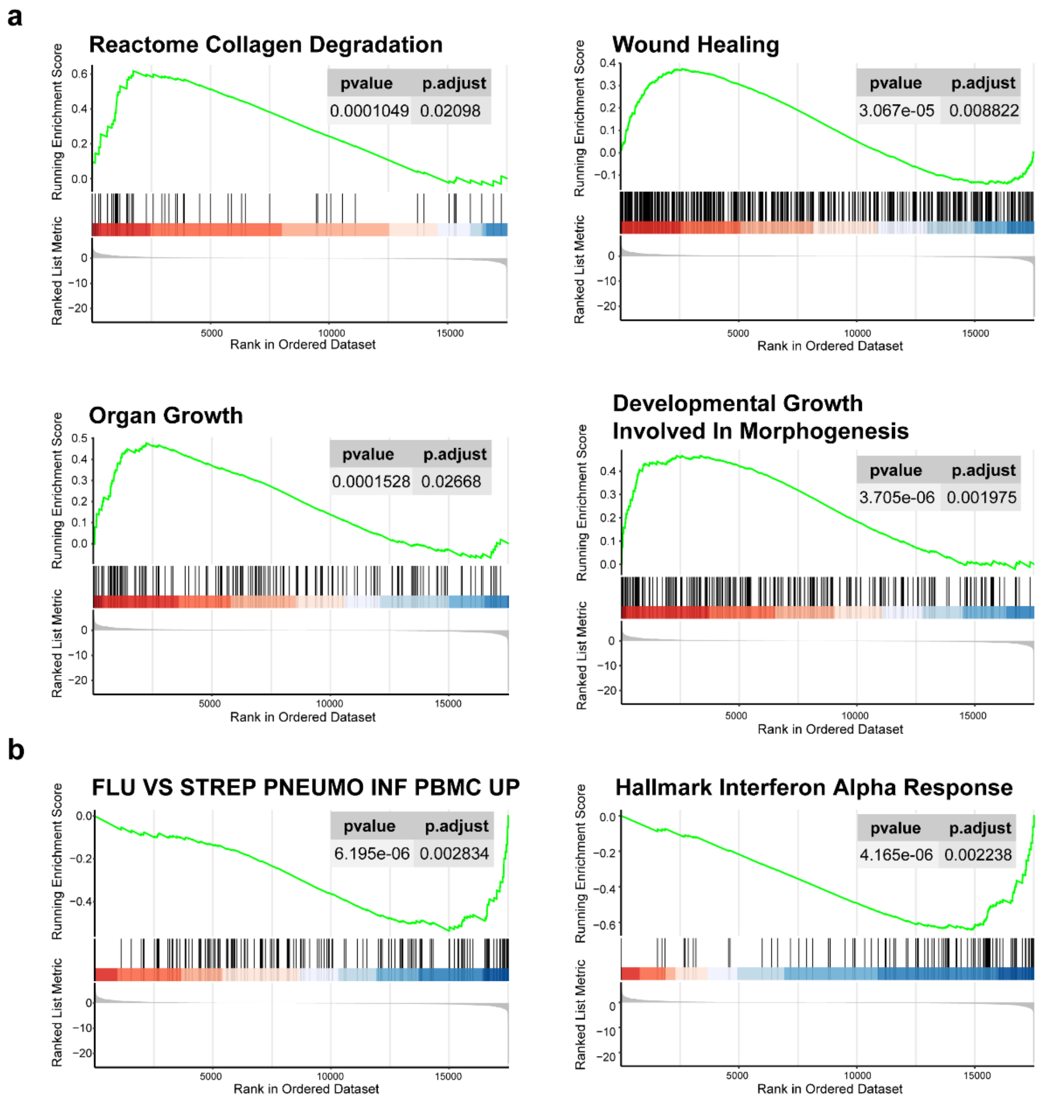

Fig. S11 Gene set enrichment analysis (GSEA) of RNA-Seq data from complete responsive (CR) and non-responsive (NR) progenitor cells. Related to Figure 4. a, b, GSEA showed that the CR (**a**) and NR (**b**) group was enriched in MSigDB gene sets compared to the NR and CR group (CR, n = 4; NR, n = 3).

Data S1: Clinical trial protocol of the study, related to the STAR Methods.

An Exploratory Study on Autologous Bronchial Basal Cell Transplantation for the Treatment of Bronchiectasis

Version 4.0 – March 2, 2020

Sponsor: Ruijin Hospital, Shanghai Jiao Tong University School of Medicine

Principal investigator: Jie-Ming Qu

Ethics Reference Number: 2018-10-5

ClinicalTrials. gov: NCT03655808

Confidentiality Statement:

The information contained in this research proposal is provided solely for the review of the researchers involved in this project, the ethics committee, and relevant institutions. It is strictly prohibited to disclose any information to third parties not associated with this research without the approval of the Principal Investigator (PI).

TABLE OF CONTENTS

1 INTRODUCTION 1
1.1 STUDY RATIONALE 2
1.2 BACKGROUND 4
1.2.1 Identification and characterization of bronchial basal cell 5
1.2.2 Collection, isolation, and culture of human bronchial basal cells 8
1.2.3 Mouse bronchial basal cell transplantation (ARDS model based on influenza virus) 10
1.2.4 Human bronchial basal cell transplantation (based on bleomycin-induced pulmonary fibrosis model) 11
1.2.5 Pilot clinical trial of autologous bronchial basal cell transplantation for the treatment of interstitial lung disease 12
1.2.6 Pilot clinical trial of autologous bronchial basal cell transplantation for the treatment of bronchiectasis 14
1.2.7 Other relevant studies 15
2 RESEARCH OBJECTIVES 17
3 STUDY DESIGN 17
3.1 OVERALL DESIGN 17
3.2 NUMBER OF PARTICIPANTS 17

3.3 RANDOMIZATION AND MASKING.......................... 17
4 STUDY POPULATION.. 18
4.1 DIAGNOSTIC CRITERIA 18
4.2 INCLUSION CRITERIA 18
4.3 EXCLUSION CRITERIA 19
4.4 REMOVING CRITERIA 20
4.5 WITHDRAWAL CRITERIA.. 21
5 STUDY INTERVENTIONS.. 23
5.1 INVESTIGATIONAL DRUG 23
5.2 ADMINISTRATION 23
5.2.1 Collection, separation, and culture of bronchial basal cells before treatment 23
5.2.2 Bronchial basal cell treatment 25
5.2.3 Transplantation treatment duration and course for bronchial basal cell therapy 26
5.3 PRIOR AND CONCOMITANT MEDICATIONS....... 26
5.4 HANDLING OF CASES INELIGIBLE FOR INCLUSION DURING THE CELL THERAPY PREPARATION PROCESS 27
5.5 HANDLING OF CASES WITH CELL CULTURE FAILURES 29
6 PACKAGING AND LABELING 30
6.1 PACKAGING SPECIFICATIONS............................. 30
6.2 PACKAGING REQUIREMENTS 30

6.3 LABELS 30
6.4 CELL ALLOCATION 30
6.5 PRODUCT STORAGE AND APPLICATION 31
6.6 DRUG MANAGEMENT 31
6.7 CODE ESTABLISHMENT 31
6.8 TRANSPORT QUALITY ASSURANCE MEASURES 32
7 SAFETY AND EFFICACY ASSESSMENT INDICATORS 33
7.1 SAFETY ASSESSMENTS 33
7.1.1 Physical and vital sign examination 33
7.1.2 Laboratory examinations 33
7.1.3 12-lead electrocardiogram (ECG) examination. 34
7.1.4 Fiber-optic bronchoscopy examination 34
7.1.5 Arterial blood gas analysis 35
7.2 EFFICACY ASSESSMENTS 36
7.2.1 Primary efficacy endpoints 36
7.2.2 Secondary efficacy endpoints 36
8 FOLLOW-UP PLAN 37
9 STUDY INTERVENTION DISCONTINUATION AND TERMINATION 39
9.1 CRITERIA FOR DISCONTINUATION 39
9.2 CRITERIA FOR TERMINATION 40

10 ADVERSE EVENTS AND SERIOUS ADVERSE EVENTS 40
10.1 DEFINITION OF ADVERSE EVENTS (AE) 40
10.2 DEFINITION OF SERIOUS ADVERSE EVENTS (SAE) 41
10.3 METHOD AND FREQUENCY OF AE DETECTION 42
10.3.1 Physical and vital signs examination 42
10.3.2 Laboratory tests 43
10.3.3 12-lead electrocardiogram (ECG) examination 43
10.3.4 Arterial blood gas analysis 44
10.3.5 Arterial blood gas analysis 45
10.4 RECORDING OF ADVERSE EVENTS AND SERIOUS ADVERSE EVENTS 45
10.5 ASSESSMENT OF ADVERSE EVENTS AND SERIOUS ADVERSE EVENTS 45
10.5.1 Severity determination 45
10.5.2 Causality determination 46
10.6 FOLLOW-UP OF ADVERSE EVENTS AND SERIOUS ADVERSE EVENTS 47
10.7 REPORTING OF SERIOUS ADVERSE EVENTS 47
10.8 ADVERSE REACTIONS AND TREATMENT METHODS 47

10.9 POTENTIAL RISKS AND MANAGEMENT STRATEGIES 48
11 DATA MANAGEMENT AND STATISTICAL ANALYSIS 50
11.1 DATA MANAGEMENT 50
11.1.1 Completion and Submission of CRF 50
11.1.2 Data Entry and Modification 50
11.1.3 Data Review 51
11.1.4 Data Lock 51
11.2 STATISTICAL ANALYSIS 51
11.2.1 Sample Size Determination 51
11.3 STATISTICAL METHODS 52
11.3.1 Proposed statistical methods 52
11.3.2 Primary efficacy endpoint 52
11.3.3 Statistical expression 53
11.4 STATISTICAL SOFTWARE AND GENERAL REQUIREMENTS 53
12 TRIAL MANAGEMENT 53
12.1 COMPLIANCE WITH GCP REQUIREMENTS 53
12.2 PROTECTION OF SUBJECT PRIVACY 53
12.3 QUALITY CONTROL AND ASSURANCE 54
12.3.1 Quality control 54
12.3.2 Quality assurance 55

12.4 SUBJECT CODING, RANDOM NUMBER TABLE, AND CRF PRESERVATION 56
13 ETHICAL CONSIDERATIONS 57
14 EXPECTED PROGRESS AND COMPLETION DATES OF CLINICAL TRIALS 57
15 MAIN REFERENCES 58

1 INTRODUCTION

Bronchiectasis is a chronic respiratory disease characterized by permanent and irreversible bronchial wall dilation and thickening. There has been a remarkable increase in its incidence and prevalence during the past 20 years. The latest statistics estimated that over 1.5% of women and 1.1% of men in the general population have physician-diagnosed bronchiectasis in China. Patients with bronchiectasis usually present with chronic cough and sputum production, and their clinical course is characterized by intermittent exacerbations, which can eventually develop into respiratory failure, causing loss of work ability and self-care ability, and even death. The condition worsens progressively and irreversibly, and the socioeconomic burden of the disease has also been increasing.

Current commonly used treatment methods in clinical practice include antibiotics, mucoactive agents, bronchodilators and corticosteroids, and airway clearance therapy. However, traditional treatments only provide symptomatic relief and fail to fundamentally solve the problem of lung structural damage. Lobes resection surgery is one of the treatments for bronchiectasis with poor prognosis, but this technology has relatively short development time, unclear indications and efficacy, and a high incidence of complications.

1.1 STUDY RATIONALE

Transplant therapy, which includes mature organ, tissue, and emerging cell transplant, is the main option for treating end-stage organ failure diseases so far. However, organ and tissue transplants come with significant disadvantages, such as the severe shortage of sources and obvious immune rejection. Allogeneic transplants, which are the most common type, often lead to varying degrees of immune rejection reactions. The use of immunosuppressants to treat this increases the risk of various opportunistic infections. Additionally, organ and tissue transplants require long surgical time, high technical requirements, and complex procedures. In the field of lung disease, the number of lung transplant surgeries performed in China in 2016 was less than 300, which was far from meeting the huge demand of patients.

Cell transplantation technology, particularly hematopoietic cell transplantation (transfusion and bone marrow transplantation), skin cell transplantation, and corneal cell transplantation, have been successfully used in clinical treatment and are increasingly valued by the medical community. These technologies provide a promising alternative to organ and tissue transplants and could help mitigate the current shortage of sources. Cell therapy has been utilized for the treatment of blood-related diseases, such as blood transfusion and bone

marrow transplantation since the 1970s. Over the years, it has been increasingly applied to other tissue and organ diseases, such as skin cell transplantation for burns, mesenchymal stem cell transplantation for liver failure and metabolic diseases, and limbal stem cell transplantation for corneal injuries. In cell therapy, different types of cells were cultured and used for distinct purposes, such as to enhance immune function, eliminate pathogens and tumor cells, promote tissue and organ regeneration, and aid in disease treatment.

Autologous stem/progenitor cell therapy has several advantages: 1) since the cells are sourced from the patient's own body, there is no risk of immune rejection; 2) there is no risk of tumorigenesis as the cells come from adult tissues and organs that are already part of the patient's body; 3) cells have plasticity and can actively divide and migrate to supplement apoptotic or necrotic cells of the same tissue or type in an appropriate environment; 4) the administrative procedure is simple and no complicated surgery is needed; and 5) cell transplantation can help tissue regeneration by multiple mechanisms: migrating to damaged tissue sites and differentiating into normal tissue cells, playing a role in repairing the damaged tissue, while at the same time, activating paracrine mechanisms by secreting various anti-inflammatory factors and inhibiting pro-inflammatory factor secretion. In conclusion, cell transplantation, with its advantages of good therapeutic effect, minimal side effects, personalized

and precision therapy, is currently being applied to the treatment of various diseases, including blood system diseases, tumors, diabetes, cardiovascular diseases, and nervous system diseases.

1.2 BACKGROUND

Recent high-level international scientific research has shown that a special population of **bronchial basal cells (also called $KRT5^+/P63^+$ distal airway stem cells or lung basal progenitor cells in some literature)** in the lungs can regenerate and repair damaged tissue (Hong, *et.al.*, *AJP*, 2004; Kumar, *et.al.*, *Cell*, 2011; Zuo, *et.al.*, *Nature*, 2015; Vaughan, *et.al.*, *Nature*, 2015), making them a promising type of "seed" cells for the treatment of lung tissue damage that cannot be naturally repaired by the body. These cells are located in the basal layer of the airway epithelium and specifically express the KRT5 and P63 antigens. They function as adult tissue stem/progenitor cells in lung, and are relatively active in cell division and migration, could produce cells to replace other types of epithelial cells that have been dismissed. They have plasticity and can directly repair the structure of bronchi and alveoli epithelium.

Such bronchial basal cells can be obtained by bronchoscopic brushing, and isolated, purified, and extensively

expanded by appropriate methods. It has been proven that bronchial basal cell transplantation can directly repair damaged lungs in experimental animal models. Since bronchial basal cells are derived from autologous tissue, there is no immune rejection issue. Also, because bronchial basal cells are derived from adult tissue organs and are themselves part of the body, there is no risk of tumor formation. In the animal experiment, even when 100 times the human dose of bronchial basal cells was administered *via* the trachea in animals, there was no tumor formation observed.

1.2.1 IDENTIFICATION AND CHARACTERIZATION OF BRONCHIAL BASAL CELL

Recent studies have revealed the existence of bronchial basal cells located in the basal layer of the bronchi in the lungs. These cells express the KRT5 marker gene, as well as the P63 gene. Although they are relatively rare in mouse lungs, they are widespread in human lungs. Please refer to the image below for more details:

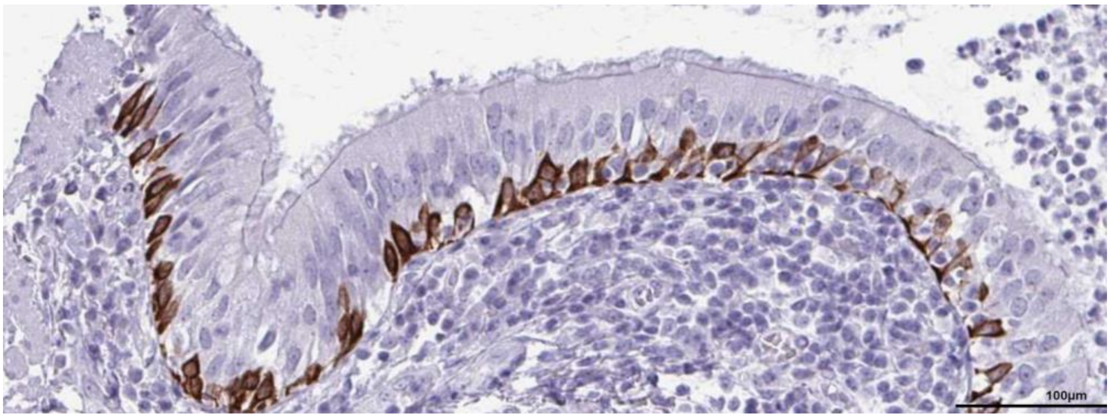

Human bronchial basal cells (KRT5 IHC staining)

Importantly, we have observed a significant proliferation of $KRT5^+$ bronchial basal cells in the bronchial basal layer in the injury area of ARDS patient lungs, which has begun to form bronchiole and air sac-like structures as shown in the figure below. This suggests the potential role of bronchial basal cells in lung repair.

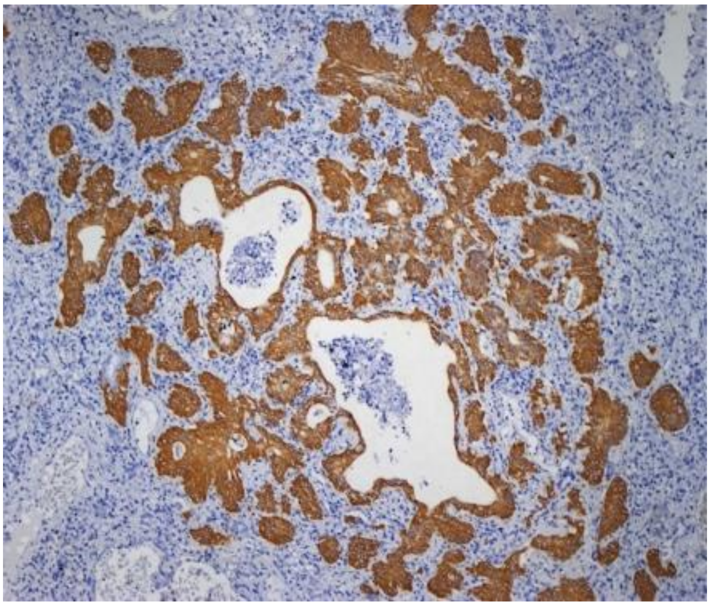

To prove the potential of bronchial basal cells to differentiate into mature alveolar and bronchial epithelial cells, Professor Wei Zuo's team utilized genetic lineage tracing to track the proliferation, migration, and differentiation of bronchial basal cells in mice following influenza virus infection. This demonstrated that these cells are indeed capable of repairing lung tissue. The results are shown in the figure below (Zuo, *et.al.*, *Nature*, 2015).

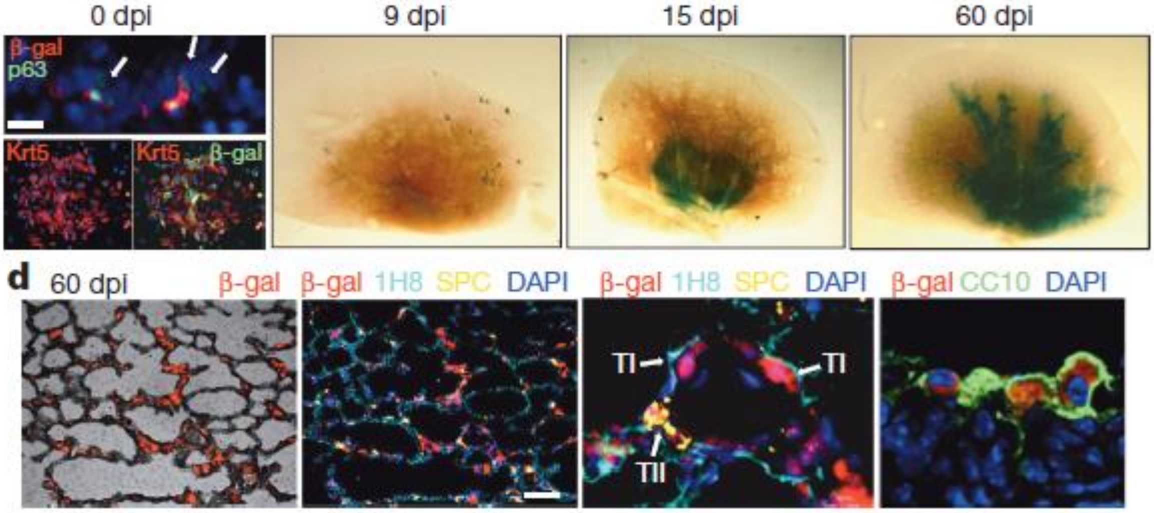

Further animal experiments have also demonstrated that if the activated bronchial basal cells were genetically eliminated using a diphtheria toxin receptor (DTR) in mice (DTR + DTox group), not only could lung function not be repaired, but tissue fibrosis could also occur. It can be inferred that maintaining a sufficient number of bronchial basal cells in the body is essential for repairing the lungs and inhibiting the occurrence of lung fibrosis, as shown in the figure below:

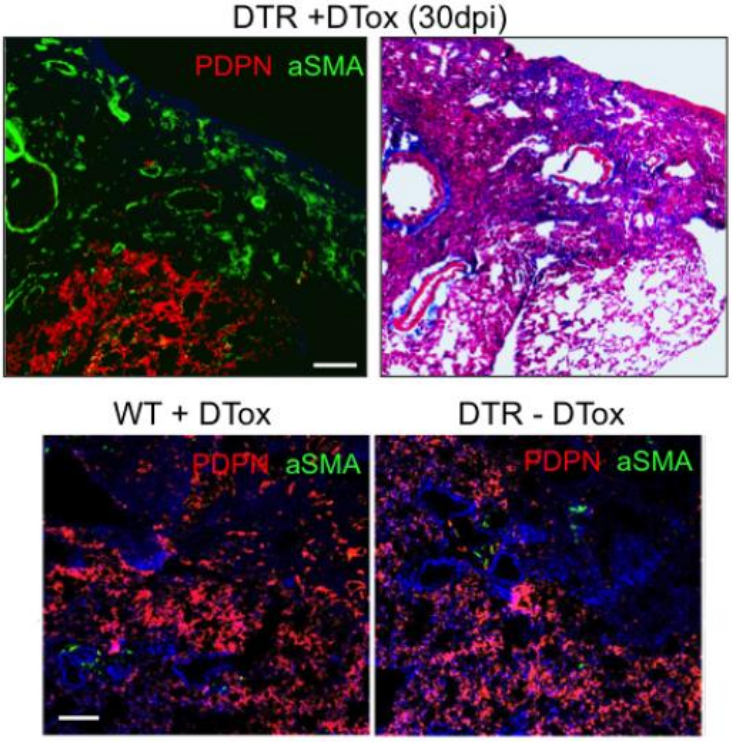

1.2.2 COLLECTION, ISOLATION, AND CULTURE OF HUMAN BRONCHIAL BASAL CELLS

Our team obtained small amounts of bronchial basal cells through bronchoscopic brushing. The obtained tissue was washed and digested into single-cell suspension, which was then cultured using a patented technology that simulates the basal layer environment *in vitro* using a combination of growth factors. This system selectively amplified bronchial basal cells, while other types of mature epithelial cells and fibroblasts could not grow and would naturally undergo apoptosis. After a period of expansion, bronchial basal cells could be stored in liquid nitrogen cell banks for long-term preservation. Before being used

in subjects, bronchial basal cells underwent a series of strict tests, including microbial contamination detection, cell morphology, cell viability, genetic characteristics, and KRT5 marker gene detection, *etc*. The bronchial basal cells obtained from brushing and P_0 culture are shown in the figure below:

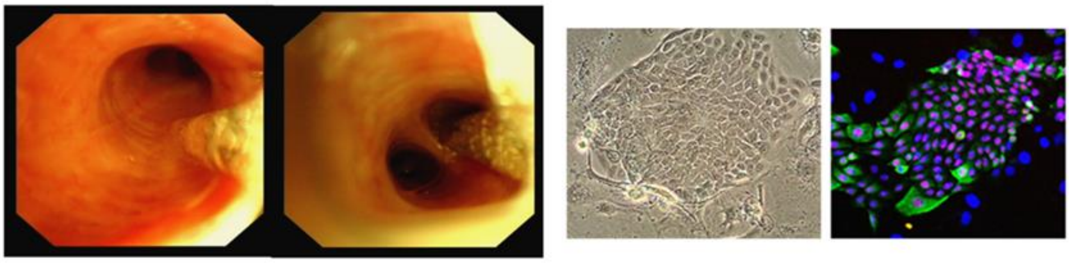

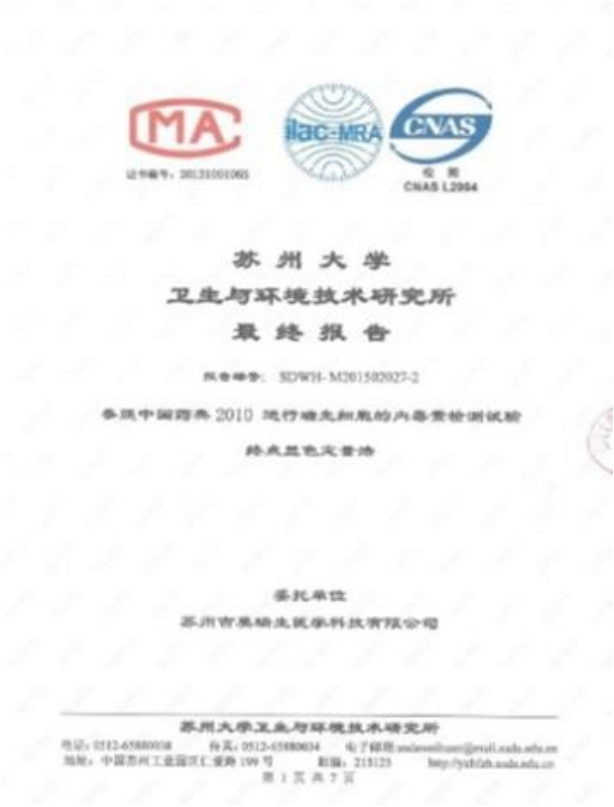

苏州大学

卫生与环境技术研究所

最终报告

CMA/CNAS QC Certification

1.2.3 MOUSE BRONCHIAL BASAL CELL TRANSPLANTATION (ARDS MODEL BASED ON INFLUENZA VIRUS)

In the mouse model of ARDS induced by the H_1N_1 influenza virus, we genetically labeled bronchial basal cells with blue LacZ and then transplanted them into the mouse lungs *via* the trachea. We observed that the donor cells integrated extensively into the recipient's lungs and differentiated into mature alveoli and bronchial structures. It is worth noting that the transplantation of bronchial basal cells into the lungs of healthy mice without injury was completely unsuccessful, which to some extent guarantees the relative safety of transplantation. The figure below shows the data (Zuo, *et.al.*, *Nature*, 2015):

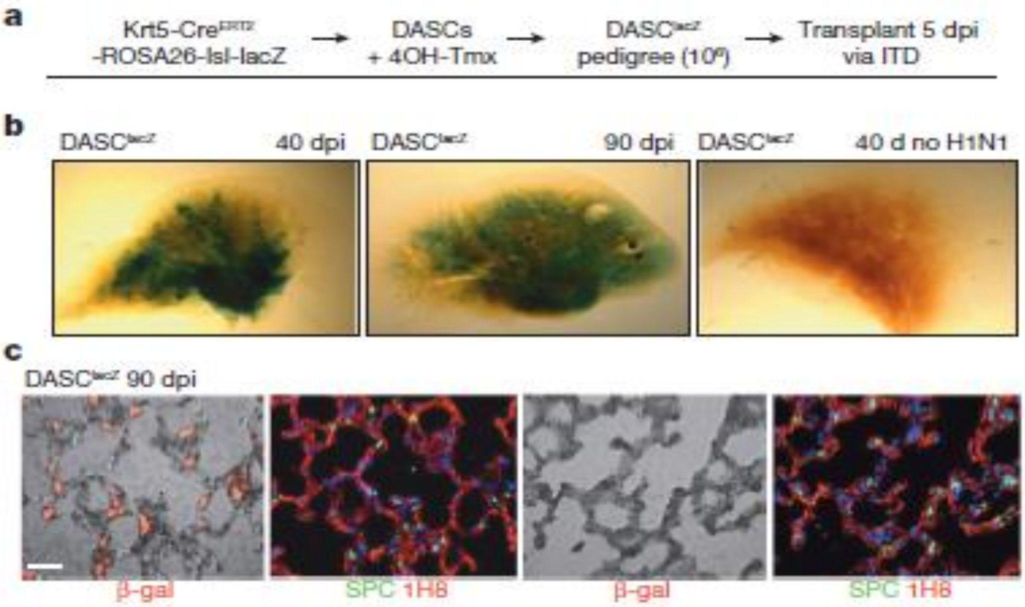

For ARDS mice whose lung injuries could not be naturally repaired due to the lack of bronchial basal cells, the basal cell

transplantation helped restore their morphological structure and function (blood oxygen saturation). At the same time, transplanting bronchial basal cells in lung-injured mice treated with bleomycin significantly increased their survival time. The figure below shows this:

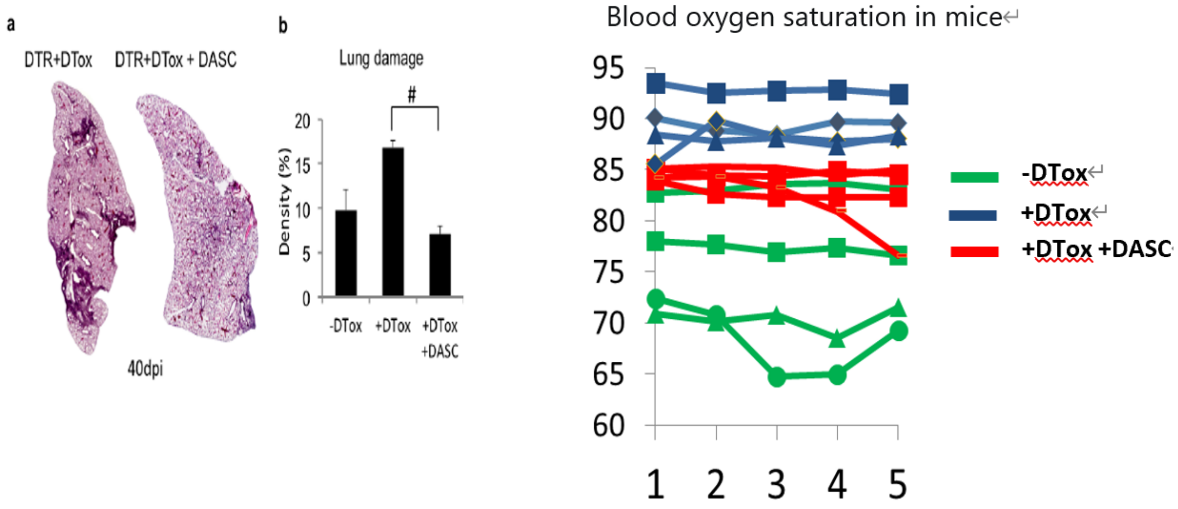

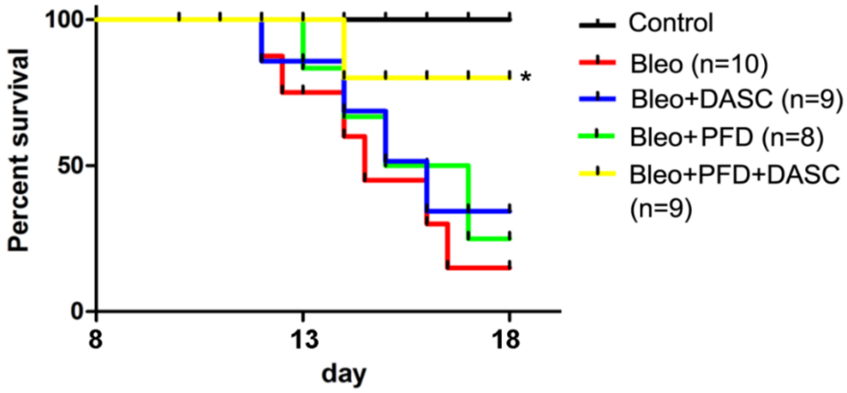

1.2.4 HUMAN BRONCHIAL BASAL CELL TRANSPLANTATION (BASED ON BLEOMYCIN-INDUCED PULMONARY FIBROSIS MODEL)

In the mouse model of lung injury induced by bleomycin, human bronchial basal cells (marked with GFP fluorescence)

transplanted into the injured area of the recipient's lungs were observed to integrate extensively three weeks after transplantation. They differentiated into mature human alveolar epithelium and bronchial structures. The newly generated human bronchiole-alveolar epithelium recruited surrounding capillaries, reconstructing functional units with gas exchange ability. Importantly, by rebuilding the epithelial tissue structure, the transplantation of bronchial basal cells effectively reduced the proliferation and activation of fibroblasts (α-SMA positive) in the lungs. The transplanted bronchial basal cells and their differentiated offspring in the body survived almost indefinitely. The process is shown in the figure below:

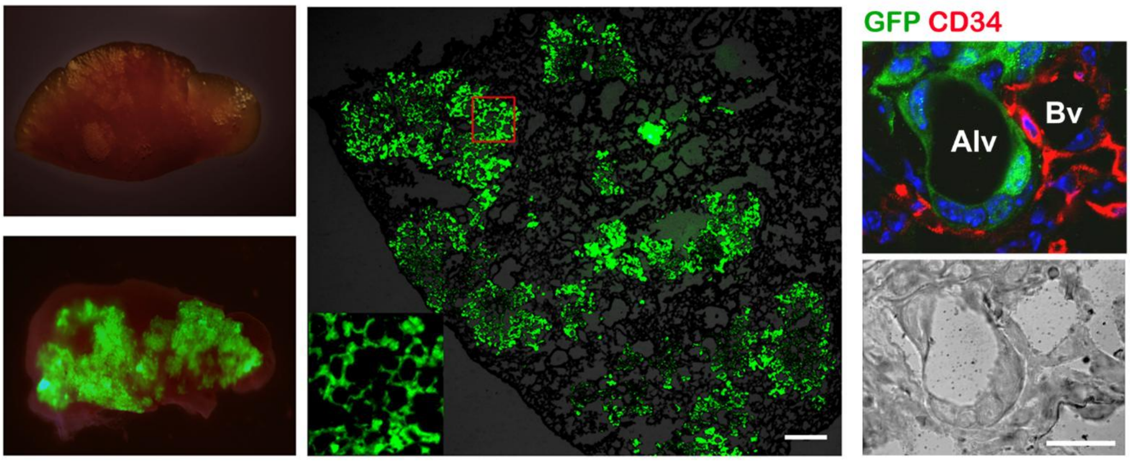

1.2.5 PILOT CLINICAL TRIAL OF AUTOLOGOUS BRONCHIAL BASAL CELL TRANSPLANTATION FOR THE TREATMENT OF INTERSTITIAL LUNG DISEASE

In 2016, Regend Therapeutics collaborated with Shanghai East Hospital to investigate the potential of autologous bronchial basal cell transplantation therapy. The treatment was administered to a 59-year-old male who was diagnosed with interstitial lung disease at the First Affiliated Hospital of Guangzhou Medical University in 2015. In April 2016, this subject underwent autologous bronchial basal cell transplantation therapy at Shanghai East Hospital. Three weeks post-transplantation, CT imaging revealed significant improvement in interstitial lesions (as depicted in the figure below). Various lung function indicators, such as FVC (forced vital capacity), FEV_1 (forced expiratory volume in one second), and D_{LCO} (diffusing capacity of the lungs for carbon monoxide), demonstrated varying degrees of enhancement. Additionally, the 6-minute walk test significantly improved, and symptoms of wheezing exhibited significant improvement.

Before transplantation 3 weeks post transplantation

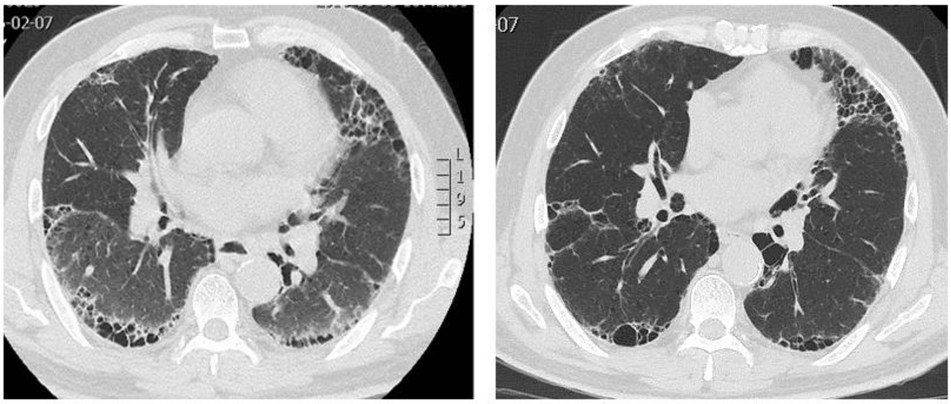

As of December 2016, a total of five cases for autologous bronchial basal cell transplantation therapy for interstitial lung disease had been completed at Shanghai East Hospital. The results showed exceptionally high levels of safety and promising preliminary efficacy.

1.2.6 PILOT CLINICAL TRIAL OF AUTOLOGOUS BRONCHIAL BASAL CELL TRANSPLANTATION FOR THE TREATMENT OF BRONCHIECTASIS

After obtaining approval from the ethics committee of the Southwest Hospital of the Third Military Medical University of PLA, two patients with bronchiectasis (one of whom had comorbid COPD) received autologous bronchial basal cell transplantation therapy in our department in 2016. Three months after treatment, we observed significant improvements in various pulmonary function indicators, including FVC, VC MAX, FEV_1, and D_{LCO}, as well as a decrease in C-reactive protein levels and a significant increase in the 6-minute walk test. Furthermore, according to the subjects' self-reports, their symptoms were significantly relieved, as shown in the table below:

Days post transplantation	FVC (%Pred)			FEV1(%Pred)			VC MAX (%Pred)			TLC-SB (%Pred)		
	-1	30	90	-1	30	90	-1	30	90	-1	30	90
Patient 1	69.4	58.3	79.5	36.6	31.9	47.5	74.1	62.1	81.5	75.9	120.6	88.2
Pateint 2	24.3	61.2	62.4	20.8	34.6	33.3	24.8	62.2	60.4	22.2	67.6	71.9

Days post transplantation	RV-SB (%Pred)			FRC-SB (%Pred)			DLCOc/VA (%Pred)			6MWT (m)		
	-1	30	90	-1	30	90	-1	30	90	-1	30	90
Patient 1	94.5	245.0	116.0	71.9	194.3	89.7	128.2	136.3	120.2	453	504	558
Pateint 2	15.7	73.1	101.0	40.7	112.0	120.3	14.9	103.9	97.3	399	453	468

1.2.7 OTHER RELEVANT STUDIES

Currently, there are several clinical studies registered on ClinicalTrials.gov investigating the use of autologous and allogeneic mesenchymal stem cell transplantation therapy, including adipose-derived stem cells and bone marrow-derived mesenchymal stem cells, for the treatment of COPD. Nine clinical trials have been registered so far (NCT02645305, NCT02216630, NCT00683722, NCT01110252, NCT02161744, NCT02348060, NCT02041000, NCT02412332, NCT01559051), with six being conducted in the United States, two in Brazil, and one in Vietnam. Two small-sample studies using allogeneic mesenchymal stem cell transplantation therapy for COPD in the United States and Brazil have been completed and reported high safety levels with almost no adverse reactions, but they did not show significant improvement in lung function or other therapeutic endpoints. In contrast, our preliminary attempts at autologous bronchial basal cell transplantation therapy in our hospital have demonstrated significant improvements in lung function indicators such as FEV_1, FVC, MMEF, MVV, 6-minute

walk test (6MWT), and St. George's Respiratory Questionnaire (SGRQ) score. These results suggest that by fundamentally repairing the damaged lung structure of COPD patients, autologous bronchial basal cell transplantation therapy may help to restore lung function.

2 RESEARCH OBJECTIVES

The objective of this study is to evaluate the safety and efficacy of autologous bronchial basal cell transplantation in the treatment of bronchiectasis.

3 STUDY DESIGN

3.1 OVERALL DESIGN

This trial is a randomized, single-blind, controlled pilot study.

3.2 NUMBER OF PARTICIPANTS

A total of 76 participants are expected to be enrolled and randomly assigned to the control or cell treatment group at a 1:1 ratio.

3.3 RANDOMIZATION AND MASKING

Eligibility patients will be assigned according to a random number table, with sequentially numbered in a 1:1 ratio generated by computer, to receive either B-ACT + autologous bronchial basal cell transplantation therapy (cell treatment group) or B-ACT therapy (control group). The opaque sealed envelope method will be used to conceal the allocation sequence. Both patients and investigators, except for the bronchoscopy

operators, remain masked to the treatment assignment for the duration of the study. That is, only the investigators who perform the bronchoscopy are unblinded. The non-blinded investigators should not disclose any blind information to other investigators, participants, or clinic staff.

4 STUDY POPULATION

4.1 DIAGNOSTIC CRITERIA

The diagnosis will be based on the 2019 British Thoracic Society (BTS) guidelines for bronchiectasis.

4.2 INCLUSION CRITERIA

- 18~75 years, outpatients with chronic cough, sputum production, and a clinical diagnosis of bronchiectasis (confirmed by chest high-resolution computed tomography [HRCT] scan);
- Remaining clinically stable (respiratory symptoms not significantly exceeding the daily variations, and in the absence of acute exacerbation of bronchiectasis or acute upper respiratory tract infection within the previous 2 weeks);
- D_{LCO} % predicted < 80%;

- Being capable of doing pulmonary function tests;
- Being eligible for bronchoscopy and willing to receive autologous bronchial basal cell transplantation therapy;
- Patients or their family members voluntarily participated in the study and signed the informed consent.

4.3 EXCLUSION CRITERIA

- Pregnant or lactating women or women of childbearing age who were planning to conceive;
- Positive serological tests for hepatitis B virus (HBV), hepatitis C virus (HCV), human immunodeficiency virus (HIV), or syphilis (HBV carriers and patients with stable chronic hepatitis B could be accepted if titers of HBV DNA < 500 IU/mL or copies < 1000 copies/mL; patients with curative hepatitis C were eligible if HCV RNA tests were negative)
- Malignant tumors;
- Co-existing active pulmonary tuberculosis, pulmonary embolism, pneumothorax, multiple huge bullae, uncontrolled asthma, acute exacerbation of chronic bronchitis, or extremely severe chronic obstructive pulmonary disease (COPD);
- A history of severe systemic diseases (*i.e.*, poorly controlled diabetes, myocardial infarction, unstable angina pectoris, liver cirrhosis, acute glomerulonephritis)

- Leukopenia (WBCs < 4 × 10^9 /L) or agranulocytosis (WBCs < 1.5 × 10^9 /L or neutrophils < 0.5 × 10^9 /L) for any cause;
- Significant kidney dysfunction (Cr being 1.5 times higher than the upper limit of normal);
- Significant liver dysfunction (ALT, AST, total bilirubin > 2 times of the upper limit of normal);
- A history of mental disorders, or suicide risk, or epilepsy, or other central nervous system disease;
- Clinically significant arrhythmia (*i.e.*, ventricular tachycardia, frequent supraventricular tachycardias, atrial fibrillation, atrial flutter, second- or third-degree atrioventricular block);
- A history of alcohol or drug abuse;
- Participated in other clinical trials within 3 months before screening;
- Subjects with poor compliance, difficult completing the study;
- Any other conditions that might increase the risk of subjects or interfere with the clinical trial.

4.4 REMOVING CRITERIA

- Participants who do not meet the inclusion criteria or meet the exclusion criteria.

- Participants who are unwilling or unable to continue participating in this trial.
- Participants who are unable to evaluate efficacy due to treatment interruption.
- Participants who interrupt treatment due to pregnancy.

4.5 WITHDRAWAL CRITERIA

Subject-Requested Withdrawal:

According to the provisions of the informed consent form, participants have the right to withdraw from the clinical study at any time, or if a participant, without explicitly withdrawing from the clinical study, no longer undergoes medication and testing and becomes lost to follow-up (also considered withdrawal or dropout). Participants who meet the inclusion criteria for entry into the clinical study, regardless of when or for what reason they withdraw before completing the specified observation and follow-up periods, are considered dropout cases.

For participants who withdraw, the researcher should clearly state the reasons for withdrawal and the withdrawal time, and should (or as much as possible) conduct appropriate observation and evaluation of the withdrawn participants, complete the specified withdrawal assessment, and fill out the corresponding records. For those who withdraw due to adverse reactions or

abnormal laboratory findings, tracking should continue until the adverse reactions disappear or the laboratory findings return to normal/baseline levels. Properly preserving trial data related to dropout cases is crucial, and a comprehensive analysis set should be compiled for them.

Researcher-Requested Subject Withdrawal Conditions:

- The Medical Ethics Committee deems it necessary to stop.
- Inability to collect a sufficient number of bronchial basal cells according to the planned protocol, or failure to successfully amplify autologous basal cells due to reasons such as drug-resistant bacterial infection, or the quality of amplified autologous basal cells is unsuitable for clinical researchers.
- Occurrence of a severe adverse event making the participant unsuitable to continue the trial.
- Occurrence of severe other concurrent diseases during the clinical study.
- Use of any anticancer drugs during the clinical study.
- Poor compliance of the participant, who no longer undergoes cell transplantation therapy or testing before completing the entire clinical study, cannot adhere to the planned clinical study, including not adhering to prescribed

medications, or any other factor that may affect efficacy observation.

- Participation in other clinical trials during the clinical study.
- Acute worsening of relevant symptoms in the participant.
- Other situations deemed necessary by the researcher to withdraw from the study.

5 STUDY INTERVENTIONS

5.1 INVESTIGATIONAL DRUG

Autologous bronchial basal cells

Dosage: 1~3 × 10^6 cells/kg body weight, which can be adjusted according to the severity of bronchiectasis.

5.2 ADMINISTRATION

5.2.1 COLLECTION, SEPARATION, AND CULTURE OF BRONCHIAL BASAL CELLS BEFORE TREATMENT

Using the bronchoscopic brush method, a small amount of tissue is collected and bronchial basal cells are isolated from the 3~5th level bronchi of the human lung after B-ACT completed. The obtained tissue is washed and enzymatically digested to form a single-cell suspension, which is then cultured. The basal cell culture system is a patented technology of the sponsor

(Regend Therapeutics Co., Ltd.), which uses a combination of special growth factors and composite materials *in vitro* to simulate the basal environment and selectively expand bronchial basal cells, while other types of terminally differentiated epithelial cells and fibroblasts cannot survive and naturally die. After a period of expansion, bronchial basal cells can be stored in a liquid nitrogen cell bank for long-term preservation. Before being used for subjects, bronchial basal cells need to undergo a series of rigorous tests, including identity, purity, sterility, endotoxin, viral contamination, bovine serum albumin (BSA) remaining and antibiotic remaining, *etc*.

Strict quality control of the transplanted bronchial basal cells, including relevant testing to ensure that the cells meet the quality standards:

- Cell viability ≥ 90%;
- Expression of marker KRT5;
- Purity greater than 90%;
- No bacterial or fungal contamination;
- No mycoplasma contamination;
- No bovine virus contamination;
- Endotoxin content lower than 0.1 EU/mL;
- Cells have a karyotype of 46XX or 46XY;

- Potential for differentiation into alveolar-like structures or HOPX-positive cells.
- Gentamicin residues in cell suspension are less than 5.4 ppb.

5.2.2 BRONCHIAL BASAL CELL TREATMENT

After locating the lesion through chest CT, the subject will be placed in the supine position and receive local anesthesia. Cell suspension is pre-warmed to approximately 37 °C 15 minutes before use, and then kept in a syringe for later use. A fiberoptic bronchoscope will be used to perform B-ACT. After the bronchoalveolar lavage is completed, the lavage fluid in the affected area of the bronchial lobe or segment will be aspirated as much as possible. Six lung segments with the most severe lesions are selected by the team of doctors before bronchoscopy according to CT results. After lavage and when the oxygen saturation of patients reaches > 92%, 5 mL of the cell suspension will be slowly and gently pushed into each lung segment *via* the working channel of the bronchoscope with a 20 mL syringe in around 30 seconds, and the severely damaged lung segment may be injected multiple times. After the successful injection of bronchial basal cells, the feeding tube will be removed first, followed by the fiberoptic bronchoscope. Non-invasive positive pressure ventilation treatment may be added if necessary. Then

allow the subject to maintain a flat position for 2 hours after transplantation. The subject should not drink water for 2 hours after surgery and is advised to minimize coughing. If necessary, oral codeine may be given.

5.2.3 TRANSPLANTATION TREATMENT DURATION AND COURSE FOR BRONCHIAL BASAL CELL THERAPY

The duration of bronchial basal cell transplantation treatment is determined by the completion time of cell expansion. Following the completion of bronchial basal cell tissue sampling, the subsequent processes include cell isolation, cryopreservation, culture, and expansion. Generally, these procedures take 4~8 weeks. Upon completion of the expansion, various quality tests are conducted in the production workshop. The product is then released, transported to the cell therapy unit, and the cell therapy is completed.

Currently, the course of bronchial basal cell transplantation treatment consists of a single session, meaning the treatment is administered only once.

5.3 PRIOR AND CONCOMITANT MEDICATIONS

In principle, subjects can continue to use medications they were taking before the start of this treatment to exclude any interference from sudden discontinuation of medication on the

study results. Conventional bronchiectasis medications can be used for treatment. For subjects who were already receiving conventional bronchiectasis medication before the screening, the dosage should remain stable from the start of the screening period throughout the entire study. For subjects who are recently diagnosed and have not received any treatment, they may begin using conventional bronchiectasis medications from the start of the screening period and maintain a stable dosage throughout the entire study. For all concomitant medications, the researcher should record the details on the Concomitant Drug Use page of the Case Report Form (CRF), including the reason for medication/treatment, administration/treatment methods, and start and end dates.

5.4 HANDLING OF CASES INELIGIBLE FOR INCLUSION DURING THE CELL THERAPY PREPARATION PROCESS

If a participant, originally meeting the inclusion and exclusion criteria, becomes ineligible for cell therapy between the completion of cell collection and the initiation of cell therapy (usually a period of 4~8 weeks), the participant will be considered disqualified, and the preparation of cell therapy will be temporarily halted. The cells that have been cultured, either completed or partially completed, will be cryopreserved

according to the relevant regulations of the production workshop. After being disqualified, the participant may undergo observation and intervention for a period. When their physical condition permits, they can participate in screening again. If they meet the inclusion criteria, there is no need to recollect cells; they can directly enter the trial, reschedule the treatment time, and use the cells after resuscitation, cultivation, and testing. Participants can undergo multiple screenings until the end of the study. If, until the end of the study, a participant has not met the inclusion criteria, they will be excluded, and the cells will be either destroyed or cryopreserved for an extended period at the participant's request.

Considerations regarding the standards or range of various pre-cell therapy examinations for participants mainly involve the researcher further verifying the inclusion and exclusion criteria before the participant undergoes cell therapy. If the participant still meets the inclusion criteria, does not meet the exclusion criteria, and there are no special circumstances, the researcher, after comprehensive assessments, deems the participant suitable for cell therapy, and the treatment proceeds as normal. If the participant does not meet the inclusion criteria or meets the exclusion criteria, the treatment will be temporarily halted (cells that have been cultured will be cryopreserved, and treatment will be given at a later date). For instance, if there is significant progression of the participant's condition, worsening of

pulmonary inflammation, higher risk during bronchoscopy, follow-up CT (as required or treatment interval exceeding 8 weeks) reveals concurrent lung cancer with a life expectancy of less than 1 year, or the anticipated frequent risk of acute exacerbation after cell therapy is high, the researcher, after comprehensive assessment, deems the participant unsuitable for cell therapy. These situations can be considered as criteria or a range where treatment is not acceptable.

5.5 HANDLING OF CASES WITH CELL CULTURE FAILURES

If, during the cell culture process before treatment, it is discovered that the cells cannot be cultured, or there is abnormal cell proliferation, or quality inspection reveals that the cells do not meet quality requirements, the reasons need to be investigated. The technical department should design a response plan, arrange for the participant to undergo resampling, and conduct cell culture again. Cells that have been cultured (if found to be non-compliant) should be destroyed according to relevant regulations.

If the re-culturing is successful, the participant will proceed with normal enrollment in the clinical trial. If re-culturing still fails, the participant will be excluded from the clinical trial.

Note: Preliminary research results from the applicant indicate that the success rate of the first cell culture is higher than 90%, and the success rate of two consecutive cultures is higher than 95%.

6 PACKAGING AND LABELING

6.1 PACKAGING SPECIFICATIONS

30 mL per package.

6.2 PACKAGING REQUIREMENTS

The product will be packaged in sealed sterile bags.

6.3 LABELS

Autologous Bronchial Basal Cells
(For clinical research use by the subject only)

Batch: __________________ ID: _____________________________
Indication: Bronchiectasis
Specification: 30 mL
Color: White or transparent
Cell count:
Dosage and administration: After suspension in saline, the cells will be locally introduced into the lungs at a dose of 1-3 × 10^6/Kg/person.
Storage: 2-8 °C
Shelf life: 12 hours
Date of manufacture: As indicated in the package insert
Notes: These cells are sterile products and should be used strictly under the guidance of a physician. If you have any questions, please consult your physician promptly. Unused cells and packaging need to be collected.
Regend Therapeutics Co., Ltd.

6.4 CELL ALLOCATION

The cells will be strictly used for autologous treatment. Once the cells are prepared, they will be immediately transported to the clinical facility by a designated individual and received by responsible personnel. The cells will be used by the managing physician of the subject.

6.5 PRODUCT STORAGE AND APPLICATION

Storage conditions: 2~8 ^{o}C

The cells will be shipped to Ruijin Hospital in an ice box with a real-time monitoring and alarm device for temperature, and delivered to the airway using a bronchoscope.

6.6 DRUG MANAGEMENT

A designated individual will be responsible for managing the cells. The cells will be counted after each use, and any unused cells and packaging will be collected and returned.

6.7 CODE ESTABLISHMENT

The patient identification number will be consistent with the cell preparation number. The numbering will start from the patient's admission to the hospital, and all related documents will use the same numbering system.

6.8 TRANSPORT QUALITY ASSURANCE MEASURES

1) Firstly, ensure that the real-time temperature recorder is in good condition (with sufficient battery and calibrated within the last year). Set the temperature alarm's upper limit to 8.1 ^{o}C and the lower limit to 1.9 ^{o}C.

2) The packaged cellular preparations (including internal and external packaging) are placed in a temperature-controlled box balanced at 2-8 ^{o}C for transportation. Simultaneously, an opened real-time temperature recorder is placed in the temperature-controlled box to monitor the temperature throughout the transportation. Set the recording interval of the temperature recorder to 15 minutes, meaning it will automatically record the temperature inside the temperature-controlled box every 15 minutes. Ensure uninterrupted temperature detection, continuous recording, data storage, and alerts for exceeding limits throughout the transportation.

3) Upon arrival of the cellular preparations at the clinical institution, personnel should inspect the packaging integrity of the cellular preparations. Confirm that the temperature displayed on the temperature recorder is within the range of 2-8 ^{o}C. Additionally, the transportation personnel should promptly retrieve the temperature recorder to verify the temperature conditions inside the temperature-controlled box throughout the transportation and complete relevant records.

4) Within 3 hours of cellular transportation to the clinical institution, institution personnel conduct a release inspection of the cells to exclude the influence of the transportation process on the quality of the stem cells.

7 SAFETY AND EFFICACY ASSESSMENT INDICATORS

7.1 SAFETY ASSESSMENTS

The main safety endpoint is the incidence of adverse events. The following assessments will be conducted during the follow-up:

7.1.1 PHYSICAL AND VITAL SIGN EXAMINATION

- Physical and vital sign examination include height, weight, medical history, *etc*.

7.1.2 LABORATORY EXAMINATIONS

- Complete blood count: red blood cell count, white blood cell count, hemoglobin, platelets, white blood cell differential count, mean corpuscular volume, hematocrit, and mean corpuscular hemoglobin concentration.
- Urinalysis: urine pH, specific gravity, protein, glucose, ketones, occult blood, white blood cells, and urobilinogen.

- Biochemical analysis: alanine aminotransferase (ALT), aspartate aminotransferase (AST), alkaline phosphatase (ALP), gamma-glutamyltransferase (GGT), uric acid (URIC), total protein (TP), albumin (ALB), creatine kinase (CK), lactate dehydrogenase (LDH), total bilirubin (TBIL), blood urea nitrogen (BUN), creatinine (Cr), blood glucose (Glu), sodium ion (Na^+), potassium ion (K^+), chloride ion (Cl^-).

7.1.3 12-LEAD ELECTROCARDIOGRAM (ECG) EXAMINATION

- The 12-lead ECG examination should record heart rate, rhythm, PQ or PR interval, QRS interval, QT interval (uncorrected), and QTc (QT/RR1/2), and provide an overall evaluation (normal, clinically insignificant abnormalities, clinically significant abnormalities, requiring further explanation). The signed original ECG will be archived at the study center, and the ECG examination results will be recorded on the CRF.

7.1.4 FIBER-OPTIC BRONCHOSCOPY EXAMINATION

1) Preoperative Preparation: Thoroughly understand the participant's medical history, conduct a physical examination, perform CT scans, and conduct laboratory tests. Explain the

purpose and significance of the examination, addressing concerns. Ensure a fasting period of at least 4 hours. Administer intramuscular atropine 0.5 mg 30 minutes before, with sedatives for anxious individuals.

2) Local Anesthesia: Combine 2% lidocaine throat spray with inhalation anesthesia until a sensation of obstruction in the throat.

3) Procedure Steps: Position the participant supine, inserting the flexible bronchoscope through the nasal passages. Observe tracheal mucosal folds, cartilaginous rings, epiglottis, bronchi openings, mucosal smoothness, color, presence of abnormalities, and record details.

4) Specimen Collection: If abnormalities like new growths are identified, perform a bronchoscopic biopsy for specimen collection.

5) Postoperative Care: Restrict oral intake for 2 hours, allowing eating or drinking only after anesthesia effects dissipate. Explain potential blood-tinged sputum. Administer antibiotics for postoperative fever and provide oxygen therapy for breathlessness and hypoxemia.

7.1.5 ARTERIAL BLOOD GAS ANALYSIS

- pH, P_aCO_2, P_aO_2, the concentration of HCO_3^-.

7.2 EFFICACY ASSESSMENTS

7.2.1 PRIMARY EFFICACY ENDPOINTS

- The change from baseline in D_{LCO} at Week 4, 12, and 24 (after treatment).

7.2.2 SECONDARY EFFICACY ENDPOINTS

- The changes from baseline in other pulmonary function parameters except D_{LCO}, including FEV_1, FVC, FEV_1/FVC, MMEF, and MVV, at Week 4, 12, and 24
- The changes from baseline in the 6-minute walking distance (6MWD) and the composite index (distance-saturation product, DSP) at Week 4, 12, and 24
- The changes from baseline in the modified medical research council (mMRC) chronic dyspnea scale at Week 4, 12, and 24
- The changes from baseline in the SGRQ score at Week 4, 12, and 24
- The changes from baseline in bronchiectasis severity index (BSI) score and FACED score at Week 4, 12, and 24
- Imaging of lung by HRCT at Week 4, 12, and 24

8 FOLLOW-UP PLAN

All subjects must sign an informed consent form before screening.

The study physician will provide the subjects with complete and truthful information about the background, objectives, trial design, direct and indirect benefits that the subjects can obtain, potential risks, and any other relevant information related to this clinical trial, in a quiet and private environment. The subjects will be allowed to think independently and discuss with the physician, ask any questions they have, and receive help to fully understand all the information. After the subjects make a voluntary decision to participate, they and the study physician will sign the informed consent form simultaneously.

Please refer to the table below for details on the specific study procedures:

Project	Screening (collection)	Baseline	4 weeks	12 weeks	24 weeks
**Sign of informed consent**	X				
**Inclusion / exclusion criteria screen**	X	X			
**Medical history inquiry**	X	X			
**Symptom inquiry**	X	X	X	X	X
**Physical examination**	X	X	X	X	X

**Vital signs examination**	X	X	X	X	X
**Blood routine test**	X	X	X	X	X
**Blood pregnancy test[1]**		X			
**Urine routine test**		X	X	X	X
**Blood biochemical test**		X	X	X	X
**Blood coagulation test**	X	X			
**Creatine kinase test**		X	X	X	X
**Serological test of syphilis, HBV, HCV, HIV**	X				
**Electrocardiogram**	X	X	X	X	X
**Chest HRCT**	X		X	X	X
**Bronchoscopic brushing**	X				
**Cell transplantation**		X			
**Arterial blood gas analysis**		X	X	X	X
**Pulmonary function testing**	X	X	X	X	X
**6MWT and DSP**		X	X	X	X
**SGRQ**		X	X	X	X
**BSI score**		X	X	X	X
**FACED score**		X	X	X	X
**Combined medicine**	X	X	X	X	X
**Adverse event evaluation**	X	X	X	X	X

Note: The day of cell transplantation is considered day 0 of the study; all follow-up procedures for baseline will be completed

before bronchial basal cell infusion except for adverse events, and bronchoscopy will only be performed during the non-acute phase. The follow-up time may vary by approximately 7 days before and after the scheduled time.

[1]Female subjects (postmenopausal women who have completed one year of menopause are not required to participate).

9 STUDY INTERVENTION DISCONTINUATION AND TERMINATION

9.1 CRITERIA FOR DISCONTINUATION

The clinical trial must be discontinuation if the following situations occur:

- If a subject experiences intolerable adverse events or serious adverse events, and the investigator determines that the risk of continued participation in this trial is greater than the benefit to the subject, the trial must be terminated. Appropriate treatment measures should be taken, and the subject should be included in the full analysis set for safety analysis.
- If the subject's condition worsens after treatment, the subject should be included in the efficacy and safety analysis for the protocol set.

- If the investigator or sponsor terminates the study for any reason, the reason for termination should be submitted to the ethics committee.
- Once a subject withdraws from this trial, they cannot re-enter the study.

9.2 CRITERIA FOR TERMINATION

The clinical trial will be ended when the following situations occur:

- Enough subjects have been recruited;
- Follow-up has been completed for the last subject recruited;
- Statistical analysis has been completed for all safety and efficacy indicators for all subjects.

10 ADVERSE EVENTS AND SERIOUS ADVERSE EVENTS

10.1 DEFINITION OF ADVERSE EVENTS (AE)

Any unexpected and unfavorable medical occurrence related to any medical intervention in the study, regardless of its association with bronchial basal cell therapy, is considered an adverse event (AE). AE includes clinically significant laboratory abnormalities that indicate damage to a disease and/or organ.

All observed or subject-reported adverse events should be recorded on the adverse events page of the CRF.

For each adverse event, the time of occurrence, severity, duration, management measures, and outcome of the event should be described, and analysis should be conducted to determine the causal relationship with the study drug and whether it meets the criteria for a serious adverse event.

The analysis of the association between adverse events and bronchial basal cell therapy should take into account the following factors:

- Whether there is a reasonable temporal sequence between the adverse event and the time of bronchial basal cell therapy;
- Whether the clinical or pathological manifestations of the adverse event are consistent with known knowledge of bronchial basal cell therapy;
- Whether the adverse event can be explained by the original disease, subject's factors, or environmental factors.

10.2 DEFINITION OF SERIOUS ADVERSE EVENTS (SAE)

A serious adverse event (SAE) is defined as an adverse event that results in any of the following:

- Death;
- Life-threatening;
- Requires hospitalization or prolongation of existing hospitalization;
- Results in persistent or significant disability/incapacity;
- Results in a congenital anomaly/birth defect;
- Cancer.

If a subject experiences a serious adverse event during the trial, regardless of whether it is related to bronchial basal cell therapy, the investigator should take appropriate treatment measures immediately to ensure the subject's safety.

10.3 METHOD AND FREQUENCY OF AE DETECTION

While observing the efficacy, closely monitor for adverse events or unforeseen toxic side effects. Safety indicators are set in the observation index, as follows:

10.3.1 PHYSICAL AND VITAL SIGNS EXAMINATION

Physical and vital signs examination including height, weight, medical history, *etc*.

10.3.2 LABORATORY TESTS

- Blood routine: red blood cell count, white blood cell count, hemoglobin, platelet, white blood cell differential count, mean red cell volume, hematocrit, and mean corpuscular hemoglobin concentration.
- Urine routine: urine pH, specific gravity, protein, urine sugar, ketone bodies, occult blood, white blood cells, and urinary bilirubin.
- Blood biochemistry: alanine aminotransferase (ALT), aspartate aminotransferase (AST), alkaline phosphatase (ALP), gamma-glutamyl transferase (GGT), uric acid (URIC), total protein (TP), albumin (ALB), creatine kinase (CK), lactate dehydrogenase (LDH), total bilirubin (TBIL), blood urea nitrogen (BUN), creatinine (Cr), blood glucose (Glu), sodium ion (Na^+), potassium ion (K^+), and chloride ion (Cl^-).

10.3.3 12-LEAD ELECTROCARDIOGRAM (ECG) EXAMINATION

The 12-lead ECG examination should record heart rate, rhythm, PQ or PR interval, QRS interval, QT interval (uncorrected), and QTc (QT/RR1/2) and give an overall evaluation (normal, clinically insignificant abnormality, clinically

significant abnormality, need further explanation). The signed original ECG will be archived at the study center, and the ECG examination result will be recorded on the CRF.

10.3.4 ARTERIAL BLOOD GAS ANALYSIS

Fiber-optic bronchoscopy examination:

1) Preoperative Preparation: Thoroughly understand the participant's medical history, conduct a physical examination, perform CT scans, and conduct laboratory tests. Explain the purpose and significance of the examination, addressing concerns. Ensure a fasting period of at least 4 hours. Administer intramuscular atropine 0.5 mg 30 minutes before, with sedatives for anxious individuals.

2) Local Anesthesia: Combine 2% lidocaine throat spray with inhalation anesthesia until a sensation of obstruction in the throat.

3) Procedure Steps: Position the participant supine, inserting the flexible bronchoscope through the nasal passages. Observe tracheal mucosal folds, cartilaginous rings, epiglottis, bronchi openings, mucosal smoothness, color, presence of abnormalities, and record details.

4) Specimen Collection: If abnormalities like new growths are identified, perform a bronchoscopic biopsy for specimen collection.

5) Postoperative Care: Restrict oral intake for 2 hours, allowing eating or drinking only after anesthesia effects dissipate. Explain potential blood-tinged sputum. Administer antibiotics for postoperative fever and provide oxygen therapy for breathlessness and hypoxemia.

10.3.5 ARTERIAL BLOOD GAS ANALYSIS

- pH, P_aCO_2, P_aO_2, the concentration of HCO_3^-.

10.4 RECORDING OF ADVERSE EVENTS AND SERIOUS ADVERSE EVENTS

For any adverse events and serious adverse events that occur during the study period, their symptoms, severity, onset time, duration, treatment measures, and outcomes should be recorded in the CRF. The relationship between the event and the study cells should be evaluated, and the researcher should record, sign, and date the information in detail.

10.5 ASSESSMENT OF ADVERSE EVENTS AND SERIOUS ADVERSE EVENTS

10.5.1 SEVERITY DETERMINATION

- Mild adverse event: The subject has symptoms, but the reaction is slight and tolerable, does not affect normal

activities, and the symptoms are transient and self-resolving.

- Moderate adverse event: The symptoms affect the subject's normal daily life and persist for a longer duration.
- Severe adverse event: The subject's body function is impaired, leading to loss of normal work and life ability, and the symptoms persist for an even longer duration.

10.5.2 CAUSALITY DETERMINATION

The relationship between adverse events and study cells can be classified into the following categories:

- Definitely related: There is evidence of using the study drug, the occurrence of adverse events is reasonably related to cell therapy, and it is more reasonable to explain the adverse events with cell therapy than other reasons.
- Probably related: There is a reasonable correlation between the occurrence of adverse events and cell therapy in time. Adverse events can be explained by other reasons.
- Possibly unrelated: There is evidence of using cell therapy, and the adverse events may be better explained by other reasons.

- Unrelated: No cell therapy was conducted, or there is no correlation between cell therapy and the occurrence of adverse events, or there are other clear causes of adverse events.
- Unable to determine.

10.6 FOLLOW-UP OF ADVERSE EVENTS AND SERIOUS ADVERSE EVENTS

Subjects experiencing adverse events will be followed up *via* telephone interview once per quarter, and the recovery of the subjects will be recorded in detail.

10.7 REPORTING OF SERIOUS ADVERSE EVENTS

In the event of a serious adverse event, the clinical research unit must take immediate measures to protect the safety of the subjects and report to the Drug Supervision Administration, sponsor, and ethics committee within 24 hours. The investigator should sign and date the report. The sponsor will ensure compliance with all legal and regulatory reporting procedures.

10.8 ADVERSE REACTIONS AND TREATMENT METHODS

Adverse reactions should be anticipated before the operation, and timely treatment should be provided if adverse reactions occur. The patients should receive corresponding symptomatic treatment.

When an adverse event occurs, the observing physician may decide whether to discontinue the observation according to the patient's condition. For cases where treatment is stopped due to adverse reactions, a follow-up investigation should be conducted, and the management process and results should be recorded in detail.

10.9 POTENTIAL RISKS AND MANAGEMENT STRATEGIES

- Risks associated with bronchoscopy: The common complications of bronchoscopy include anesthesia accidents, bleeding, pneumothorax, laryngospasm, hypoxemia, infection, postoperative fever, and other unexpected events. Currently, clinical practices in bronchoscopy are highly mature and exhibit good safety. This study involves only sputum suction and injection in specific lung segments, and the occurrence of complications is very low.
- Risk Monitoring for Anesthesia-Related Events: 1) Anesthesia physicians must conduct thorough

preoperative visits, explaining procedures and formulating suitable plans. 2) Verify equipment functionality before anesthesia and ensure preparedness for any patient receiving anesthesia. 3) Monitor vital signs closely, adhere to a double-check system for medications, and stay with the patient throughout anesthesia.4) Prepare for intravenous anesthesia, securing loose teeth and ensuring gentle tracheal intubation. 5) Conduct postoperative follow-ups within 48 hours, promptly reporting any issues to senior physicians for intervention.

- Other possible risks during bronchial basal cell therapy: Regend Therapeutics follows GMP to establish bronchial basal cell production and quality systems. Trained personnel, especially the manager, ensure compliance, preventing contamination risks with clear roles, regular training, and effective reporting mechanisms.
- Multiple organ dysfunction: Due to the rich capillary network in the lungs, bronchial basal cells, if excessively absorbed into the bloodstream, may lead to multi-organ dysfunction. Our method, delivered through the trachea, minimizes the risk compared to intravenous administration, reducing the likelihood of organ dysfunction to less than 0.01%.

- Emergency management of massive hemoptysis: Massive hemoptysis is a life-threatening complication of bronchiectasis, defined as a single episode exceeding 200 ml or a total of 500 mL within 24 hours. It can lead to suffocation. Preventing asphyxiation is the primary focus. Ensure airway patency, improve oxygenation, and stabilize hemodynamics. For lesser amounts of hemoptysis, reassure the patient and have them rest on the affected side. In cases of asphyxiation, place the patient in a 45° head-down position, clear blood clots from the mouth, and gently tap the healthy side of the back to facilitate blood drainage. If these measures are ineffective, prompt endotracheal intubation or, if necessary, a tracheostomy should be performed.

11 DATA MANAGEMENT AND STATISTICAL ANALYSIS

11.1 DATA MANAGEMENT

11.1.1 COMPLETION AND SUBMISSION OF CRF

The investigator is responsible for completing CRF for each eligible case. Completed CRFs will be reviewed by the trial sponsor and archived.

11.1.2 DATA ENTRY AND MODIFICATION

A designated person, appointed by the trial sponsor, will enter CRF data into the database. Any modifications to the data should follow the CRF modification requirements.

11.1.3 DATA REVIEW

Following the completion of data entry and verification, a designated person appointed by the trial sponsor will review the data and make the final determination of the analysis population.

11.1.4 DATA LOCK

Data can be locked once the following conditions are met:

- All data has been entered into the database;
- All queries have been resolved;
- The analysis population has been defined and determined.

11.2 STATISTICAL ANALYSIS

11.2.1 SAMPLE SIZE DETERMINATION

A sample size of 76 subjects is not based on any statistical considerations. The sample size is based on the clinical consideration to provide safety and efficacy information with the need to minimize exposure to subjects in a pilot study.

11.3 STATISTICAL METHODS

11.3.1 PROPOSED STATISTICAL METHODS

- Descriptive Statistics:

Outliers: Statistical and professional analysis is used to determine whether to include outliers.

Missing data: For individual subjects with missing primary efficacy data, the method of imputation is determined based on statistical and professional judgment.

Descriptive statistics: n, mean, standard deviation (SD), median, Q1, Q3, and range (minimum and maximum), *etc*.

- Inferential Statistics:

Continuous data: Unpaired Student's t-test and Mann-Whitney U test.

Count data: Chi-square test and Fisher's exact test.

11.3.2 PRIMARY EFFICACY ENDPOINT

The primary efficacy endpoint in the study is the change of D_{LCO} after therapy. D_{LCO} data will be expressed as both absolute value (mmol/min/kPa) or % of predicted (%). The difference between the cell treatment and control groups of the changes in D_{LCO} from baseline to Week 4, 12, and 24, is tested using the Mann-Whitney U test, and the median differences are calculated using the Hodges-Lehmann estimation.

11.3.3 STATISTICAL EXPRESSION

- Tables are mainly used to present the results and should be self-explanatory, with a title, caption, and number of cases.
- Results of repeated measures data are presented in tables, with statistical graphs attached to increase readability.
- Analyses are presented with two-sided P values, with the level of significance set at 0.05.

11.4 STATISTICAL SOFTWARE AND GENERAL REQUIREMENTS

All statistical analysis and diagramming are performed by SPSS (version 25.0) and GraphPad (version 9.0).

12 TRIAL MANAGEMENT

12.1 COMPLIANCE WITH GCP REQUIREMENTS

This clinical trial fully complies with the Good Clinical Practice (GCP) guidelines and is strictly conducted according to the specified requirements.

12.2 PROTECTION OF SUBJECT PRIVACY

This clinical trial respects the privacy of subjects, strictly protects subject privacy, and prevents leakage of subject information.

12.3 QUALITY CONTROL AND ASSURANCE

12.3.1 QUALITY CONTROL

- Determination of the study protocol: The clinical research protocol is discussed and negotiated by all researchers participating in this clinical trial. After reaching a consensus, it is submitted to the ethics committee for approval.
- Quality control measures for the laboratory: the sponsor has standardized testing indicators, standard operating procedures, and quality control procedures.
- Qualifications of researchers: Researchers participating in clinical trials must have professional expertise, qualifications, and capabilities in clinical research. After qualification review, personnel requirements are relatively fixed.
- Pre-trial training: Pre-trial training for researchers is conducted to ensure full understanding and awareness of the clinical research protocol and its specific indicators.

The entire clinical trial process should strictly follow the relevant operating norms.

- Measures to ensure subject compliance: Detailed information on the huge benefits of successful cell therapy is provided to subjects, which may help prevent the pain of future loss of lung function, and subject compliance is monitored.
- The abnormal judgment criteria for laboratory examinations are based on the normal reference range of the inspection unit.
- All observation results and findings in the clinical study should be verified to ensure the reliability of the data and ensure that all conclusions in the clinical study are derived from original data. Corresponding data management measures are taken in the clinical study and data processing stages.
- Based on the original observation records of the subjects, researchers ensure that the data are entered correctly (consistent with the actual situation of the subjects), complete (without omissions), clear (neat handwriting and easy to identify), and timely on the CRF.

12.3.2 QUALITY ASSURANCE

- The sponsor appoints a monitor to ensure the protection of the rights and interests of subjects in the clinical study, the accuracy and completeness of research records and report data, and ensure that the study follows the approved protocol and relevant regulations.
- All observation results and findings in the trial should be verified to ensure the reliability of the data and ensure that all conclusions in the clinical trial are derived from original data. Quality control is used at every stage of data processing to ensure that all data is reliable and processed correctly.
- The sponsor performs strict quality testing of cell preparation, provides a cell out-of-factory testing report, and ensures the quality of the cells.

12.4 SUBJECT CODING, RANDOM NUMBER TABLE, AND CRF PRESERVATION

The researcher should keep all study materials, including confirmation of all subjects participation (effective cross-check of different recording data, such as original records in hospitals), all original informed consent forms signed by the subjects, all case observation forms, and detailed records of cell distribution and

use. The researcher should keep the clinical study materials for at least 5 years after the end of the study.

13 ETHICAL CONSIDERATIONS

This clinical study will adhere to the Helsinki Declaration (2008 version) and relevant regulations and guidelines for the management of cellular clinical research in China. Before the commencement of the clinical trial, the research center ethics committee must approve the study protocol.

Before each participant is enrolled in the study, the investigating physician is responsible for providing complete and comprehensive written information about the purpose, procedures, and potential risks of the study to the participant or their designated representative. Participants should be informed that they have the right to withdraw from the study at any time. Each participant must be provided with an informed consent form before being enrolled in the study. The investigating physician is responsible for ensuring that each participant signs the informed consent form before entering the clinical trial and keeping it in the study records.

14 EXPECTED PROGRESS AND COMPLETION DATES OF CLINICAL TRIALS

1) Research Start Date: The implementation of this protocol will commence after obtaining approval from the Ethics Committee.

2) Midterm Clinical Coordination Meeting: The timing for convening the midterm clinical coordination meeting will be determined based on the progress and completion status of the clinical study.

3) Clinical Study Completion Date: The clinical study is expected to be completed within 36 months from the initiation of the research.

4) Data Collection, Statistical Analysis, and Summary of Clinical Study: The collection, statistical analysis, and summarization of clinical study data will be completed within 6 months after the completion of the study and receipt of the statistical analysis report.

15 MAIN REFERENCES

Hong KU, Reynolds SD, *et al*. Basal cells are multipotent progenitor capable of renewing the bronchial epithelium. *American Journal of Pathology* 2004. 164:577-588.

Zuo W, Zhang T, *et al*. p63+Krt5+ distal airway stem cells are essential for lung regeneration. *Nature* 2015 517,616-620.

Vaughan A, Brumwell, *et al*. Lineage-negative progenitor mobilize to regenerate lung epithelium after major injury. *Nature* 2015 517, 621-625.
